# Genomic Analysis of a Mycobacterium Bovis Bacillus Calmette-Guérin Strain Isolated from an Adult Patient with Pulmonary Tuberculosis

**DOI:** 10.1371/journal.pone.0122403

**Published:** 2015-04-13

**Authors:** Xuming Li, Liping Chen, Yongqiang Zhu, Xia Yu, Jun Cao, Rui Wang, Xinyan Lv, Jin He, Aizhen Guo, Hairong Huang, Huajun Zheng, Siguo Liu

**Affiliations:** 1 Division of Bacterial Diseases, State Key Laboratory of Veterinary Biotechnology, Harbin Veterinary Research Institute, Chinese Academy of Agriculture, Harbin 150001, China; 2 Stake Key Laboratory of Agricultural Microbiology, College of Life Science and Technology, Huazhong Agricultural University, Wuhan, Hubei 430070, China; 3 Shanghai-MOST Key Laboratory of Health and Disease Genomics, Chinese National Human Genome Center at Shanghai, Shanghai 201203, China; 4 National Clinical Laboratory on Tuberculosis, Beijing Key Laboratory on Drug-resistant Tuberculosis Research, Beijing Tuberculosis and Thoracic Tumor Institute, Beijing Chest Hospital, Capital Medical University, Beijing101149, China; 5 Laboratory of Medical Foods, Shanghai Institute of Planned Parenthood Research, 2140 Xie-Tu Road, Shanghai 200032, China; University of Delhi, INDIA

## Abstract

For years, bacillus Calmette-Guérin (BCG) has served as the unique vaccine against tuberculosis and has generally been regarded as safe. However, a clinical strain labeled 3281 that was isolated from a TB patient was identified to be BCG. Via the combination of next-generation sequencing (NGS) and comparative genomic analysis, unique 3281 genetic characteristics were revealed. A region containing the dnaA and dnaN genes that is closely related to the initial chromosome replication was found to repeat three times on the BCG Pasteur-specific tandem duplication region DU1. Due to the minimum number of epitopes in BCG strains, 3281 was inferred to have a high possibility for immune evasion. Additionally, variations in the virulence genes and predictions for potential virulence factors were analyzed. Overall, we report a pathogen that has never previously been thought to be pathogenic and initial insights that are focused on the genetic characteristics of virulent BCG.

## Introduction

During the 20 years since the WHO declared that tuberculosis (TB) is a global public health emergency, great efforts have been made to control and eradicate this diseaseworldwide. Globally, the TB mortality rate has fallen by 45% since 1990. Although considerable progress has been made in these years,an estimated 8.6 million individuals stilldevelop TB, and 1.3 million die from the disease every year[1]. As one of the “three killers” of humans, TB remains a current major global health problem.Furthermore, one-third of the world population is latently infected with *Mycobacterium tuberculosis* (MTB), which makes the eradication of this this disease more difficult[2].

With the development of genomics and high-throughput sequencing technology, scientists have sought to disclose the “secret garden” of TB via the use of genomic methods[3,4]. H37Rv is a laboratoryvirulent MTB strain whose genome was the first to be completely sequenced, and it has typically been used as areference strain in comparative genomic research. The sole available TB vaccine, bacillus Calmette-Guérin (BCG), was derived from *Mycobacterium bovis*(*M*.*bovis*); the virulence of this mycobacterium was attenuated in the laboratory via cultivation on potatoglycerol medium, and this vaccine can only supply sufficient protection for children. However, this vaccine is incapable ofproviding the same efficacy for adolescents and adults[1,5]. Furthermore, the continual process of the subculturingof BCG in laboratories around the world has led to the generation of daughter strains, and the protective efficacies against these strains has been shown to varyacross laboratories and epidemiological investigations[6–8].To define the molecular basis of the attenuation of BCGs and the variation among daughter strains, comparative genomics research has been performed. Comparisons of BCG to *M*. *bovis*revealed that several genes associated with virulence were lost[9].Further studies identified twotandem duplications, DU1 and DU2,which were shown to vary across all of the BCG vaccine strains[10–12]. In addition to these major mutations, it has been demonstrated that single nucleotide polymorphisms (SNPs) might also play significant roles in the attenuation and variation of BCGs[13,14].

In our study, a strain labeled 3281, which was derived from an adult TB patient who reported having never been inoculated with a TB vaccine and was determined to be free of HIV infection, was screened and identified to be BCG. Our interest was aroused by the question how BCG turned into a pathogen despitebeing regarded as safe for years. Thepresent research compared a virulent BCG isolate withBCG vaccines.

## Results and Discussion

### Case finding

The strain 3281 was isolated from a 33 year old male, who lived in Hebei province, which is a none-animal-husbandry regionlocated in northern China. The patient worked in a commercial company which was not involved with livestock. The patient had never previously been diagnosed with tuberculosis and there was no known tuberculosis case among his family members or friends. The patient reported a cough and expectorate for less than 3 weeks before he consulted a doctor. The chest X-ray and CT demonstrated sign of pneumonia. Three consecutive sputa were all Acid-Fast Bacilli (AFB) positive while the *M*. *bovis BCG* strain was cultured from all of the sputa. Given these reason, we suggested that the *M*. *bovis BCG* strain might be the pathogen of this pneumonia patient.

This isolate 3281 belonged to a predominant spoligotype (SB0120) which was frequently reported both among human bovine TB and among cattle[15]. This spoligotype is similar to the spoligotype of the vaccine strain BCG type, and four strains out of the 14 *M*. *bovis* strains isolated from cattle in China during 2007 and 2008 had the same spoligotype[16].

### MIC(minimal inhibitory concentration)testing

*Mycobacterium tuberculosis s*usceptibility to 12 first- and second-line drugs were performed using Trek Sensitre MYCOTB MIC plate (MYCOTB; Trek Diagnostic Systemes, Cleveland, OH), with incubation at 37°C for 30 days. The MIC was recorded as the lowest antibiotic concentration that reduced visible growth (**Table 1**). The result showed that BCG 3281 showed a higher resistance to Ethionamide (5μg/ml) than BCG Pasteur (2.5μg/ml), *M*.*bovis*(1.2μg/ml) and H37Rv (0.6μg/ml). Meanwhile, BCG 3281 showed similar resistance to Isoniazid as *M*.*bovis* (0.12μg/ml), twice that of BCG Pasteur and H37Rv (0.06μg/ml). In addition, the resistance to Para-aminosalicylic acid, Kanamycin, Ofloxacin and Moxifloxacin of BCG 3281 was different with BCG Pasteur, indicating that BCG 3281 was not a traditional BCG strain.

**Table 1:** MIC testing results.

**Antibiotic**	**Concentration Range (μg/ml)**	**MIC (μg/ml)**			
		**BCG 3281**	**BCG Pasteur**	***M*. *bovis***	**H37Rv**
Cycloserine	2–256	16	16	16	8
Ethambutol	0.5–32	1	1	4	2
Ethionamide	0.3–40	**5**	2.5	1.2	0.6
Isoniazid	0.03–4	**0.12**	0.06	0.12	0.06
Para-aminosalicylic acid	0.5–64	0.5	1	64	0.5
Rifabutin	0.12–16	0.12	0.12	0.12	0.12
Rifampicin	0.12–16	0.12	0.12	0.5	0.25
Kanamycin	0.6–40	**1.2**	0.6	1.2	2.5
Ofloxacin	0.25–32	0.25	1	2	1
Moxifloxacin	0.06–8	0.06	0.25	0.5	0.5
streptomycin	0.25–32	0.25	0.25	1	0.5
Amikacin	0.12–16	0.12	0.12	0.5	0.5

### General genomic features

**Fig 1:**
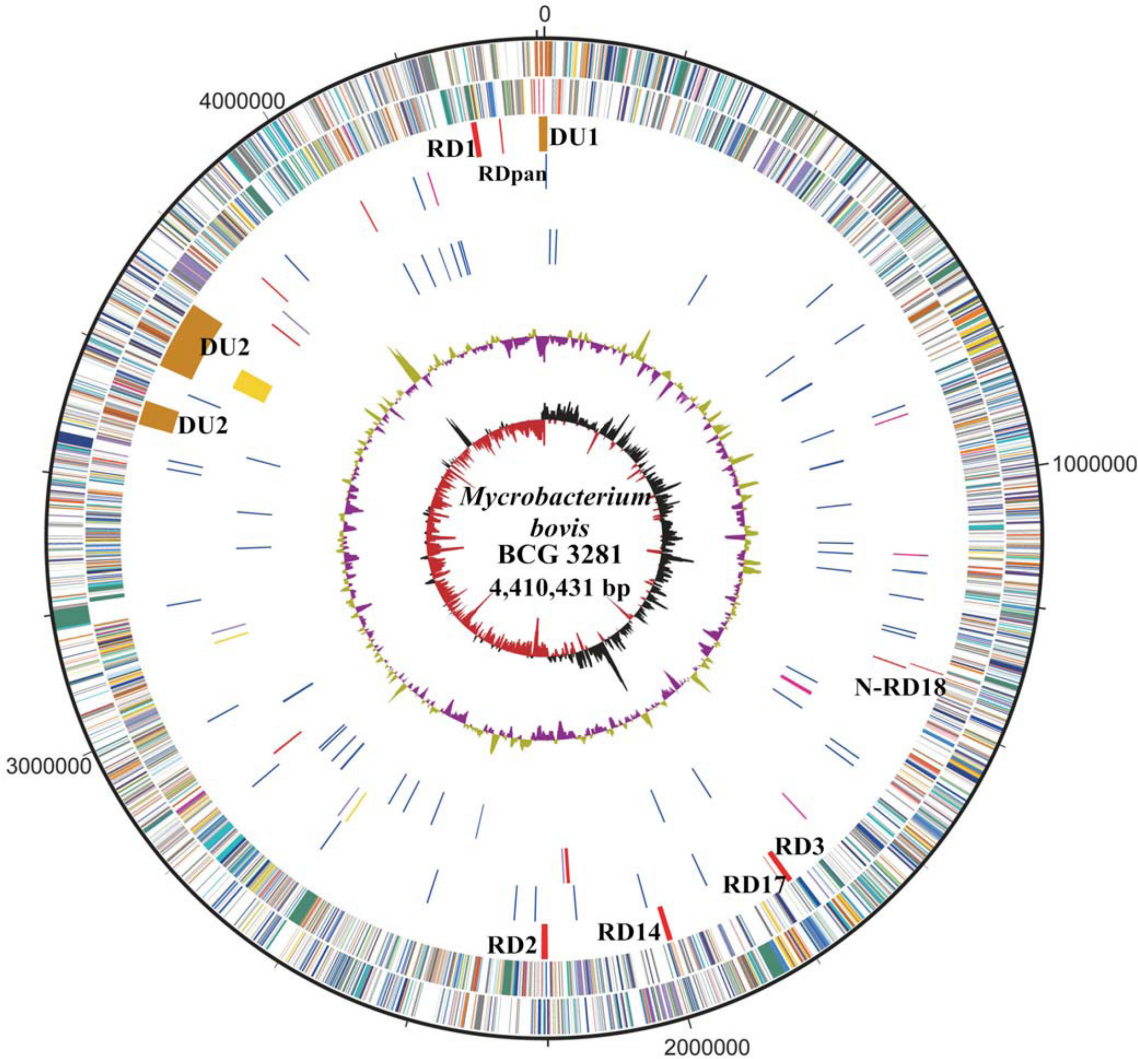
Circular representation of the *M*. *bovis* BCG 3281 chromosome. The outer black circle shows the coordinate. Moving inward, the next two circles show forward and reverse strand CDS, respectively, with colors representing the functional classification, the next circle shows RD(red) and DU (orange), followed by the 3281 unique SNP with nonsynonymous blue and synonymous red, then is the tRNA (blue) and rRNA (purple), final two are GC-content and GC-skew by using a 10-kb window.

The size of the BCG 3281genome was 4,410,431 bp (**Fig 1**), and the sequencing error was less than 1/Mb. Thus far, BCG 3281 has the largest genome size in terms of the genomes of BCG that have been completed. The genome of 3281 is 135,909 bps larger than that of BCG Pasteur (**Table 2**). A total of 4,186 CDSs were identified by glimmer-prediction and reference gene-alignment[17]. Among these CDSs, 3,079 might be COG categories with e-values 1e-5. No credible prophage was found, despite the finding that prophage genes produced four hits in the BCG 3281 genomevia phage-finder[18]. Due to the polymorphic G+C-rich sequences (PGRSs), most of which consist of enzymes involved in lipogenesis and lipolysisandthe Pro-Glu(PE) motif-Pro-Pro-Glu(PPE) motifgene family, BCG 3281’s GC-content was as much as 65.6%, which is similar to the GC contents of MTB and *M*. *bovis*[3]. Forty-fivetRNA operons were predicted by tRNAscan-SE,and one rRNAoperon was located by RNAmmer[19,20].

**Table 2:** Genome messages of strains used in this paper.

**Strain**	**Length(bp)**	**GC**	**CDSs**	**rRNA Operons**	**tRNA Operons**
*M*. *tuberculosis* H37Rv	4,411,708	65.62%	4111	1	45
*M*. *bovis* AF2122	4,345,492	65.63%	3918	1	45
*M*. *bovis* BCG Mexico	4,350,386	65.66%	3951	1	45
*M*. *bovis* BCG Tokyo	4,371,711	65.64%	3944	1	45
*M*. *bovis* BCG Pasteur	4,374,522	65.64%	3949	1	47
*M*. *bovis* BCG Korea	4,376,711	65.64%	4139	1	45
*M*. *bovis* BCG 3281	4,410,431	65.65%	4186	1	45

Genomic comparison with *M*.*bovis* and the four BCG strains revealed that the regions of difference (RDs)that contain virulence genes that were lost in the BCGswere also absent in 3281. Compared to the other BCGs and *M*. *bovis*, 35 BCG 3281-specific single nucleotide polymorphisms (SNPs) were identified (**Fig 1**), and 23of these SNPs produced nonsynonymous variations. Additionally, nineindels (threeinsertions and sixdeletions) were found to be exclusive to BCG 3281, and fourother deletions were shared only with *M*. *bovis* only. A total of 20 genes were affected by the 23 nonsynonymous variations (**S1 Table**), and 50 genes were affected by the 13 indels (**Table 3 and** S2 Table).

**Table 3:** CDSs involved in indels between *M*. *bovis* and BCGs.

**CDSs**	**Function**	**CDSs**	**Function**
GS11_3486	TetR family transcriptional regulator	Mb3236c	hypothetical protein
GS11_3501	acetyl- CoA carboxylase biotin carboxyl carrier protein subunit	Mb3237	ATP-dependent RNA helicase RhlE
GS11_3519	hypothetical protein Mb3266c	Mb3238	hypothetical protein
BCG_1955c	PPE family protein	Mb3239c	SOJ/PARA-like protein
BCG_2407	hypothetical protein	Mb3240	acid phosphatase
BCG_3228	hypothetical protein	Mb3241	isochorismate synthase
BCG_3354c	L-lysine-epsilon aminotransferase lat'	Mb3242	acetyltransferase
Mb1951c	malto-oligosyltrehalose synthase	Mb3243c	hypothetical protein
Mb2572	lipoprotein LppA	Mb3244	hypothetical protein
Mb2573	lipoprotein LprR	Mb3245	transcriptional regulator WhiB
Mb3220	ABC transporter ATP-binding protein	Mb3246c	two component sensor kinase
Mb3222c	DNA helicase II	Mb3247c	acetyl- CoA carboxylase biotin carboxyl carrier protein subunit
Mb3223	glutaredoxin protein	Mb3248c	anti-sigma factor
Mb3224c	NADH pyrophosphatase	Mb3249c	hypothetical protein
Mb3225c	transmembrane cation transporter	Mb3250c	RNA polymerase sigma factor RpoE
Mb3226c	ATP-dependent DNA helicase	Mb3251	short chain dehydrogenase
Mb3227c	ATP-dependent DNA helicase	Mb3254c	hypothetical protein
Mb3228	lipase LipV	Mb3255c	hypothetical protein
Mb3229	DNA-methyltransferase	Mb3256	transferase
Mb3230c	hypothetical protein	Mb3257	hypothetical protein
Mb3231c	molybdopterin biosynthesis-like protein MoeZ	Mb3258c	3-phosphoshikimate 1-carboxyvinyltransferase
Mb3232c	hypothetical protein	Mb3259c	hypothetical protein
Mb3233	TetR family transcriptional regulator	Mb3319c	AsnC family transcriptional regulator
Mb3234c	hypothetical protein	Mb3320	hypothetical protein
Mb3235	hypothetical protein	Mb3321	piperideine-6-carboxilic acid dehydrogenase

The GS11 is the official locus of BCG 3281 given by Genbank.

### Unique genomic features of the BCG strains

Thirteen years of laboratory cultivation have caused great differences in virulence between the progeny and the original strainand resulted in the attenuated virulence and sufficient reserved antigenicity for protection against TB. Comparative genomic analyses have revealed massive discrepancies between BCG and *M*. *bovis*. The most significant two events were the loss of the RD1 regions that contain a specialized secretion system that is strongly associated with pathogenic ability[11,21]and the two tandem duplications, DU1 and DU2. DU1 is restricted to BCG Pasteur 1173P2, and DU2 is present in four different types in different BCGs[10,22].

**Fig 2:**
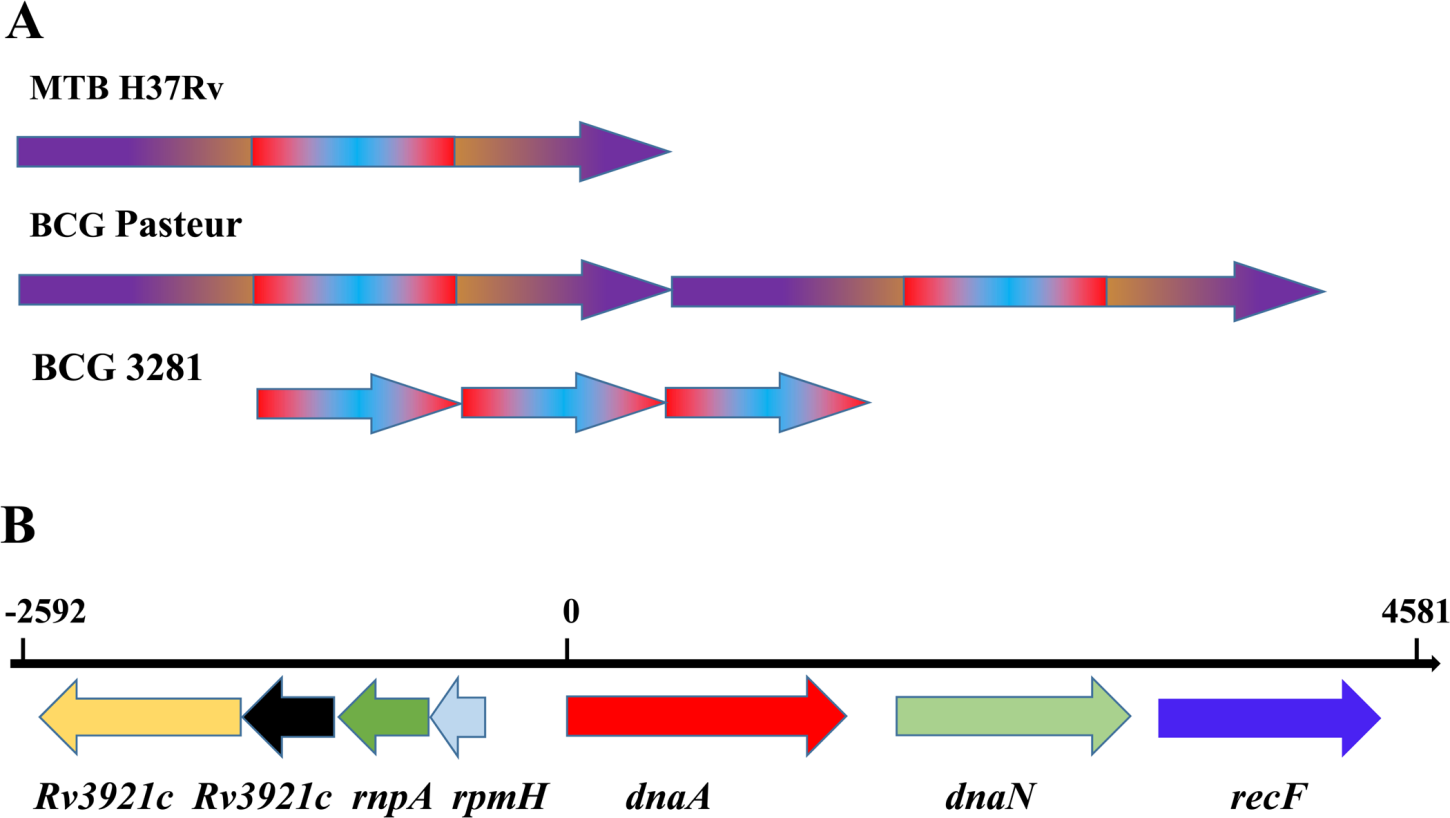
Scheme showing the DU1 region of BCG 3281 and BCG Pasteur 1173p2. (A). The color schemes means duplicated regions. (B). Details of genes involved in the BCG 3281 duplicated units (using H37Rv coordinate).

In the genome sequence of BCG 3281, a loss of RD1and duplications in the DU1 and DU2 regions were observed, which validates this stain as BCG. In the DU1 region, a 7 kb unit that covered six genes and crossed the *ori*C was found to be repeated three times (**Fig 2**); this duplication is specific to BCG 3281 and has never been reported before (**Table 4 and 5**). The DU1 in BCG Pasteur is 29.7 Kb, encompassing the region from *Rv3910* to *Rv0013*, while the DU1 in BCG 3281 is only 7.2 Kb, including the region from *Rv3921c* to *Rv0003*. BCG 3281 has three copies of *dnaA*-*dnaN* region with functional oriC.Protein DnaA initiates chromosome replication when accumulated to the ‘initiation’ level[23], and multiple copies of *dnaA* in BCG 3281 might help the strain increase growth rate [24]and activate some gene expression [25].Thusthe triploidfor DNA replication elements might partly contribute to the pathogenic of BCG 3281.

**Table 4:** Summary of DU1 regions within *M*. *bovis* BCG Pasteur, Mexico, Tokyo, Korea and 3281.

**Strain**	**H37Rv Coordinate**	**Unit Length**	**Repeat Times**	**Total Length**
BCG Pasteur	4398772..16733	29.7kb	2	59.4kb
BCG Mexico	NA			
BCG Tokyo	NA			
BCG Korea	NA			
BCG 3281	4409117..4581	7.2kb	3	21.6kb

“NA” means not present.

**Table 5:** Genes in the duplication unit that located at DU1 region of *M*. *bovis* BCG 3281.

**Genes**	**Length**	**Function**
GS11_4181	507	dnaA, chromosomal replication initiation protein
GS11_4182	402	dnaN, DNA polymerase III subunit beta
GS11_4183	385	recF, recombination protein F
GS11_4184	366	oxaA, inner membrane protein translocase component YidC
GS11_4185	62	rnpA, ribonuclease P protein component
GS11_4186	47	rpmH, 50S ribosomal protein L34

The DU2 zone of BCG 3281 belongs to the DU2-Ⅳ type, which consists of two repeat units (41 kb and 37.5 kb) that correspondto regions 3,567,459–3,608,472 and 3,671,536–3,709,097 of *M*. *tuberculosis*H37Rv that are separate and repeattwice (**Table 6**).

**Table 6:** Summary of DU2 reagions with in *M*. *bovis* BCG Pasteur, Mexico, Tokyo, Korea and 3281.

**Strain**	**Type**	**H37Rv Coordinate**	**Unit Length**	**Repeat Times**	**Total Length**
BCG Pasteur	DU2-Ⅳ	3590899..3608474	17.5kb	2	72kb
		3671533..3690125	18.5kb	2	
BCG Mexico	DU2-Ⅳ	3590899..3608474	17.5kb	2	72kb
		3671533..3690125	18.5kb	2	
BCG Tokyo	DU2-Ⅰ	3684226..3705104	20.8kb	3	62.4kb
BCG Korea	DU2-Ⅳ	3590899..3608473	17.5kb	3	106.5kb
		3671533..3690125	18kb	3	
BCG 3281	DU2-Ⅲ	3567459..3608472	41kb	2	157kb
		3671536..3709097	37.5kb	2	

The loss of RD1 and the two identified tandem duplications in BCG 3281 confirmed that the strain is a BCG. This result is completely contrasted with our expectation that BCG 3281 would be an *M*. *bovis*. Furthermore, the RD17 and RDpan, which are specific to BCGsand lost in *M*.*bovis*AF2122, were also found in BCG 3281[26].

**Fig 3:**
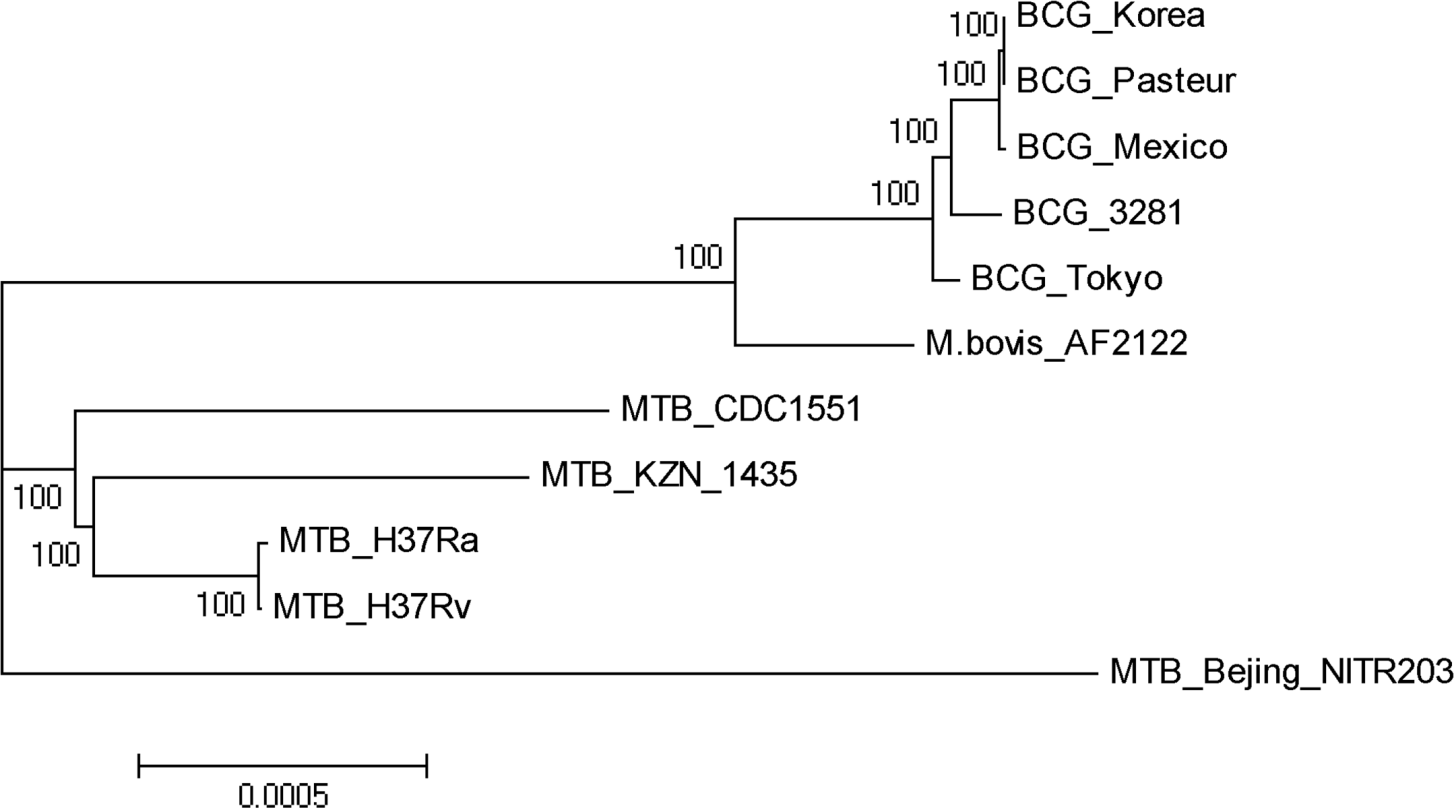
Phylogenetic tree of *M*. *tuberculosis*, *M*. *Bovis* and BCGS. The tree was constructed employing Neighbor-joining method. It is based on the SNPs within 2263 core genes of the strains.

To ensure the accuracy of the strain identification, a SNP-based NJ phylogenetic tree was constructed (**Fig 3**). The phylogenetic position of BCG 3281was located near BCG Tokyo and far from the clinic strains, which validated 3281 as a BCG. For years, people have acknowledged that BCG strains are safe for vaccination and have notransmissibility. Nevertheless, thestrain 3281, which was isolated from an adult patient who had not been vaccinated with a BCG, was identified to be a BCG. We believe that the source of pathogen in this case was from the vaccine and had mutated to acquire the ability for horizontal transmission.

### Antigen epitopevariations

Epitopes are the parts of antigens that are recognized by T-cell receptors (TCRs) and B-cell receptor (BCRs) and play the core role in the immune response. We believed similarities between the epitopes of BCG 3281 and *M*. *Bovis* or MTB would exist because all of these strains are pathogenic.

To identify the variations in the epitopes of these strains, 2,667 epitopes complied from the Immune Epitope Database(IEDB)[27], including 2,055 T-cell epitopes and 612 B-cell epitopes, were selected and renamed (**S3 Table**).These epitopes were subsequently positively experimentally identified by IEDB. Four complete genome BCG vaccines (i.e., BCG Pasteur 1173P2, BCG Tokyo 172, BCG Mexico and BCG Korea 1168P) were acquired from the National Center of Biotechnology Information (NCBI).

Only 100% identical match results were considered as the same epitopes because recent studies have shown that human T cell epitopes of *Mycobacteriumtuberculosis* are evolutionarily hyper-conserved[28]. For comparison, 1,600 epitopes, including 1,213 T-cell epitopes and 387 B-cell epitopes, were identified in all seven strains (BCG 3281, BCG Pasteur 1173P2, BCG Tokyo 172, BCG Mexico, BCG Korea1168P, *M*.*bovis*AF2122 and *M*. *tuberculosis* H37Rv). In contrast, 531 epitopes, including 404 T-cell epitopes and 127 B-cell epitopes, were absent in all seven strains. Moreover, 329 epitopes, including 290 T-cell epitopes and 39 B-cell epitopes, were found to be lost in only BCG 3281 and other BCGs. Additionally, 44 epitopes, including 33 T-cell epitopes and 11 B-cell epitopes, located in 22 geneswere found to be missing in only BCG 3281. When these 22 genes were examined, frameshiftswere found to have occurred in the coding regions of 19genes and 3 genes were lost (**S4 Table**).

**Fig 4:**
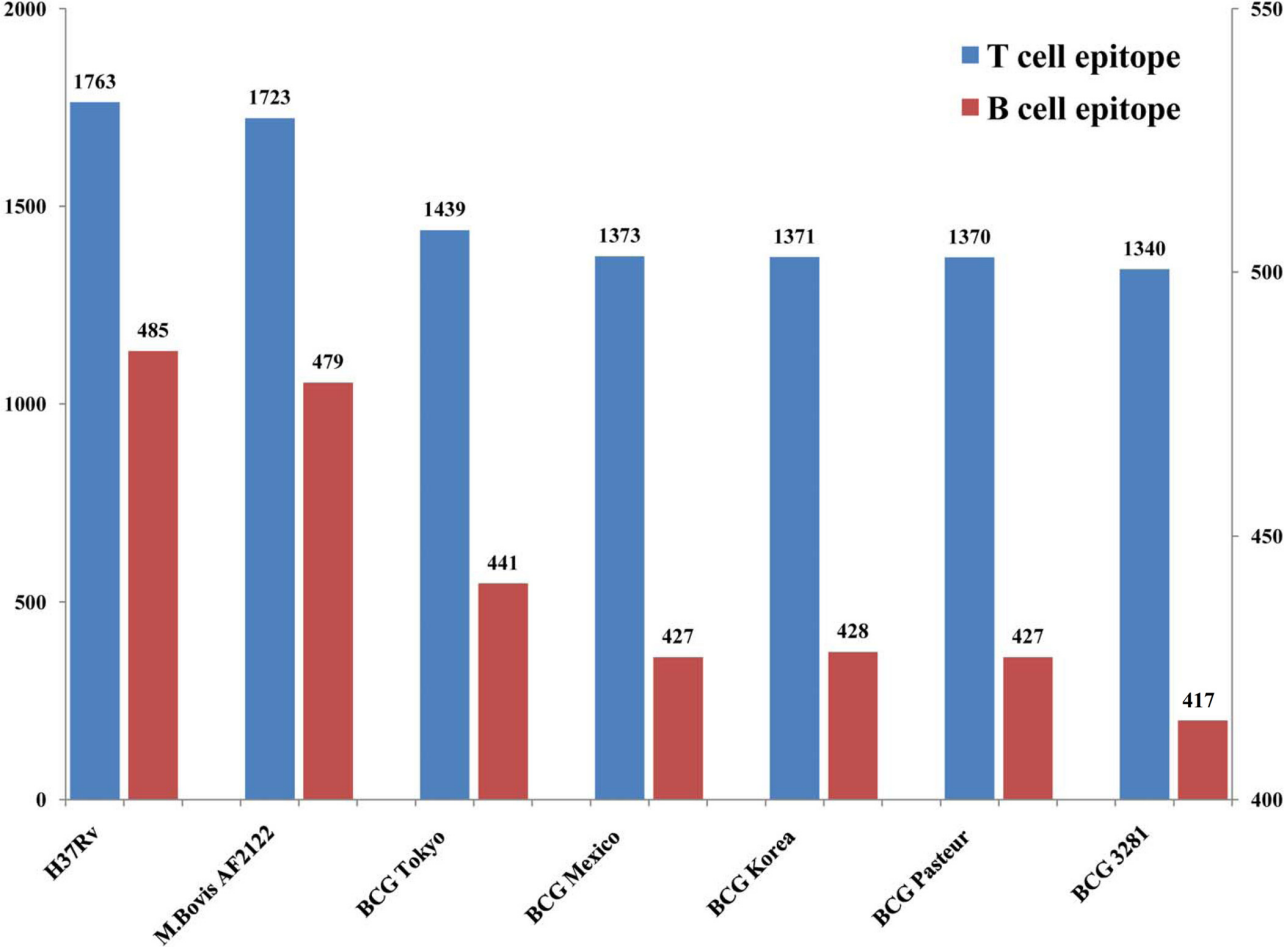
Epitopes in *M*. *tuberculosis* H37Rv, *M*. *bovis* AF2122 and genome finished BCGs. Duplicate epitopes were removed and only epitopes with 100% identical matches were considered present in the strain.

Despite sharing majorities of both T-cell and B-cell epitopes with H37Rv and *M*. *bovis*, the BCGs obviously possess fewer epitopes (**Fig 4**), whichmight result in reduced protection againstTB. In other words, fewer epitopes indicate poorer recognition of alien invadersby the human body. Moreover, BCG 3281 had the fewest number of epitopes among the BCGs, which amplifies the possibility for immune escape. Wen et al. found that BCG Tokyo possess the greatest number of both T-cell and B-cell epitopes among the BCGs and thus might be the vaccine that confers the best immune protection[29]. We found that 62 unique epitopes of BCG Tokyothat are locatedin two BCG Tokyo genes,*JTY1991* and *JTY1996*, that were also present in *M*.*bovis* and H37Rvbut absent in other BCGs. The efficiency of BCG protection might be improved by the transduction of two genes into other BCG vaccines. No epitopes unique to 3281 among the other BCGs were identified. In one aspect, this might hint that BCG 3281 did not obtain exogenous genetic element through lateral gene transfer, emphasizing the possibility that pathogenic BCG 3281 might be formed through mutation from BCG vaccine. On the other hand, epitopes that had not been experimentally identified might existed in BCG 3281 unique genes.

### Virulence factors in BCG 3281

#### Variation in known virulence factors

BecauseBCG 3281 was considered to be a pathogenic bacterium, we expected that BCG 3281would share extensive similarities with MTB and *M*. *bovis*and possess distinct genetic differences from other BCGs, particularly with respect to virulence genes. To detect the variationsin the virulence factors, 88 virulence genes that were identified from the Virulence Factors Database (VFDB) were selected[30]. Blastpresults (**Table 7**) revealed that 51 virulence genes were 100% identical with *M*. *bovis* and the five CG strains,threegenes (located at RD5) were lost in both *M*. *bovis* and all of the BCGs, and sevengenes were *M*. *bovis-*specific; the latter genes were located at RD1 and were lost in all of the BCGs. A copy number variation (CNV) of one gene (*VFG1412*) was found and was located in the DU2 region. Additionally, a frameshiftin one virulence gene (*VFG2388*) was found in both *M*. *bovis* and the BCGs. Moreover, plentiful nonsynonymousmutations were identified. To our surprise, no virulence genes were found to be specific to BCG 3281 with respect to *M*. *bovis* and the other BCGs. Although the differences between *M*. *bovis*and BCG 3281were enormous, these differences were found to be common characteristics of BCGs.

**Table 7:** Comparison of mutative virulence factors within *M*. *bovis* AF2122 and *M*. *bovis* BCG 3281, Pasteur, Tokyo, Mexico and Korea.

**Virulence Factors**	***M. bovis***	**BCG 3281**	**BCG Pasteur**	**BCG Tokyo**	**BCG Mexico**	**BCG Korea**
*VFG1382*	+	+	+	1	+	+
*VFG1384*	1	1	1	1	1	1
*VFG1385*	1	1	1	1	1	1
*VFG1386*	+	1	+	+	+	+
*VFG1390*	1	1	1	1	1	1
*VFG1391*	2	2	2	2	2	2
*VFG1396*	1	1	1	1	1	1
*VFG1400*	-	-	-	-	-	-
*VFG1401*	-	-	-	-	-	-
*VFG1402*	-	-	-	-	-	-
*VFG1407*	1	1	1	1	1	1
*VFG1408*	1	1	1	1	1	1
*VFG1409*	1	1	1	1	1	1
*VFG1412*	1 copy	2 copies	2 copies	1 copy	2 copies	3 copies
*VFG1421*	1	1	1	1	1	1
*VFG1422*	+	-	-	-	-	-
*VFG1423*	+	-	-	-	-	-
*VFG1812*	+	1	1	1	1	1
*VFG1815*	+	+	1	+	+	1
*VFG1816*	1	1	1	1	1	1
*VFG1818*	1	1	1	1	1	1
*VFG1820*	2	2	2	2	2	2
*VFG1825*	1	1	1	1	1	1
*VFG2378*	+	-	-	-	-	-
*VFG2379*	+	-	-	-	-	-
*VFG2380*	1	1	1	1	1	1
*VFG2383*	+	trancated	trancated	trancated	trancated	trancated
*VFG2384*	+	-	-	-	-	-
*VFG2385*	+	-	-	-	-	-
*VFG2387*	2	2	2	2	2	2
*VFG2388*	frameshift	frameshift	frameshift	frameshift	frameshift	frameshift
*VFG2389*	trancated	-	-	-	-	-
*VFG2391*	1	1	1	1	1	1
*VFG2394*	3	3	3	3	3	3
*VFG2395*	1	2	2	2	2	2
*VFG2397*	1	1	1	1	1	1
*VFG2398*	2	2	1	1	1	1

“+” means 100% in identit, “-” stands for lost, the number shows nonsynonymous mutations number or copy number.

#### Possible virulence genes

**Fig 5:**
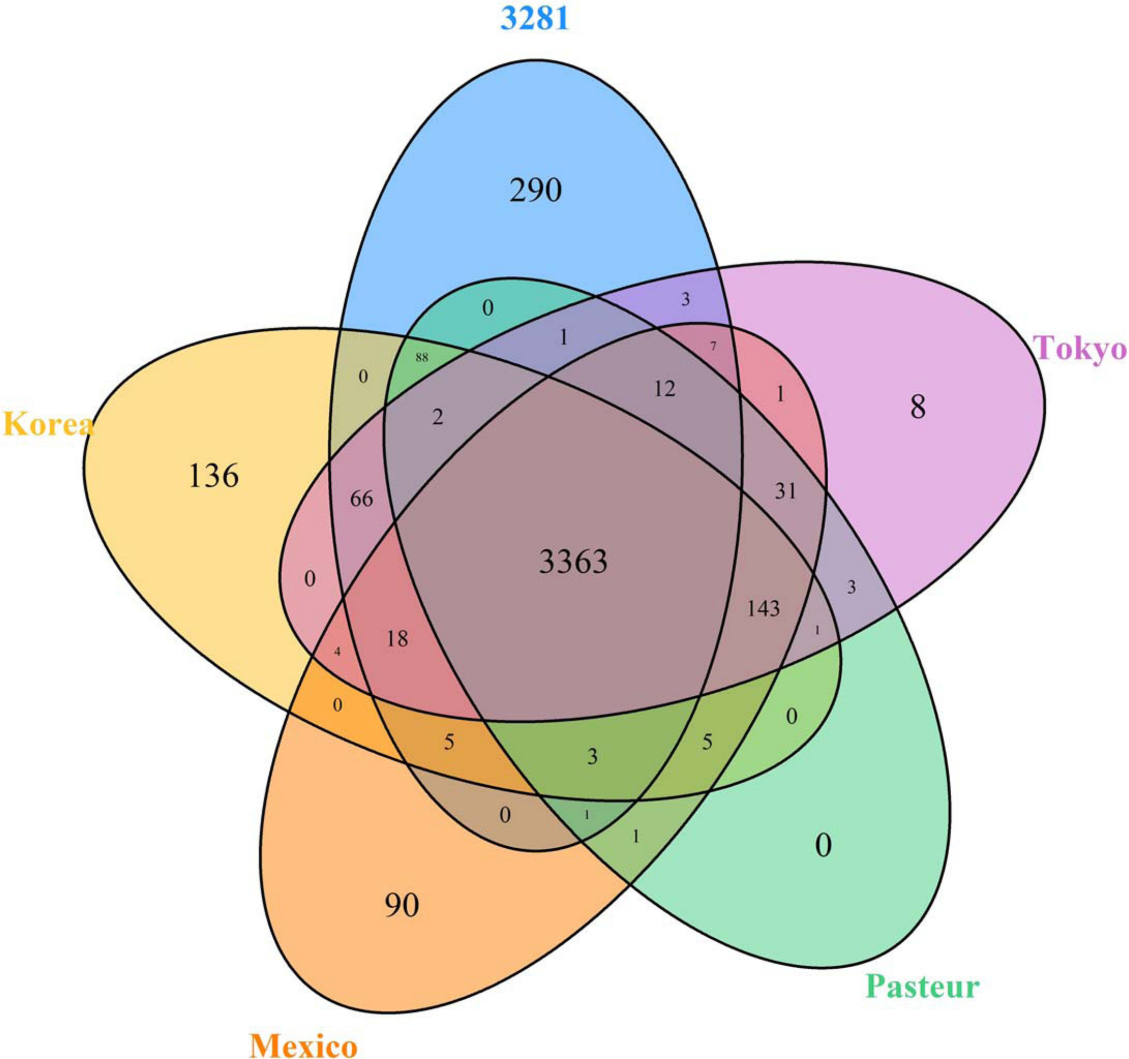
The Venn diagram showing core orthologs of five genome finished BCGs. Genes overlapping at least 50% length and 50% (by PGAP) of similarity were considered orthologs.

Because no large variations in confirmed virulence genes were detected within BCG 3281, a pan-genome analysis was performed to identify possiblenew virulence factors[31]. Via the use of the pan-genome analysis pipeline (PGAP),orthologous clusters within the 5 BCGs were grouped (**Fig 5**)[32]. The pan-genome clusters consisted of 4,282 orthologs and had a core of 3,363 orthologs.Two hundred and ninety orthologclusters contained 294 CDSs that were likely to be unique to 3281 and might have conferred additional virulence to BCG 3281.

Considering the prediction discrepancyand the restrictions of the software, we searched these 294 CDSs within the genome and re-predicted the CDS libraries of the other fourBCGs. Ultimately, fourCDSs were proven to be 3281-specific, and all of these CDSs were generated by indels (**Table 8**).

**Table 8:** CDSs inferred with potential virulence in *M. bovis* BCG 3281.

**CDS**	**Length**	**Variation**	**Former Function**
GS11_0276	1056	a single nucleotide deletion	succinate dehydrogenase flavoprotein subunit
GS11_0578	1737	9 nucleotides deletion	PE-PGRS family protein
GS11_1865	2145	a single nucleotide insertion	WAG22 antigen
GS11_3751	5442	9 nucleotides insertion	PE-PGRS family protein

The GS11 is the official locus of BCG3281 given by Genbank.

## Conclusions

Although BCG, which is an attenuated derivativeof *M*. *bovis*, has served for nearly 90 years as the sole vaccine that provides protection against tuberculosis, the clinical strain 3281 was proven to be a BCG and was found to be morbigenous. In an effort to determine the genetic structure of BCG 3281 and determine whether a BCG could be pathogenic, we sequenced the complete genome of BCG 3281 and compared its entire genome to four complete BCG genomesand the genome of *M*. *bovis* AF2122. First, to demonstrate the accuracy of the physiological and biochemical identification results, we examined the tandem duplicationsDU1 and DU2, which are significant characteristics of BCGs. Simultaneously, a genetic evolution analysis of the complete BCG genomes and the genome of *M*. *bovis* was constructed. The results of both analyses verified that strain 3281 is a BCG.

Examinations of all of the BCG genomes, includingthose of BCG Pasteur, Tokyo, Mexico, Korea,Frappier, Glaxo, Moreau, Phipps, Prague, Sweden, China, ATCC35733, ATCC35740 and ATCC35743,revealed that none contained the 7 kb duplication in the DU1 region.The presence of the *dnaA and dnaN*genes is strongly associated with the initiation and regulation of chromosomal replication; thus, we inferred that BCG 3281would likely be capable of enduring greater burdens in replication[24].

To determine whether any identified virulence factors were unique to 3281 relative to the other BCGs, 88 virulence genes located at H37Rv were examined; 3281-unique indels anda single amino acid polymorphismwere located, but 3281-unique virulence factors were not found. We believe that these variations might influence the virulence of BCG 3281 to some extent but not so much as to convert an attenuated vaccine into a pathogenic bacterium. To identify the possible virulence factors, a pan-genome method was applied and four BCG 3281-unique CDSs were identified as puativevirulence genes since no other large variations in genome structure were found.Additionally, we detected antigen epitope variationsin BCG 3281. Compared to the other BCGs, BCG 3281 has lost more epitopes, which might intensify this strain’s potential for immune escape and increase the risk of secondary infection.Overall, this study provides initial insight into the characteristics of a pathogenic BCGthat should have significant effects on TB vaccine research.

## Materials and Methods

### Strain Information

The mycobacterial strain used in this study was acquired from the Beijing Bio-Bankof clinical resourceson Tuberculosis (D09050704640000)". This strain was originally isolated from an adult male patientwho was not infected with HIV.

### Genome sequencing, assembly and annotation

Through a combination of next-generation sequencing (NGS) techniques, thegenome was sequenced with both a 454 GS-FLX system and a Hiseq2500. The 454 data were assembled with Newbler 2.5 withcoverage of 29.6.Using Soap 1.05, the Hiseq reads were assembled with a 108.9-fold coverage[33]. Gap closure was performed using the PCR method with the help of ContigScapeusing the 454 assembly results[34]. The low value dots were verified by the Hiseq assembly results. ORFs were predicted with Glimmer 3.0.2 and replenished by reference annotation[35].

### SNP and Phylogenetic analyse

All SNPs were identified with Mauve 2.3.1, and they were localized to CDSsvia an in-house Perl script[36].The pangenomemethod was employed for the phylogenetic analysis. A core of 2,263 geneslengths of at least 0.8 and similarities of at least 0.8 was generated. The neighbor-joining tree was generated by MEGA with a bootstrap value of 1,000[37].

## Supporting Information

S1 Table Details of *M*. *bovis*BCG 3281 specific SNPs. (DOC)

S2 Table Details of indels between *M*. *bovis* and genome finished BCGs. (DOC)

S3 Table . Detailed information of epitopes used in this paper. (DOC)

S4 Table . Details of the lost epitopes by BCG 3281 alone. (DOC)
