# Correction: Genomic Analysis of a Mycobacterium Bovis Bacillus Calmette-Guérin Strain Isolated from an Adult Patient with Pulmonary Tuberculosis

**DOI:** 10.1371/journal.pone.0129753

**Published:** 2015-05-29

**Authors:** The PLOS ONE Staff

## Notice of Republication

This article was republished on May 14, 2015, to correct an error in the title: “Bovis Bacillus” was incorrectly displayed as one word. Please download this article again to view the correct version. The originally published, uncorrected article and the republished, corrected article are provided here for reference.

## Supporting Information

S1 File Originally published, uncorrected article. (PDF)

S2 File Republished, corrected article. (PDF)
