# Supplementary material for: Genomic Analysis of a Mycobacterium Bovis Bacillus Calmette-Guérin Strain Isolated from an Adult Patient with Pulmonary Tuberculosis
Source: PLoS One. 2015 Apr 13;10(4):e0122403. doi: 10.1371/journal.pone.0122403 (PMC4395146; doi:10.1371/journal.pone.0122403)
Supplement: S1 Table — (DOC) [file pone.0122403.s001.doc]

| **Table S1 Details of *M. bovis* BCG 3281 specific SNPs.** | | | | | | | | | | | | | |
| --- | --- | --- | --- | --- | --- | --- | --- | --- | --- | --- | --- | --- | --- |
| **SNP pattern** | **M. bovis AF222** | **3281** | **Pasteur** | **Tokyo** | **Korea** | **Maxico** | **Mbovis Gene** | **Gene start** | **Gene End** | **SNP location** | **M.bovis Codon** | **3281 codon** | **SNP Type** |
| AGAAAA | 1517 | 1517 | 31184 | 1517 | 1517 | 1517 | **Mb0001** | 1 | 1524 | 1517 | AAG->K | AGG->R | non-synonymous |
| CTCCCC | 594521 | 594518 | 624168 | 594520 | 594501 | 594502 | **Mb0514** | 593942 | 595018 | 580 | CGC->R | TGC->C | non-synonymous |
| CACCCC | 688043 | 688023 | 717682 | 688043 | 688015 | 688016 | **Mb0603** | 687171 | 688058 | 873 | TTC->F | TTA->L | non-synonymous |
| CTCCCC | 854161 | 854099 | 883754 | 855472 | 854087 | 854033 | **Mb0780** | 853798 | 854541 | 364 | CCC->P | TCC->S | non-synonymous |
| GCGGGG | 1134228 | 1134612 | 1164266 | 1135927 | 1134599 | 1134545 | **Mb1042c** | 1134360 | 1133785 | 133 | CGT->R | GGT->G | non-synonymous |
| TCTTTT | 1134230 | 1134614 | 1164268 | 1135929 | 1134601 | 1134547 | **Mb1042c** | 1134360 | 1133785 | 131 | AAG->K | AGG->R | non-synonymous |
| GCGGGG | 1214616 | 1214901 | 1244555 | 1215893 | 1214888 | 1214834 | **Mb1116** | 1212920 | 1215244 | 1697 | GGC->G | GCC->A | non-synonymous |
| CTCCCC | 1286596 | 1286914 | 1316568 | 1287906 | 1286901 | 1286847 | **Mb1190** | 1286364 | 1287659 | 233 | GCA->A | GTA->V | non-synonymous |
| CGCCCC | 1547231 | 1547610 | 1575712 | 1548598 | 1546045 | 1545991 | **Mb1411** | 1545827 | 1547320 | 1405 | CAA->Q | GAA->E | non-synonymous |
| GAGGGG | 1906330 | 1898106 | 1926206 | 1899108 | 1896433 | 1896485 | **Mb1722** | 1905025 | 1906788 | 1306 | GAT->D | AAT->N | non-synonymous |
| AGAAAA | 2023469 | 2015006 | 2034016 | 2016007 | 2004171 | 2013381 | **Mb1823** | 2022695 | 2024206 | 775 | ATC->I | GTC->V | non-synonymous |
| CTCCCC | 2154127 | 2145521 | 2164946 | 2146937 | 2135101 | 2144311 | **Mb1951c** | 2158945 | 2153213 | 4819 | GAT->D | AAT->N | non-synonymous |
| GAGGGG | 2279885 | 2259767 | 2279624 | 2272402 | 2249269 | 2258989 | **Mb2074c** | 2290899 | 2278444 | 11015 | CCG->P | CTG->L | non-synonymous |
| CTCCCC | 2664305 | 2641444 | 2661310 | 2654082 | 2630955 | 2640675 | **Mb2421c** | 2664716 | 2663865 | 412 | GGG->G | AGG->R | non-synonymous |
| GAGGGG | 2834683 | 2811162 | 2831682 | 2824455 | 2801330 | 2811047 | **Mb2574** | 2834577 | 2835239 | 107 | GGC->G | GAC->D | non-synonymous |
| TCTTTT | 2979233 | 2955763 | 2976282 | 2969055 | 2945930 | 2955647 | **Mb2729** | 2979097 | 2980068 | 137 | GTC->V | GCC->A | non-synonymous |
| ACAAAA | 3204881 | 3181780 | 3202297 | 3195124 | 3171945 | 3181662 | **Mb2956** | 3202018 | 3207648 | 2864 | CAC->H | CCC->P | non-synonymous |
| CACCCC | 3455765 | 3433009 | 3453703 | 3446353 | 3422998 | 3433068 | **Mb3159** | 3455106 | 3456251 | 660 | AAC->N | AAA->K | non-synonymous |
| CGCCCC | 3471281 | 3448525 | 3469219 | 3461869 | 3438514 | 3448584 | **Mb3174** | 3470521 | 3471858 | 761 | TCG->S | TGG->W | non-synonymous |
| GAGGGG | 3596610 | 3573799 | 3594493 | 3587144 | 3563788 | 3573858 | **Mb3290** | 3596135 | 3597481 | 476 | GGC->G | GAC->D | non-synonymous |
| GCGGGG | 3832427 | 3887852 | 3866298 | 3864136 | 3871703 | 3845663 | **Mb3496** | 3831660 | 3832613 | 768 | GAG->E | GAC->D | non-synonymous |
| GAGGGG | 4000912 | 4058259 | 4036696 | 4033947 | 4038942 | 4016061 | **Mb3648c** | 4001141 | 4000491 | 230 | ACC->T | ATC->I | non-synonymous |
| AGAAAA | 4106533 | 4164205 | 4142641 | 4139834 | 4144830 | 4122006 | **Mb3750** | 4105996 | 4106760 | 538 | ACG->T | GCG->A | non-synonymous |
| GAGGGG | 1277777 | 1278167 | 1307821 | 1279159 | 1278154 | 1278100 | **Mb1179c** | 1279120 | 1277672 | 1344 | ACC->T | ACT->T | synonymous |
| CTCCCC | 1277804 | 1278194 | 1307848 | 1279186 | 1278181 | 1278127 | **Mb1179c** | 1279120 | 1277672 | 1317 | AAG->K | AAA->K | synonymous |
| CTCCCC | 1530670 | 1531060 | 1559149 | 1532036 | 1529482 | 1529428 | **Mb1396c** | 1531668 | 1530478 | 999 | GCG->A | GCA->A | synonymous |
| CGCCCC | 2241614 | 2221493 | 2241350 | 2234128 | 2210995 | 2220715 | **Mb2038c** | 2241988 | 2240732 | 375 | GGG->G | GGC->G | synonymous |
| GAGGGG | 2442731 | 2422363 | 2442228 | 2435000 | 2411873 | 2421593 | **Mb2222c** | 2442799 | 2442380 | 69 | GGC->G | GGT->G | synonymous |
| CTCCCC | 2834684 | 2811163 | 2831683 | 2824456 | 2801331 | 2811048 | **Mb2574** | 2834577 | 2835239 | 108 | GGC->G | GGT->G | synonymous |
| CTCCCC | 2834693 | 2811172 | 2831692 | 2824465 | 2801340 | 2811057 | **Mb2574** | 2834577 | 2835239 | 117 | CCC->P | CCT->P | synonymous |
| GCGGGG | 3832430 | 3887855 | 3866301 | 3864139 | 3871706 | 3845666 | **Mb3496** | 3831660 | 3832613 | 771 | CTG->L | CTC->L | synonymous |
