# Supplementary material for: Genomic Analysis of a Mycobacterium Bovis Bacillus Calmette-Guérin Strain Isolated from an Adult Patient with Pulmonary Tuberculosis
Source: PLoS One. 2015 Apr 13;10(4):e0122403. doi: 10.1371/journal.pone.0122403 (PMC4395146; doi:10.1371/journal.pone.0122403)
Supplement: S2 Table — (DOC) [file pone.0122403.s002.doc]

| **Table S2 Details of indels between *M. bovis* and BCGs.** | | |
| --- | --- | --- |
| **M. bovis and 3281 specific deletions**(BCG Pasteur coordinate) | | |
| **length** | **start** | **end** |
| 57 | 2164584 | 2164640 |
| 46 | 3109919 | 3109964 |
| 26 | 2652215 | 2652240 |
| 7 | 3806768 | 3806774 |
| **3281 specific deletions**(*Mycobacterium Bovis* coordinate) | | |
| **length** | **start** | **end** |
| 128 | 2153694 | 2153821 |
| 217 | 2153822 | 2154038 |
| 72 | 2157270 | 2157341 |
| 657 | 2833879 | 2834535 |
| 41 | 3772798 | 3772838 |
| 70 | 3772901 | 3772970 |
| **3281 specific insertions**(BCG 3281 coordinate) | | |
| **length** | **start** | **end** |
| 42441 | 3621809 | 3664249 |
| 46 | 3089402 | 3089447 |
| 26 | 2632350 | 2632375 |
