# Supplementary material for: Genomic Analysis of a Mycobacterium Bovis Bacillus Calmette-Guérin Strain Isolated from an Adult Patient with Pulmonary Tuberculosis
Source: PLoS One. 2015 Apr 13;10(4):e0122403. doi: 10.1371/journal.pone.0122403 (PMC4395146; doi:10.1371/journal.pone.0122403)
Supplement: S3 Table — (DOC) [file pone.0122403.s003.doc]

| **Table S3 Detailed information of epitopes used in this paper.** | | |
| --- | --- | --- |
| **Name** | **Length** | **Epitope Sequence** |
| TcellP0001 | 20 | AAAAKLAGLVFPQPPAPIAV |
| TcellP0002 | 20 | AAAAVAHAVAPAGVGGTALT |
| TcellP0003 | 20 | AAAGFASKTPANQAISMIDG |
| TcellP0004 | 20 | AAAGTAVQDSRSHVYAHQAQ |
| TcellP0005 | 15 | AAAQASAAAAAYEAA |
| TcellP0006 | 8 | AAASAIQG |
| TcellP0007 | 16 | AAASAIQGNVTSIHSL |
| TcellP0008 | 15 | AAASWDALAAELASA |
| TcellP0009 | 18 | AADELVGGPPVEASAAAL |
| TcellP0010 | 20 | AADLLAAAAAWNGIAVEVST |
| TcellP0011 | 15 | AADMWGPSSDPAWER |
| TcellP0012 | 20 | AADTGLEAVRQFLNLDVPLF |
| TcellP0013 | 20 | AAEQAAARRRRIVDHLAHAG |
| TcellP0014 | 15 | AAFDYADGAAEDELS |
| TcellP0015 | 15 | AAFQAAHARFVAAAA |
| TcellP0016 | 15 | AAFQGAHARFVAAAA |
| TcellP0017 | 15 | AAFSKLPASTIDELK |
| TcellP0018 | 15 | AAFSRMLSLFFRQHI |
| TcellP0019 | 17 | AAFYAQFVQALSAGGGA |
| TcellP0020 | 15 | AAFYRLSSLRLWPDR |
| TcellP0021 | 15 | AAGAQLLWQLPLLSI |
| TcellP0022 | 20 | AAGFASKTPANQAISMIDGP |
| TcellP0023 | 15 | AAGGHNAVFNFPPNG |
| TcellP0024 | 15 | AAGGWDSLAAELATT |
| TcellP0025 | 7 | AAGNVNI |
| TcellP0026 | 25 | AAGSSATGGAAPVGAGAMGQGAQSG |
| TcellP0027 | 15 | AAGTAAQAAVVRFQE |
| TcellP0028 | 17 | AAGTAAQAAVVRFQEAA |
| TcellP0029 | 25 | AAGTAAQAAVVRFQEAANKQKQELD |
| TcellP0030 | 15 | AAGTYVAADAAAASS |
| TcellP0031 | 15 | AAGTYVAADAAAAST |
| TcellP0032 | 15 | AAGVAAWSLIALMIP |
| TcellP0033 | 25 | AAGWQTLSAALDAQAVELTARLNSL |
| TcellP0034 | 15 | AAHAGEYGQMVTLRG |
| TcellP0035 | 9 | AAHARFVAA |
| TcellP0036 | 15 | AAIGLSMAGSSAMIL |
| TcellP0037 | 20 | AAIGLSMAGSSAMILAAYHP |
| TcellP0038 | 21 | AAIGLSMAGSSAMILAAYHPQ |
| TcellP0039 | 9 | AAIGNMTLL |
| TcellP0040 | 15 | AAIHEMFVNTLQMSS |
| TcellP0041 | 15 | AAIHEMFVNTLVASS |
| TcellP0042 | 15 | AAKLAGLVFPQPPAP |
| TcellP0043 | 18 | AALAMEVYQAETAVNTLF |
| TcellP0044 | 15 | AALPAVGAAAGAPAA |
| TcellP0045 | 15 | AALPLLFFALAGQRI |
| TcellP0046 | 15 | AANKQKQELDEISTN |
| TcellP0047 | 20 | AANKQKQELDEISTNIRQAG |
| TcellP0048 | 25 | AANKQKQELDEISTNIRQAGVQYSR |
| TcellP0049 | 9 | AAPEPVARR |
| TcellP0050 | 15 | AAQAAVVRFQEAANK |
| TcellP0051 | 11 | AAQARPVKTVI |
| TcellP0052 | 20 | AAQAVTGETLRATVERALQQ |
| TcellP0053 | 15 | AAQEMMIALRRLREL |
| TcellP0054 | 11 | AAQHRQIVADF |
| TcellP0055 | 15 | AAQHRQIVADFCDFL |
| TcellP0056 | 11 | AAQYRPDELAR |
| TcellP0057 | 15 | AARLLSIRAMSTKFS |
| TcellP0058 | 9 | AASAAIANR |
| TcellP0059 | 15 | AASAIQGNVTSIHSL |
| TcellP0060 | 15 | AASLLDEDMDALEEA |
| TcellP0061 | 20 | AASSVGSVITRLSTEHWMGP |
| TcellP0062 | 20 | AATGIAAVLTDGNPPEVKSV |
| TcellP0063 | 24 | AATGIAAVLTDGNPPEVKSVGLGN |
| TcellP0064 | 15 | AATGYASVIAELTGA |
| TcellP0065 | 15 | AATIDQLKTDAKLLS |
| TcellP0066 | 15 | AATQARAAAAAFEAA |
| TcellP0067 | 15 | AAVALGFFVWLEGRA |
| TcellP0068 | 15 | AAVDKDAVIVAAAGN |
| TcellP0069 | 16 | AAVEEGIVAGGGVTLL |
| TcellP0070 | 9 | AAVENVVDT |
| TcellP0071 | 15 | AAVIVGSGRIASLYV |
| TcellP0072 | 15 | AAVPAVGAAAGAPAA |
| TcellP0073 | 24 | AAVQAAWAVLVAYFNRLRCGTGDY |
| TcellP0074 | 15 | AAVVLPGLVGLAGGA |
| TcellP0075 | 20 | AAVVLPGLVGLAGGAATAGA |
| TcellP0076 | 14 | AAVVRFQEAANKQK |
| TcellP0077 | 15 | AAVVRFQEAANKQKQ |
| TcellP0078 | 17 | AAVVRFQEAANKQKQEL |
| TcellP0079 | 16 | AAWGGSGSEAYQGVQQ |
| TcellP0080 | 18 | AAWGGSGSEAYQGVQQKW |
| TcellP0081 | 20 | AAWGGSGSEAYQGVQQKWDA |
| TcellP0082 | 15 | AAYHPQQFIYAGSLS |
| TcellP0083 | 18 | AAYHPQQFIYAGSLSALL |
| TcellP0084 | 11 | ACFTRPARWTL |
| TcellP0085 | 15 | ACGKAGCQTYKWETL |
| TcellP0086 | 15 | ADEEQQQALSSQMGF |
| TcellP0087 | 15 | ADKNPLFLDEQLTRA |
| TcellP0088 | 20 | ADLGNASVVGRLSVPASWST |
| TcellP0089 | 15 | ADLILLYLIQHCPDL |
| TcellP0090 | 13 | ADLVPTATLLDTY |
| TcellP0091 | 15 | ADQILRETLLTVSSD |
| TcellP0092 | 15 | ADSSKYMITLHTPIA |
| TcellP0093 | 15 | ADVLTMKAVRAATAL |
| TcellP0094 | 15 | ADYLRMWIQAATVMS |
| TcellP0095 | 15 | ADYNMLLISRLREEA |
| TcellP0096 | 20 | AEADADIASGRTYGEDEIRA |
| TcellP0097 | 15 | AEAPAAAAAPEEQVQ |
| TcellP0098 | 15 | AEFLENFVRSSNLKF |
| TcellP0099 | 18 | AEFLENFVRSSNLKFQDA |
| TcellP0100 | 15 | AEHQAIIRDVLTASD |
| TcellP0101 | 15 | AEHQAIISDVLTASD |
| TcellP0102 | 15 | AEHQAIVRDVLAAGD |
| TcellP0103 | 15 | AEHQAIVRDVLAASD |
| TcellP0104 | 15 | AEKFKEDVINDFVSS |
| TcellP0105 | 20 | AEKPATEQAEPVHEVTNDDQ |
| TcellP0106 | 16 | AEKVRNLPAGHGLNAQ |
| TcellP0107 | 15 | AELFRLQTEFVKLQE |
| TcellP0108 | 15 | AELMILIATNLLGQN |
| TcellP0109 | 8 | AEMKTDAA |
| TcellP0110 | 10 | AEMKTDAATL |
| TcellP0111 | 11 | AEMKTDAATLA |
| TcellP0112 | 9 | AEMWAQDAA |
| TcellP0113 | 20 | AERLHVTPSAVSQRIKSLEQ |
| TcellP0114 | 20 | AETPGCVAYIGISFLDQASQ |
| TcellP0115 | 15 | AEVIRLIRRLLPALV |
| TcellP0116 | 15 | AFDKLPAATIDQLKT |
| TcellP0117 | 15 | AFIFADLLILPILNI |
| TcellP0118 | 9 | AFLIGYGLL |
| TcellP0119 | 15 | AFNEILRRRAATAVA |
| TcellP0120 | 8 | AFPSFAGL |
| TcellP0121 | 9 | AFRNIVNML |
| TcellP0122 | 20 | AGAAMLLTPGTAACNRDDVE |
| TcellP0123 | 15 | AGAMGAYAAAEAANA |
| TcellP0124 | 20 | AGARPAASPLAAPVDPSTPA |
| TcellP0125 | 15 | AGCQTYKWETFLTSE |
| TcellP0126 | 15 | AGDQSIGDLIAEAMD |
| TcellP0127 | 15 | AGGFGGAGAGIANFL |
| TcellP0128 | 15 | AGGGVTLLQAAPTLD |
| TcellP0129 | 20 | AGGGVTLLQAAPTLDELKLE |
| TcellP0130 | 20 | AGGSTGSLLMALLGDAGVPF |
| TcellP0131 | 15 | AGGVAVIKAGAATEV |
| TcellP0132 | 15 | AGGYKAADMWGPSSD |
| TcellP0133 | 20 | AGGYKAADMWGPSSDPAWER |
| TcellP0134 | 20 | AGGYKASDMWGPKEDPAWQR |
| TcellP0135 | 15 | AGIEAAASAIQGNVT |
| TcellP0136 | 16 | AGIEAAASAIQGNVTS |
| TcellP0137 | 21 | AGIERTFVAYLKMAGKTAQDT |
| TcellP0138 | 15 | AGISSLIIDPNPMFV |
| TcellP0139 | 15 | AGKPLLIIAEDVEGE |
| TcellP0140 | 21 | AGNVNIAIGGAATGIAAVLTD |
| TcellP0141 | 20 | AGQAELTAAQVRVAAAAYET |
| TcellP0142 | 15 | AGRVAQIRQEIENSD |
| TcellP0143 | 15 | AGSLQGQWRGAAGTA |
| TcellP0144 | 15 | AGSLSALLDPSQGMG |
| TcellP0145 | 15 | AGTAAQAAVVRFQEA |
| TcellP0146 | 18 | AGTAAQAAVVRFQEAANK |
| TcellP0147 | 15 | AGTAVQDSRSHVYAH |
| TcellP0148 | 15 | AGTLSTFFGVPLVLT |
| TcellP0149 | 12 | AGTVNIGASDAY |
| TcellP0150 | 15 | AGWLAFFRDLVARGL |
| TcellP0151 | 16 | AGYAGTLQSLGADIAS |
| TcellP0152 | 15 | AGYPAELAYFEVLHE |
| TcellP0153 | 16 | AHAGDMAGYAGTLQSL |
| TcellP0154 | 19 | AHAGTHVDADVLLSEQAWR |
| TcellP0155 | 15 | AHESLCFAGANLIPL |
| TcellP0156 | 15 | AHGETVSAVAELIGD |
| TcellP0157 | 15 | AHLIHFAAANLRNPG |
| TcellP0158 | 16 | AHLRAFRAYAAHSQEI |
| TcellP0159 | 22 | AHVHISQAEVVVNQLDTMFVAE |
| TcellP0160 | 18 | AIAAGLNAPRRNRVGRQH |
| TcellP0161 | 25 | AIAIAFLAGCSSTKPVSQDTSPKPA |
| TcellP0162 | 20 | AIDTAAANQLMNNVPQALQQ |
| TcellP0163 | 9 | AILIRVRNA |
| TcellP0164 | 15 | AILRRRRRIAEPATC |
| TcellP0165 | 16 | AILTGGQVISEEGVGL |
| TcellP0166 | 15 | AILTGGQVISEEVGL |
| TcellP0167 | 15 | AINGDFILIAPEVQE |
| TcellP0168 | 20 | AIPPRGTQAVVLKVYQNAGG |
| TcellP0169 | 9 | AIQGNVTSI |
| TcellP0170 | 15 | AIQGNVTSIHSLLDE |
| TcellP0171 | 16 | AIQGNVTSIHSLLDEG |
| TcellP0172 | 17 | AIQGNVTSIHSLLDEGK |
| TcellP0173 | 18 | AIRRAYAEMVATSHEIDD |
| TcellP0174 | 15 | AISVTAYALAAEVVP |
| TcellP0175 | 15 | AITILLLVILLIIYG |
| TcellP0176 | 9 | AIVNNRQKD |
| TcellP0177 | 9 | AIYDTMQYV |
| TcellP0178 | 15 | AKAAVEEGIVAGGGV |
| TcellP0179 | 20 | AKKVAKKAPAKKATKAAKKA |
| TcellP0180 | 15 | AKLMRDIPFRVGAVV |
| TcellP0181 | 20 | AKQYRFVLPGPSLTVAEQEQ |
| TcellP0182 | 13 | AKVNIKPLEDKIL |
| TcellP0183 | 9 | ALADAVKVT |
| TcellP0184 | 9 | ALADLPVTV |
| TcellP0185 | 25 | ALAMEVYQAETAVNTLFEKLEPMAS |
| TcellP0186 | 15 | ALCLRLLTARSRTRA |
| TcellP0187 | 9 | ALDEGLLPV |
| TcellP0188 | 10 | ALEAFAIAVA |
| TcellP0189 | 15 | ALEDLVRAYQSMSGT |
| TcellP0190 | 16 | ALEDLVRAYQSMSGTH |
| TcellP0191 | 15 | ALEMFYDDDADLSII |
| TcellP0192 | 20 | ALGANINQLMTLQDYLATVI |
| TcellP0193 | 10 | ALGENGNGGM |
| TcellP0194 | 15 | ALGERRLVRLLRLGG |
| TcellP0195 | 9 | ALGGGATGV |
| TcellP0196 | 9 | ALLALTRAI |
| TcellP0197 | 20 | ALLALTRAILIRVRNASWQH |
| TcellP0198 | 20 | ALLDPSQGMGLIGLAMGDAG |
| TcellP0199 | 15 | ALLDPSQGMGPSLIG |
| TcellP0200 | 20 | ALLDPSQGMGPSLIGLAMGD |
| TcellP0201 | 25 | ALLESEALAEMGGNASLKRTRITIA |
| TcellP0202 | 20 | ALLGDAGVPFRVIPIAASTR |
| TcellP0203 | 9 | ALLGGLRPV |
| TcellP0204 | 15 | ALLPRAGAAAAAALP |
| TcellP0205 | 15 | ALLWLADQVDAALEK |
| TcellP0206 | 15 | ALPRLLRRLVIMGGM |
| TcellP0207 | 9 | ALQNAASIA |
| TcellP0208 | 15 | ALQNLARTISEAGQA |
| TcellP0209 | 15 | ALQSHDDVALVSVMW |
| TcellP0210 | 15 | ALRILVYSDNVQTRE |
| TcellP0211 | 20 | ALRRLKGFDQILKLMSGMLR |
| TcellP0212 | 15 | ALSAEYAAVAQELSV |
| TcellP0213 | 25 | ALSGQLNPQVNLVDTLNSGQYTVFA |
| TcellP0214 | 15 | ALSRVHSMFLGTGGS |
| TcellP0215 | 15 | ALSRVQSMFLGTGGS |
| TcellP0216 | 10 | ALSTLVVNKI |
| TcellP0217 | 15 | ALSTLVVNKIRGTFK |
| TcellP0218 | 15 | ALSVLVGLTAATVAI |
| TcellP0219 | 15 | ALTALIRDPPADSTG |
| TcellP0220 | 25 | ALTEMDYFIRMWNQAALAMEVYQAE |
| TcellP0221 | 15 | ALVAEGIEAIVFRTL |
| TcellP0222 | 15 | ALVEEYLRGLRQAAG |
| TcellP0223 | 19 | ALYLLDGLRAQDDFSGWDI |
| TcellP0224 | 9 | AMAGSIDLL |
| TcellP0225 | 9 | AMASTEGNV |
| TcellP0226 | 15 | AMDYTTIVAAAASES |
| TcellP0227 | 9 | AMEDLVRAY |
| TcellP0228 | 18 | AMEDLVRAYHAMSSTHEA |
| TcellP0229 | 9 | AMEYFRQVL |
| TcellP0230 | 20 | AMFGYAATAATATEALLPFE |
| TcellP0231 | 9 | AMGDAGGYK |
| TcellP0232 | 15 | AMGPTLIGLAMGDAG |
| TcellP0233 | 18 | AMGQGAQSGGSTRPGLVA |
| TcellP0234 | 15 | AMHLLLNMWALYVVG |
| TcellP0235 | 13 | AMKGDLQSSLGAG |
| TcellP0236 | 9 | AMLGHAGDM |
| TcellP0237 | 9 | AMMARDTAE |
| TcellP0238 | 15 | AMNEAFVAMLAPARI |
| TcellP0239 | 15 | AMNEAFVAMLGASAD |
| TcellP0240 | 15 | AMSGLLDPSQAMGPT |
| TcellP0241 | 18 | AMSSTHEANTMAMMARDT |
| TcellP0242 | 9 | AMVNTTTRL |
| TcellP0243 | 15 | ANATVYMIDSVLMPP |
| TcellP0244 | 16 | ANEAETTTASGLVIPD |
| TcellP0245 | 20 | ANKGVSPTGNAAVGLSMSGG |
| TcellP0246 | 9 | ANNTRLWVY |
| TcellP0247 | 15 | ANRAVKPTGSAAIGL |
| TcellP0248 | 20 | ANRAVKPTGSAAIGLSMAGS |
| TcellP0249 | 20 | ANRHVKPTGSAVVGLSMAAS |
| TcellP0250 | 20 | ANSPALYLLDGLRAQDDFSG |
| TcellP0251 | 9 | ANTMAMMAR |
| TcellP0252 | 20 | APDGYPIINYEYAIVNNRQK |
| TcellP0253 | 14 | APIVMVDSVEHLDL |
| TcellP0254 | 20 | APKTYCEELKGTDTGQACQI |
| TcellP0255 | 10 | APLKQIAFNS |
| TcellP0256 | 15 | APLKQIAFNSGLEPG |
| TcellP0257 | 15 | APPNLIRAILRAPVD |
| TcellP0258 | 15 | APQINFFYYLGEPIV |
| TcellP0259 | 15 | APTLDELKLEGDEAT |
| TcellP0260 | 16 | APTLDELKLEGDEATG |
| TcellP0261 | 20 | APTLDELKLEGDEATGANIV |
| TcellP0262 | 15 | APTTGVVPAAADEVS |
| TcellP0263 | 15 | APVVILAHGFPELAY |
| TcellP0264 | 15 | APWQQVLRNLGIDIG |
| TcellP0265 | 15 | APYNVRRLPPPVVEP |
| TcellP0266 | 15 | APYVAWMRATAIQAE |
| TcellP0267 | 15 | AQAAVVRFQEAANKQ |
| TcellP0268 | 20 | AQAAVVRFQEAANKQKQELD |
| TcellP0269 | 15 | AQAVYDFRSIVDYLR |
| TcellP0270 | 15 | AQEYQALSAQAAAFH |
| TcellP0271 | 15 | AQFFASIAQQLTFGP |
| TcellP0272 | 21 | AQFVAATGRPLIGNGANGAPG |
| TcellP0273 | 15 | AQIIHRITATARHPG |
| TcellP0274 | 15 | AQIYQAVSAQAAAIH |
| TcellP0275 | 15 | AQLGYTIRQLERLLQ |
| TcellP0276 | 9 | AQLLTEFAI |
| TcellP0277 | 15 | AQLSQLISLLPSTLQ |
| TcellP0278 | 20 | AQNGVQAMSSLGSQLGSSLG |
| TcellP0279 | 15 | AQNGVQAMSSLGSSL |
| TcellP0280 | 9 | AQQVNYNLP |
| TcellP0281 | 10 | AQQVNYNLPG |
| TcellP0282 | 15 | AQTSQFVMAMINYED |
| TcellP0283 | 15 | AQTVEDEARRMWASA |
| TcellP0284 | 25 | AQYDQYRARNVAVMNAYVSWTRSAL |
| TcellP0285 | 10 | ARDVLAVVSK |
| TcellP0286 | 15 | ARFHQQFVQALTASV |
| TcellP0287 | 15 | ARILRQLATPISVII |
| TcellP0288 | 15 | ARIVIFFVGSVFLLT |
| TcellP0289 | 8 | ARLAGIPY |
| TcellP0290 | 18 | ARLMAGAGPAPMLAAAAG |
| TcellP0291 | 25 | ARLMAGAGPAPMLAAAAGWQTLSAA |
| TcellP0292 | 15 | ARMWIQAATTMASYQ |
| TcellP0293 | 15 | ARRMWASAQNISGAG |
| TcellP0294 | 15 | ARTDLLAFTAFPKQI |
| TcellP0295 | 15 | ARTISEAGQAMASTE |
| TcellP0296 | 16 | ARTISEAGQAMASTEG |
| TcellP0297 | 19 | ARTISEAGQAMASTEGNVT |
| TcellP0298 | 15 | ARVFLDSVLPALGEE |
| TcellP0299 | 10 | ARVIMRSAIG |
| TcellP0300 | 15 | ARVQIHRANDQVRIY |
| TcellP0301 | 18 | ASAAALAGDAAGAWRTAA |
| TcellP0302 | 20 | ASAFARQVGLSHLISDVADA |
| TcellP0303 | 15 | ASAIQGNVTSIHSLL |
| TcellP0304 | 20 | ASATKDGSHYKITGTATGVD |
| TcellP0305 | 16 | ASGLVIPDTAKEKPQE |
| TcellP0306 | 11 | ASHQRQRAFAQ |
| TcellP0307 | 15 | ASIIRLVGAVLAEQH |
| TcellP0308 | 20 | ASLEEGLACAILGVPVADLI |
| TcellP0309 | 20 | ASMLGTVTNSPGVPAVPWGA |
| TcellP0310 | 11 | ASMNRPNLVAL |
| TcellP0311 | 15 | ASMSMAAAASPYVGW |
| TcellP0312 | 9 | ASPVAQSYL |
| TcellP0313 | 25 | ASQSTTNPIFGMPSPGSSTPVGQLP |
| TcellP0314 | 15 | ASSGSYAATEAANAA |
| TcellP0315 | 15 | ASVGSYAAAEAANAS |
| TcellP0316 | 15 | ASVHVLLSHLHADHC |
| TcellP0317 | 15 | ASVIAELTGAPWVGA |
| TcellP0318 | 16 | ASVTVTGQGNSLKVGN |
| TcellP0319 | 20 | ATAAVENVVDTIVRAVHKGD |
| TcellP0320 | 15 | ATAGAFSRPGLPVEY |
| TcellP0321 | 17 | ATATELNNALQNLARTI |
| TcellP0322 | 15 | ATELNNALQNLARTI |
| TcellP0323 | 24 | ATELNNALQNLARTISEAGQAMAS |
| TcellP0324 | 15 | ATEVVRRLTATAHRG |
| TcellP0325 | 26 | ATFRTIIVVGALISMFGINVAASFGA |
| TcellP0326 | 18 | ATGGAAPVGAGAMGQGAQ |
| TcellP0327 | 15 | ATGGGAYAAAEAASV |
| TcellP0328 | 15 | ATGIVLMLGDQPQVA |
| TcellP0329 | 20 | ATGVDMANPMSPVNKSFEIE |
| TcellP0330 | 25 | ATIPQGEQSTGKIYFDVTGPSPTIV |
| TcellP0331 | 18 | ATLAQEAGNFERISGDLK |
| TcellP0332 | 20 | ATMITFRLRLPCRTILRVFS |
| TcellP0333 | 14 | ATNFFGINTIPIAL |
| TcellP0334 | 16 | ATNFFGINTIPIALTE |
| TcellP0335 | 20 | ATNFFGINTIPIALTEMDYF |
| TcellP0336 | 11 | ATQYRPDQLAK |
| TcellP0337 | 25 | ATTAGIPLLRLAAQTALLESEALAE |
| TcellP0338 | 10 | ATVLAQALVR |
| TcellP0339 | 15 | ATVLAQALVREGLRN |
| TcellP0340 | 25 | AVAASNNPELTTLTAALSGQLNPQV |
| TcellP0341 | 9 | AVADHVAAV |
| TcellP0342 | 9 | AVAGAAILV |
| TcellP0343 | 17 | AVAGAAILVAGLSGCSS |
| TcellP0344 | 15 | AVDAVFVGSCTNGRI |
| TcellP0345 | 15 | AVDGRFAVPQILGDE |
| TcellP0346 | 15 | AVHVWLRLPAGRVEI |
| TcellP0347 | 11 | AVINTTCNYGQ |
| TcellP0348 | 15 | AVITELIAMLRHHHI |
| TcellP0349 | 15 | AVLEDPYILLVSSKV |
| TcellP0350 | 15 | AVLLEKIVADEEEHI |
| TcellP0351 | 16 | AVLSSAWQGDTGITYQ |
| TcellP0352 | 15 | AVLVATNFFGINTIP |
| TcellP0353 | 15 | AVPLRLLGGLHRMVL |
| TcellP0354 | 16 | AVRNAKAAVEEGIVAG |
| TcellP0355 | 15 | AVSIGILLSLIAPLG |
| TcellP0356 | 25 | AVVELGSFDAAAERLHVTPSAVSQR |
| TcellP0357 | 20 | AVVGLSMAASSALTLAIYHP |
| TcellP0358 | 20 | AVVLLCCSGVATAAPKTYCE |
| TcellP0359 | 13 | AVVRFQEAANKQK |
| TcellP0360 | 15 | AVVRFQEAANKQKQE |
| TcellP0361 | 15 | AVVVLKRLPDALADG |
| TcellP0362 | 15 | AVWAFVMVLAFSRHL |
| TcellP0363 | 9 | AVYLLDGLR |
| TcellP0364 | 24 | AWGGSGSEAYQGVQQKWDATATEL |
| TcellP0365 | 15 | AWMSAAAAQAEQAAT |
| TcellP0366 | 18 | AWRTAAVELARALVRAVA |
| TcellP0367 | 17 | AWYRLTKFFGKLFLINF |
| TcellP0368 | 15 | AYAQRVYQANRAAGS |
| TcellP0369 | 20 | AYDEEARRGLERGLNALADA |
| TcellP0370 | 20 | AYGLTVPPPVIAENRAELMI |
| TcellP0371 | 15 | AYGSFVRTVSLPVGA |
| TcellP0372 | 9 | AYHPQQFIY |
| TcellP0373 | 9 | AYLVVKTLI |
| TcellP0374 | 15 | AYNAGGGHNGVFDFP |
| TcellP0375 | 9 | AYQGVQQKW |
| TcellP0376 | 15 | AYRTTIFAFPVFGFG |
| TcellP0377 | 9 | AYVPYVAWL |
| TcellP0378 | 26 | AYVSWTRSALSDLPRWREPPQIYRGG |
| TcellP0379 | 15 | CGNGTPNELGGANIP |
| TcellP0380 | 20 | CGNGTPNELGGANIPAEFLE |
| TcellP0381 | 20 | CGSKPPSGSPETGAGAGTVA |
| TcellP0382 | 15 | CILAWILVRIINVRS |
| TcellP0383 | 20 | CLDELRGAAASAAFVVASGS |
| TcellP0384 | 15 | CLGSHLARLELTLLV |
| TcellP0385 | 20 | CQIQMSDPAYNINISLPSYY |
| TcellP0386 | 15 | CQMSFVVTIPEALAA |
| TcellP0387 | 9 | CQTYKWETF |
| TcellP0388 | 15 | CRGYDVVILDRYVAS |
| TcellP0389 | 20 | CRQQKGHVFEGGLWSGGAAN |
| TcellP0390 | 15 | CRRYEVLLIFDEIAT |
| TcellP0391 | 15 | CSEDLLYLSDLDFDV |
| TcellP0392 | 16 | CTTAAGNVNIAIGGAA |
| TcellP0393 | 18 | DAATAQTLQAFLHWAITD |
| TcellP0394 | 19 | DAATAQTLQAFLHWAITDG |
| TcellP0395 | 20 | DAATAQTLQAFLHWAITDGN |
| TcellP0396 | 15 | DAHGAMIRALAGLLE |
| TcellP0397 | 15 | DAHGAMIRAQAASLE |
| TcellP0398 | 15 | DAHGAMIRAQAGLLE |
| TcellP0399 | 13 | DALRLARRIAAAL |
| TcellP0400 | 20 | DAPLITNPGGLLEQAVAVEE |
| TcellP0401 | 10 | DAVRNAKAAV |
| TcellP0402 | 11 | DAVRNAKAAVE |
| TcellP0403 | 20 | DCGTIRVGSFRGRWLDPRHA |
| TcellP0404 | 15 | DDGAIDILLVGLDSR |
| TcellP0405 | 20 | DDIKATYDKGILTVSVAVSE |
| TcellP0406 | 15 | DDIPRVLAEAFHIAA |
| TcellP0407 | 13 | DDLVGAGVIDAVA |
| TcellP0408 | 15 | DDYNELVISVPLQLT |
| TcellP0409 | 15 | DDYNGWDINTPAFEW |
| TcellP0410 | 18 | DDYNGWDINTPAFEWYYQ |
| TcellP0411 | 15 | DEARRMWASAQNISG |
| TcellP0412 | 20 | DEGAGASPGQQPGGGVPAQA |
| TcellP0413 | 15 | DEISTNIRQAGVQYS |
| TcellP0414 | 25 | DELDELDENWKGSSSDLLADAVERY |
| TcellP0415 | 18 | DEQRRLEAGMKLGWHPYH |
| TcellP0416 | 15 | DESWQQFRQELIPLL |
| TcellP0417 | 15 | DFDGRSEFAYGSFVR |
| TcellP0418 | 15 | DFFVAADSAFSSLND |
| TcellP0419 | 17 | DFNRDSREVVHLATGMA |
| TcellP0420 | 15 | DGDRHARGFEDLVEV |
| TcellP0421 | 25 | DGISTTKITGTIPASSVKMLDPGAK |
| TcellP0422 | 15 | DGLLAILAAGASLVQ |
| TcellP0423 | 15 | DGLVLNFDDYNELVI |
| TcellP0424 | 20 | DGNPPEVKSVGLGNVNGVTL |
| TcellP0425 | 15 | DGRSEFAYGSFVRTV |
| TcellP0426 | 15 | DGRTARVPGDEITST |
| TcellP0427 | 15 | DGTFSRMLSWAQIGV |
| TcellP0428 | 9 | DGYVGAPAH |
| TcellP0429 | 24 | DGYVRFCVMAPRQWRGLRRWLGEP |
| TcellP0430 | 20 | DHEGVIDSNTTATSAPPRTK |
| TcellP0431 | 15 | DHGGVIFIDLRDASG |
| TcellP0432 | 16 | DIASEQAVLSSAWQGD |
| TcellP0433 | 20 | DIASEQAVLSSAWQGDTGIT |
| TcellP0434 | 20 | DIASERTAPSRACQGDLGMS |
| TcellP0435 | 15 | DIGCVFSIDTDAHAP |
| TcellP0436 | 22 | DIGLHIIVTCQMSQAYKATMDK |
| TcellP0437 | 20 | DIKRATDTVAGISGRVQLTH |
| TcellP0438 | 18 | DIKVQFQGGGPHAVYLLD |
| TcellP0439 | 9 | DIKVQFQSG |
| TcellP0440 | 20 | DIKVQFQSGGANSPALYLLD |
| TcellP0441 | 15 | DIKVQFQSGGNNSPA |
| TcellP0442 | 20 | DIKVQFQSGGNNSPAVYLLD |
| TcellP0443 | 15 | DKILVQANEAETTTA |
| TcellP0444 | 20 | DKVLPLVAIVVSVGLAVSYD |
| TcellP0445 | 15 | DLDSYLVEITAEVLR |
| TcellP0446 | 15 | DLKLLRETPNAVRRS |
| TcellP0447 | 25 | DLLADAVERYLQWLSKHSSQLKHAA |
| TcellP0448 | 16 | DLSLLGKARKVVVTKD |
| TcellP0449 | 20 | DLSLLGKARKVVVTKDETTI |
| TcellP0450 | 20 | DMANPMSPVNKSFEIEVTCS |
| TcellP0451 | 9 | DMLGRAGGL |
| TcellP0452 | 15 | DMTKIVGLRARAKAA |
| TcellP0453 | 9 | DMWEHAFYL |
| TcellP0454 | 10 | DMWEHAFYLQ |
| TcellP0455 | 15 | DNGVGYVGLVASTVR |
| TcellP0456 | 15 | DNLVRTISLQPTDGL |
| TcellP0457 | 23 | DNVAGKRVHNAIIRRMQRMSFEE |
| TcellP0458 | 20 | DPDKDVDIMVRDGQLTIKAE |
| TcellP0459 | 13 | DPIELNATLSAVA |
| TcellP0460 | 15 | DPINEFFLANTGRPL |
| TcellP0461 | 15 | DPMVQIPRLVANNTR |
| TcellP0462 | 15 | DPVKGADEVVAFAEE |
| TcellP0463 | 20 | DQITITKNGAPAAVLVGADE |
| TcellP0464 | 19 | DQSIGDLIAEAMDKVGNEG |
| TcellP0465 | 15 | DQVESTAGSLQGQWR |
| TcellP0466 | 18 | DQVESTAGSLQGQWRGAA |
| TcellP0467 | 19 | DQVHFQPLPPAVVKLSDAL |
| TcellP0468 | 20 | DQVHFQPLPPAVVKLSDALI |
| TcellP0469 | 15 | DRMIHYRTIDSPIGP |
| TcellP0470 | 16 | DRRKAMLQDMAILTGG |
| TcellP0471 | 15 | DRSRIEFAITILLLV |
| TcellP0472 | 15 | DRWLDLRYVGPASAD |
| TcellP0473 | 25 | DRYRHLVALSITDFGAAGPRSSWRA |
| TcellP0474 | 15 | DSFFHLAPLGQSGAL |
| TcellP0475 | 15 | DSGTHSWEYWGAQLN |
| TcellP0476 | 15 | DSTVITDGDIVNIDV |
| TcellP0477 | 15 | DSTVITDRDIVNIDV |
| TcellP0478 | 20 | DTDPLPVVFPIVQGELSKQT |
| TcellP0479 | 15 | DTEVYLDAASLSPFV |
| TcellP0480 | 9 | DTFLFTQYL |
| TcellP0481 | 15 | DTFYDRAQEYSQVLQ |
| TcellP0482 | 15 | DTGITYQGWQTQWNQ |
| TcellP0483 | 15 | DTQSMIVTDHRYVPA |
| TcellP0484 | 9 | DVAAMSGYY |
| TcellP0485 | 20 | DVDAHGAMIRAQAGSLEAEH |
| TcellP0486 | 15 | DVEGEALSTLVVNKI |
| TcellP0487 | 16 | DVEGEALSTLVVNKIR |
| TcellP0488 | 15 | DVIHAFWVPEFLFKR |
| TcellP0489 | 20 | DVLHAFNPRIVLAGSSAFGN |
| TcellP0490 | 20 | DVQPAEVVAAARDEGAGASP |
| TcellP0491 | 24 | DVTGPSPTIVAMNNGMEDLLIWEP |
| TcellP0492 | 15 | DVTIRFRRFFSRLQR |
| TcellP0493 | 15 | DWPAAYAIGEHLSVE |
| TcellP0494 | 20 | DWPAAYAIGEHLSVEIAVAV |
| TcellP0495 | 20 | DWYQPACGKAGCQTYKWETF |
| TcellP0496 | 20 | DWYQPSQSNGQNYTYKWETF |
| TcellP0497 | 15 | DWYSPACGKAGCQTY |
| TcellP0498 | 20 | DWYSPACGKAGCQTYKWETF |
| TcellP0499 | 20 | DWYSPACGKAGCQTYKWETL |
| TcellP0500 | 15 | DYVRMWVQAATAMSA |
| TcellP0501 | 15 | DYVRMWVQAATVMSA |
| TcellP0502 | 15 | DYVYNIKANPAVRVR |
| TcellP0503 | 24 | EAAASAIQGNVTSIHSLLDEGKQS |
| TcellP0504 | 15 | EAGQAMASTEGNVTG |
| TcellP0505 | 16 | EAPLKQIAFNSGLEPG |
| TcellP0506 | 20 | EARHVIPFSALCLRLSQLGG |
| TcellP0507 | 15 | EARRMWASAQNISGA |
| TcellP0508 | 16 | EATGANIVKVALEAPL |
| TcellP0509 | 16 | EAYQGVQQKWDATATE |
| TcellP0510 | 17 | EAYQGVQQKWDATATEL |
| TcellP0511 | 15 | EDEARRMWASAQNIS |
| TcellP0512 | 20 | EDGPIAVMPPAPGMVVAANS |
| TcellP0513 | 15 | EDLVRAYHAMSSTHE |
| TcellP0514 | 15 | EDLVRAYHSMSSTHE |
| TcellP0515 | 18 | EEAAQMGLLGTSPLSNHP |
| TcellP0516 | 15 | EEGVGLTLENADLSL |
| TcellP0517 | 25 | EEIAANREERRRLIASNVAGVNTPA |
| TcellP0518 | 15 | EEIALIARWLAEPGV |
| TcellP0519 | 15 | EEPRLFYMHYWAVDD |
| TcellP0520 | 15 | EESNTFGLQLELTEG |
| TcellP0521 | 16 | EEVGLTLENADLSLLG |
| TcellP0522 | 17 | EEYLILSARDVLAVVSK |
| TcellP0523 | 15 | EFAYGSFVRTVSLPV |
| TcellP0524 | 19 | EFAYGSFVRTVSLPVGADE |
| TcellP0525 | 20 | EFETTRSSTGTGLQGVTSGL |
| TcellP0526 | 9 | EFQTVSNQL |
| TcellP0527 | 17 | EGDEATGANIVKVALEA |
| TcellP0528 | 16 | EGDTVIYSKYGGTEIK |
| TcellP0529 | 15 | EGGNQIVQYLVAQKI |
| TcellP0530 | 12 | EGIQLLASNASQ |
| TcellP0531 | 15 | EGKQSLTKLAAAWGG |
| TcellP0532 | 20 | EGKQSLTKLAAAWGGSGSEA |
| TcellP0533 | 25 | EGKQSLTKLAAAWGGSGSEAYQGVQ |
| TcellP0534 | 25 | EGPGADSLFFASGQLRELAYSVETT |
| TcellP0535 | 15 | EGVSILAESAEFESE |
| TcellP0536 | 13 | EGVVLLLVGALVL |
| TcellP0537 | 15 | EGWWPTLIGLAMNDS |
| TcellP0538 | 15 | EHELYVAVLSNALHR |
| TcellP0539 | 15 | EHFGHERYDAFFSLA |
| TcellP0540 | 15 | EHIHRPNTNNVGPII |
| TcellP0541 | 15 | EHRVKRGLTVAVAGA |
| TcellP0542 | 16 | EIAVEQAALQSAWQGD |
| TcellP0543 | 18 | EIAVEQAALQSAWQGDTG |
| TcellP0544 | 15 | EIGWEAGTAAPDEIP |
| TcellP0545 | 9 | EISTNIRQA |
| TcellP0546 | 15 | EISTNIRQAGVQYSR |
| TcellP0547 | 20 | EISTNIRQAGVQYSRADEEQ |
| TcellP0548 | 15 | EITSTVSGWLSELGT |
| TcellP0549 | 15 | EIVQFLEETFAAYDQ |
| TcellP0550 | 15 | EIYLNTFRHLYGLDC |
| TcellP0551 | 14 | EKIGAELVKEVAKK |
| TcellP0552 | 21 | EKKGWAPTITNDGVSIAKEIE |
| TcellP0553 | 15 | EKMRVAGRIAAGALA |
| TcellP0554 | 15 | EKSYGLLDDNVYTFL |
| TcellP0555 | 9 | ELAAEPTEV |
| TcellP0556 | 15 | ELADLIEFARTVNEE |
| TcellP0557 | 18 | ELALLSMHLDDEQRRLEA |
| TcellP0558 | 20 | ELDEDLVRAAQAVTGETLRA |
| TcellP0559 | 15 | ELDEISTNIRQAGVQ |
| TcellP0560 | 18 | ELDEISTNIRQAGVQYSR |
| TcellP0561 | 15 | ELFVAAYVPYVAWLV |
| TcellP0562 | 15 | ELKERKHRIEDAVRN |
| TcellP0563 | 9 | ELNNALQNL |
| TcellP0564 | 13 | ELNNALQNLARTI |
| TcellP0565 | 15 | ELNNALQNLARTISE |
| TcellP0566 | 15 | ELPGVDPDKDVDIMV |
| TcellP0567 | 10 | ELPQWLSANR |
| TcellP0568 | 15 | ELTGAPWVGAASLSM |
| TcellP0569 | 15 | ELVAAFLWAQFEEAE |
| TcellP0570 | 15 | ELVRADVTTPCLLPE |
| TcellP0571 | 15 | ELVRADVTTPRLLPE |
| TcellP0572 | 9 | EMGRAPLDL |
| TcellP0573 | 9 | EMKTDAATL |
| TcellP0574 | 15 | EMLSMLRAMLAPESL |
| TcellP0575 | 9 | EMLTSRGLL |
| TcellP0576 | 9 | EMVLRADQL |
| TcellP0577 | 15 | ENFFMFIAEEVREYL |
| TcellP0578 | 9 | ENFVRSSNL |
| TcellP0579 | 15 | EPARGRKRTLSDVLN |
| TcellP0580 | 15 | EPEMLDRLDIVVASV |
| TcellP0581 | 20 | EPEQLAAAHELIDRGRAEVV |
| TcellP0582 | 15 | EPGDIIIDGGNALYT |
| TcellP0583 | 9 | EPQTVSNQL |
| TcellP0584 | 15 | EPYAVWLDDWYARES |
| TcellP0585 | 9 | EPYLDPATM |
| TcellP0586 | 25 | EQGVPFRVQGDNISVKLFDDWSNLG |
| TcellP0587 | 15 | EQIAATAAISAGDQS |
| TcellP0588 | 15 | EQMLIDDGILLRKYW |
| TcellP0589 | 9 | EQQWNFAGI |
| TcellP0590 | 15 | EQQWNFAGIEAAASA |
| TcellP0591 | 20 | EQRDRILIITINRPKAKNAV |
| TcellP0592 | 20 | ERLHVTPSAVSQRIKSLEQQ |
| TcellP0593 | 15 | ERYVGLYLPFLDMSF |
| TcellP0594 | 15 | ESALFFIGVLIVALL |
| TcellP0595 | 20 | ESFTVNESRTAKQYRFVLPG |
| TcellP0596 | 15 | ESGASYAARDALAAA |
| TcellP0597 | 15 | ESGEEVAGEETRIAF |
| TcellP0598 | 15 | ESLVSDGLPGVKAAL |
| TcellP0599 | 20 | ESSALAAAQAMASAAAFETA |
| TcellP0600 | 15 | ESTNTKIRLLTRIAF |
| TcellP0601 | 15 | ESVFAATVAELESLI |
| TcellP0602 | 18 | ETAVNTLFEKLEPMASIL |
| TcellP0603 | 25 | ETLHERIKVTERRLLVAAVAALATH |
| TcellP0604 | 15 | ETLYWLAQPGIRESI |
| TcellP0605 | 20 | ETPGCVAYIGISFLDQASQR |
| TcellP0606 | 15 | ETTTASGLVIPDTAK |
| TcellP0607 | 32 | EVIRILRRRSLAALRAQAEPVSTAAYGRFLPA |
| TcellP0608 | 17 | EVLVEQRDRILIITINR |
| TcellP0609 | 15 | EVNIKILIDSLVSAG |
| TcellP0610 | 9 | EVVANRALL |
| TcellP0611 | 15 | EVVDYLGIPASARPV |
| TcellP0612 | 15 | EWLYQSWAAAYLPLL |
| TcellP0613 | 20 | EWYDQSGLSVVMPVGGQSSF |
| TcellP0614 | 16 | EYLILSARDVLAVVSK |
| TcellP0615 | 15 | EYTVFAPTNAAFDKL |
| TcellP0616 | 25 | EYTVFAPTNAAFDKLPAATIDQLKT |
| TcellP0617 | 20 | EYWGAQLNAMKGDLQSSLGA |
| TcellP0618 | 10 | FAGIEAAASA |
| TcellP0619 | 15 | FAGIEAAASAIQGNV |
| TcellP0620 | 20 | FALTVPPAEVVANRALLAEL |
| TcellP0621 | 15 | FATGMAQFFASIAQQ |
| TcellP0622 | 9 | FAVTNDGVI |
| TcellP0623 | 15 | FAYGSFVRTVSLPVG |
| TcellP0624 | 16 | FAYGSFVRTVSLPVGA |
| TcellP0625 | 15 | FDGRSEFAYGSFVRT |
| TcellP0626 | 15 | FDHEFTFGWDELLSK |
| TcellP0627 | 15 | FDHVPEQAFFLIGGL |
| TcellP0628 | 20 | FDQLSELWKAISPHLSPLSN |
| TcellP0629 | 15 | FDREFTFGWDELLSK |
| TcellP0630 | 20 | FEGGLWSGGAANAANGALGA |
| TcellP0631 | 15 | FEWYDQSGLSVVMPV |
| TcellP0632 | 10 | FFGINTIPIA |
| TcellP0633 | 15 | FFGQNAPAIAAIEAA |
| TcellP0634 | 15 | FFGQNTAAIAATEAQ |
| TcellP0635 | 15 | FFQVLVTQFGSSGGP |
| TcellP0636 | 7 | FGLQLEL |
| TcellP0637 | 20 | FGLQLELTEGMRFDKGYISG |
| TcellP0638 | 18 | FGMPSPGSSTPVGQLPPA |
| TcellP0639 | 15 | FGQNTASIAATEAQY |
| TcellP0640 | 15 | FGQNTGAIAAAEARY |
| TcellP0641 | 15 | FGQNTSAIAAAEAQY |
| TcellP0642 | 15 | FGVIFGAIWAEEAWG |
| TcellP0643 | 15 | FISQMVYSFFASLDR |
| TcellP0644 | 9 | FIYAGSLSA |
| TcellP0645 | 10 | FIYAGSLSAL |
| TcellP0646 | 18 | FIYAGSLSALLDPSQGMG |
| TcellP0647 | 20 | FLAGGPMAVYLLDAFNAGPD |
| TcellP0648 | 15 | FLEGFVRTSNIKFQD |
| TcellP0649 | 15 | FLGQNTAAIAATEAQ |
| TcellP0650 | 9 | FLLGLLFFV |
| TcellP0651 | 20 | FLLKASVRELRECVGSELLT |
| TcellP0652 | 9 | FLLPRGLAI |
| TcellP0653 | 15 | FLLVPVLILLTVSGR |
| TcellP0654 | 15 | FLMSVGALIIGWLLQ |
| TcellP0655 | 17 | FLRIATSARVLAAPLPT |
| TcellP0656 | 9 | FLTSELPQW |
| TcellP0657 | 18 | FLTSELPQWLSANRAVKP |
| TcellP0658 | 10 | FLTTEAVVAD |
| TcellP0659 | 9 | FLYELIWNV |
| TcellP0660 | 9 | FMTRLGPLL |
| TcellP0661 | 9 | FMYEGDTPL |
| TcellP0662 | 19 | FNLWGPAFHERYPNVTITA |
| TcellP0663 | 15 | FPDRASIIRLVGAVL |
| TcellP0664 | 15 | FPPNGTHSWEYWGAQ |
| TcellP0665 | 22 | FPVVTHDEVLRLVGRRRLWGRG |
| TcellP0666 | 15 | FQDAYNAAGGHNAVF |
| TcellP0667 | 21 | FQDAYNAAGGHNAVFNFPPNG |
| TcellP0668 | 15 | FQEAANKQKQELDEI |
| TcellP0669 | 15 | FQSGGNNSPAVYLLD |
| TcellP0670 | 15 | FRVVISSRFGDIFRG |
| TcellP0671 | 15 | FSGWDINTPAFEWYD |
| TcellP0672 | 25 | FSKLPASTIDELKTNSSLLTSILTY |
| TcellP0673 | 15 | FSLVNFFDAQVGPLS |
| TcellP0674 | 15 | FSRPGLPVEYLQVPS |
| TcellP0675 | 20 | FSRPGLPVEYLQVPSASMGR |
| TcellP0676 | 18 | FSRPGLPVEYLQVPSPSM |
| TcellP0677 | 19 | FSRPGLPVEYLQVPSPSMG |
| TcellP0678 | 20 | FSRPGLPVEYLQVPSPSMGR |
| TcellP0679 | 15 | FTQLDMEMSFVDAED |
| TcellP0680 | 11 | FTRRFAASMVG |
| TcellP0681 | 15 | FVDAEDIIAISEEVL |
| TcellP0682 | 13 | FVKAICSPMALAS |
| TcellP0683 | 15 | FVQALTTAAASYASV |
| TcellP0684 | 9 | FVRSSNLKF |
| TcellP0685 | 15 | FVRTVSLPVGADEDD |
| TcellP0686 | 15 | FVYAGAMSGLLDPSQ |
| TcellP0687 | 15 | FYNEKAFLLTTFDVS |
| TcellP0688 | 15 | FYSDWYQPACGKAGC |
| TcellP0689 | 15 | GAAGTAAQAAVVRFQ |
| TcellP0690 | 19 | GAAGTAAQAAVVRFQEAAN |
| TcellP0691 | 23 | GAAGTAAQAAVVRFQEAANKQKQ |
| TcellP0692 | 20 | GAAILVAGLSGCSSNKSTTG |
| TcellP0693 | 20 | GAANAANGALGANINQLMTL |
| TcellP0694 | 20 | GAASGPKVVIDGKDQNVTGS |
| TcellP0695 | 15 | GADATAAAAFEQFLA |
| TcellP0696 | 20 | GADEWESLQETLYWLAQPGI |
| TcellP0697 | 16 | GAGIAQAAAGTVNIGA |
| TcellP0698 | 25 | GAMGQGAQSGGSTRPGLVAPAPLAQ |
| TcellP0699 | 15 | GAMVATNFFGINTIP |
| TcellP0700 | 15 | GANIPAEFLENFVRS |
| TcellP0701 | 20 | GANIPAEFLENFVRSSNLKF |
| TcellP0702 | 20 | GAPAAVLVGADEWESLQETL |
| TcellP0703 | 10 | GAPINSATAM |
| TcellP0704 | 20 | GAPRYDPGGGGINVARIVHV |
| TcellP0705 | 15 | GAQLNAMKPDLQRAL |
| TcellP0706 | 20 | GAQRLPAEGPAVKRGVGASA |
| TcellP0707 | 20 | GASDAYLSEGDMAAHKGLMN |
| TcellP0708 | 18 | GASQSTTNPIFGMPSPGS |
| TcellP0709 | 25 | GASVTVTGQGNSLKVGNADVVCGGV |
| TcellP0710 | 15 | GAYAAAEAANVSAAQ |
| TcellP0711 | 20 | GCQTYKWETFLTSELPGWLQ |
| TcellP0712 | 18 | GCQTYKWETFLTSELPQW |
| TcellP0713 | 20 | GCQTYKWETFLTSELPQWLS |
| TcellP0714 | 15 | GCQTYKWETLLTSEL |
| TcellP0715 | 18 | GCQTYKWETLLTSELPQW |
| TcellP0716 | 20 | GCQTYKWETLLTSELPQWLS |
| TcellP0717 | 20 | GCSSNKSTTGSGETTTAAGT |
| TcellP0718 | 8 | GCVAYIGI |
| TcellP0719 | 16 | GCVAYIGISFLDQASQ |
| TcellP0720 | 15 | GDCLVAFDAPLVVAN |
| TcellP0721 | 15 | GDEATGANIVKVALE |
| TcellP0722 | 15 | GDGTQLQVMISLDKV |
| TcellP0723 | 17 | GDLKTQIDQVESTAGSL |
| TcellP0724 | 15 | GDLRVIILEGQPIHV |
| TcellP0725 | 20 | GDLVGPGCAEYAAANPTGPA |
| TcellP0726 | 25 | GDLVGPGCAEYAAANPTGPASVQGM |
| TcellP0727 | 15 | GEALSTLVVNKIRGT |
| TcellP0728 | 15 | GEEYLILSARDVLAV |
| TcellP0729 | 9 | GEGGVGSIL |
| TcellP0730 | 20 | GELARDSIYYVDANASIQEM |
| TcellP0731 | 20 | GELLPEAAGPTQVLVPRSAI |
| TcellP0732 | 20 | GELSKQTGQQVSIAPNAGLD |
| TcellP0733 | 18 | GEMDLRGVRWVAVSESEK |
| TcellP0734 | 20 | GEMWAQDASAMYGYAAASAV |
| TcellP0735 | 16 | GFASKTPANQAISMID |
| TcellP0736 | 15 | GFGVFEQRRRAARVA |
| TcellP0737 | 20 | GFLNPSEGWWPTLIGLAMND |
| TcellP0738 | 25 | GFTAAAAAKAPSLAWNRDDGLQDML |
| TcellP0739 | 25 | GFTAAARAGLGWGMFPEKLAASPLA |
| TcellP0740 | 15 | GFTIANHNAAAVGEI |
| TcellP0741 | 20 | GFVRTSNIKFQDAYNAGGGH |
| TcellP0742 | 21 | GGFGGVSNALRMPPRAYVMPR |
| TcellP0743 | 25 | GGGSDKALAAATPMVVWLQTASTQA |
| TcellP0744 | 20 | GGGVPAQAMDTGAGARPAAS |
| TcellP0745 | 20 | GGLQHISSGVFLLKASVREL |
| TcellP0746 | 16 | GGLYYIFATYTTTVDF |
| TcellP0747 | 9 | GGPHAVYLL |
| TcellP0748 | 15 | GGQSSFYSDWYQPAC |
| TcellP0749 | 18 | GGQSSFYSDWYSPACGKA |
| TcellP0750 | 20 | GGSGPMPAQLASAEKPATEQ |
| TcellP0751 | 18 | GGSGPSAGAGLLRAESLP |
| TcellP0752 | 18 | GGTGGGNPADEEAAQMGL |
| TcellP0753 | 18 | GGYKAADMWGPSSDPAWE |
| TcellP0754 | 16 | GHRRMVFRFLTSPIEI |
| TcellP0755 | 15 | GHVVRFLEAGSKVKV |
| TcellP0756 | 15 | GIEAAASAIQGNVTS |
| TcellP0757 | 21 | GIEAAASAIQGNVTSIHSLLD |
| TcellP0758 | 20 | GIERSGEVNAKPGTKSGDRV |
| TcellP0759 | 9 | GILTVSVAV |
| TcellP0760 | 15 | GINTIPIAINEAEYV |
| TcellP0761 | 20 | GINVARIVHVLGGCSTALFP |
| TcellP0762 | 15 | GIVALIALGILEHFD |
| TcellP0763 | 20 | GIWVDSAGQLVDEPAPPARA |
| TcellP0764 | 15 | GKAGCQTYKWETFLT |
| TcellP0765 | 15 | GKDGVVAHFVEDLVL |
| TcellP0766 | 15 | GKHVLIIFDDLTKQA |
| TcellP0767 | 15 | GKIVRQKANRRHLLE |
| TcellP0768 | 15 | GKLIANNTRVWVYCG |
| TcellP0769 | 14 | GKPTEKHIQIRSTN |
| TcellP0770 | 19 | GKQSLTKLAAAWGGSGSEA |
| TcellP0771 | 24 | GKQSLTKLAAAWGGSGSEAYQGVQ |
| TcellP0772 | 17 | GKTYDAYFTDAGGITPG |
| TcellP0773 | 25 | GLAALAVAVSPPAAAGDLVGPGCAE |
| TcellP0774 | 9 | GLAGGAATA |
| TcellP0775 | 9 | GLDARAYRL |
| TcellP0776 | 20 | GLDPVNYQNFAVTNDGVIFF |
| TcellP0777 | 9 | GLDSRAYRL |
| TcellP0778 | 10 | GLEPGVVAEK |
| TcellP0779 | 11 | GLEPGVVAEKV |
| TcellP0780 | 12 | GLEPGVVAEKVR |
| TcellP0781 | 16 | GLEPGVVAEKVRNLSV |
| TcellP0782 | 20 | GLGEAQLGNSSGNFLLPDAQ |
| TcellP0783 | 20 | GLGNVNGVTLGYTSGTGQGN |
| TcellP0784 | 15 | GLHFQHTAAVLFGVW |
| TcellP0785 | 10 | GLIDIAPHQI |
| TcellP0786 | 12 | GLIDIAPHQISS |
| TcellP0787 | 13 | GLIDIAPHQISSV |
| TcellP0788 | 17 | GLKRGIEKAVEKVTETL |
| TcellP0789 | 20 | GLLDPSQAMGPTLIGLAMGD |
| TcellP0790 | 25 | GLLGTSPLSNHPLAGGSGPSAGAGL |
| TcellP0791 | 9 | GLLHHAPSL |
| TcellP0792 | 9 | GLPVEYLQV |
| TcellP0793 | 20 | GLRAQDDFSGWDINTPAFEW |
| TcellP0794 | 20 | GLRAQDDYNGWDINTPAFEW |
| TcellP0795 | 12 | GLSMAASSALTL |
| TcellP0796 | 25 | GLVAPAPLAQEREEDDEDDWDEEDD |
| TcellP0797 | 9 | GLWLSVAAV |
| TcellP0798 | 9 | GMFANRWII |
| TcellP0799 | 9 | GMGMVGTAL |
| TcellP0800 | 9 | GMGPSLIGL |
| TcellP0801 | 20 | GMHNEIVGDIKRATDTVAGI |
| TcellP0802 | 16 | GMLRERQHRLLYLASA |
| TcellP0803 | 9 | GMNVTAPAL |
| TcellP0804 | 25 | GNADVVCGGVSTANATVYMIDSVLM |
| TcellP0805 | 15 | GNFERISGDLKTQID |
| TcellP0806 | 25 | GNFERISGDLKTQIDQVESTAGSLQ |
| TcellP0807 | 20 | GNGGMVTGCAETPGCVAYIG |
| TcellP0808 | 20 | GNGKPSDLGGNNLPAKFLEG |
| TcellP0809 | 15 | GNGVVALRNAQLVTF |
| TcellP0810 | 20 | GNNLPAKFLEGFVRTSNIKF |
| TcellP0811 | 12 | GNPNRPDGGILT |
| TcellP0812 | 15 | GNVTSIHSLLDEGKQ |
| TcellP0813 | 20 | GPKEDPAWQRNDPLLNVGKL |
| TcellP0814 | 18 | GPMQQLTQPLQQVTSLFS |
| TcellP0815 | 21 | GPRLYGEMTMQGTRKPRPSGP |
| TcellP0816 | 9 | GPRTRGYAI |
| TcellP0817 | 9 | GPSLIGLAM |
| TcellP0818 | 15 | GPSSDPAWERNDPTQ |
| TcellP0819 | 18 | GPSSDPAWERNDPTQQIP |
| TcellP0820 | 20 | GPSSDPAWERNDPTQQIPKL |
| TcellP0821 | 14 | GQLLRRVRSRLARL |
| TcellP0822 | 15 | GQLLVFDTRRGMVVG |
| TcellP0823 | 15 | GQNYTYKWETFLTRE |
| TcellP0824 | 20 | GQPSQATQLLSTPVSQVTTQ |
| TcellP0825 | 15 | GQQYQAMSAQAAAFH |
| TcellP0826 | 10 | GQSSFYSDWY |
| TcellP0827 | 14 | GQVVGMNTAASDNF |
| TcellP0828 | 15 | GQWRGAAGTAAQAAV |
| TcellP0829 | 20 | GQWRGAAGTAAQAAVVRFQE |
| TcellP0830 | 17 | GRAELSSIVVLLTNNTA |
| TcellP0831 | 18 | GRDIKVQFQSGGNNSPAV |
| TcellP0832 | 25 | GRGCAQYAAQNPTGPGSVAGMAQDP |
| TcellP0833 | 15 | GRLKGILKYYDAPIV |
| TcellP0834 | 13 | GRLRGLFTNPGSW |
| TcellP0835 | 15 | GRSEFAYGSFVRTVS |
| TcellP0836 | 16 | GRWDEDGEKRIPLDVA |
| TcellP0837 | 15 | GSAENFSVVEALADS |
| TcellP0838 | 20 | GSASLVAAAKMWDSVASDLF |
| TcellP0839 | 20 | GSFGSAPSNGWLKLGLVEFG |
| TcellP0840 | 9 | GSFVRTVSL |
| TcellP0841 | 15 | GSFVRTVSLPVGADE |
| TcellP0842 | 15 | GSGHFVKMVHNGIEY |
| TcellP0843 | 25 | GSGPSAGAGLLRAESLPGAGGSLTR |
| TcellP0844 | 20 | GSIRSLARAVVHAANLGVGV |
| TcellP0845 | 25 | GSLIFAAVLVMLIAVLARLMMRGWR |
| TcellP0846 | 15 | GSLQGQWRGAAGTAA |
| TcellP0847 | 15 | GSPLNLLRWTSARSI |
| TcellP0848 | 25 | GSSTPVGQLPPAATQTLGQLGEMSG |
| TcellP0849 | 15 | GSTINAANAAAALPT |
| TcellP0850 | 25 | GSVAGMAQDPVATAASNNPMLSTLT |
| TcellP0851 | 15 | GSYAATEAANAAAAG |
| TcellP0852 | 15 | GTAAQAAVVRFQEAA |
| TcellP0853 | 20 | GTDTGQACQIQMSDPAYNIN |
| TcellP0854 | 25 | GTGGGNPADEEAAQMGLLGTSPLSN |
| TcellP0855 | 20 | GTTDNFQRYLQAASNGAWGK |
| TcellP0856 | 15 | GTVLVNLINTKLTVA |
| TcellP0857 | 15 | GTVVAVGPGRWDEDG |
| TcellP0858 | 16 | GTVVAVGPGRWDEDGE |
| TcellP0859 | 15 | GTVVLTATFALGAAL |
| TcellP0860 | 16 | GVAVIKAGAATEVELK |
| TcellP0861 | 15 | GVIFFFNPGELLPEA |
| TcellP0862 | 20 | GVITVEESNTFGLQLELTEG |
| TcellP0863 | 30 | GVITVEESNTFGLQLELTEGMRFDKGYISG |
| TcellP0864 | 15 | GVLVATNFFGINTIP |
| TcellP0865 | 9 | GVNAPIPGI |
| TcellP0866 | 16 | GVSTANATVYMIDSVL |
| TcellP0867 | 19 | GVSTANATVYMIDSVLMPP |
| TcellP0868 | 20 | GVSTANATVYMIDSVLMPPA |
| TcellP0869 | 15 | GWIISNIFGAIPVLA |
| TcellP0870 | 15 | GWIISNIFGAIPVLG |
| TcellP0871 | 15 | GWSSLGREYAAVAEE |
| TcellP0872 | 15 | GWYLVAATAAAATLR |
| TcellP0873 | 9 | GYAGTLQSL |
| TcellP0874 | 9 | GYASVIAEL |
| TcellP0875 | 9 | GYETVITQL |
| TcellP0876 | 20 | GYKAADMWGPSSDPAWERND |
| TcellP0877 | 9 | GYLGARRGV |
| TcellP0878 | 20 | GYTSGTGQGNASATKDGSHY |
| TcellP0879 | 9 | HAMSSTHEA |
| TcellP0880 | 15 | HAQTVEDEARRMWAS |
| TcellP0881 | 23 | HAVYRTMMMHLLRLARSFGVLPV |
| TcellP0882 | 11 | HERYPNVTITA |
| TcellP0883 | 20 | HEVTNDDQGDQGDVQPAEVV |
| TcellP0884 | 10 | HFQPLPPAVV |
| TcellP0885 | 20 | HITQAVLTATNFFGINTIPI |
| TcellP0886 | 9 | HLDDVGFLV |
| TcellP0887 | 13 | HLVRGAEVVDTVP |
| TcellP0888 | 9 | HMAQTLGSL |
| TcellP0889 | 15 | HNDVVTVASAPKLRV |
| TcellP0890 | 15 | HPARRAILIEDLLTH |
| TcellP0891 | 15 | HPQQFIYAGSLSALL |
| TcellP0892 | 20 | HPTTTYKAFDWDQAYRKPIT |
| TcellP0893 | 20 | HQAAAVGQAGASAFARQVGL |
| TcellP0894 | 15 | HRDDRYCYFFIPSRK |
| TcellP0895 | 15 | HSLLDEGKQSLTKLA |
| TcellP0896 | 18 | HSWEYWGAQLNAMKGDLQ |
| TcellP0897 | 25 | HTLQQDVINMVNDPFQTLTGRPLIG |
| TcellP0898 | 15 | HTVLVATNFFGINTI |
| TcellP0899 | 16 | HVTLYLGNGQMELPNK |
| TcellP0900 | 25 | HVTPSAVSQRIKSLEQQVGQVLVVR |
| TcellP0901 | 20 | HVVAGQTSPANVVGTRQTLQ |
| TcellP0902 | 9 | HVYAHQAQT |
| TcellP0903 | 15 | HVYLDTVVLLGALAN |
| TcellP0904 | 10 | IAALNPGVNL |
| TcellP0905 | 20 | IAALNPGVNLPGTAVVPLHR |
| TcellP0906 | 9 | IADAALAAL |
| TcellP0907 | 20 | IADAALAALGLWLSVAAVAG |
| TcellP0908 | 20 | IAENRAELMILIATNLLGQN |
| TcellP0909 | 15 | IAFNSGLEPGVVAEK |
| TcellP0910 | 15 | IAGLFLTTEAVVADK |
| TcellP0911 | 15 | IAGMRLLVIKPEPLA |
| TcellP0912 | 20 | IAIGGAATGIAAVLTDGNPP |
| TcellP0913 | 16 | IAKEIELEDPYEKIGA |
| TcellP0914 | 21 | IALAISAQQVNYNLPGVSEHL |
| TcellP0915 | 20 | IALIVITFVVVPAMAYLHYY |
| TcellP0916 | 23 | IALTEMDYFIRMWNQAALAMEVY |
| TcellP0917 | 20 | IANNTRVWVYCGNGKPSDLG |
| TcellP0918 | 15 | IAYPVLWRHLTAIVA |
| TcellP0919 | 15 | ICVRVAEQLAELSSE |
| TcellP0920 | 20 | IDELKTNSSLLTSILTYHVV |
| TcellP0921 | 15 | IDLNVLLSAAINFFL |
| TcellP0922 | 16 | IDQVESTAGSLQGQWR |
| TcellP0923 | 9 | IDSVLMPPA |
| TcellP0924 | 23 | IDYAVNVKGVVVVVAAGNTGGDC |
| TcellP0925 | 15 | IEAAASAIQGNVTSI |
| TcellP0926 | 20 | IEAAASAIQGNVTSIHSLLD |
| TcellP0927 | 15 | IEDAVRNAKAAVEEG |
| TcellP0928 | 9 | IEPRGAQAL |
| TcellP0929 | 16 | IGLSMAGSSAMILAAY |
| TcellP0930 | 15 | IGLVLIAVLVFVPRV |
| TcellP0931 | 15 | IGLVTQTINDFYFVI |
| TcellP0932 | 15 | IGRNFYRALLAQQEQ |
| TcellP0933 | 15 | IGRWGVHQEAMMNLA |
| TcellP0934 | 15 | IGSSIGAANAAAAGS |
| TcellP0935 | 15 | IHSLLDEGKQSLTKL |
| TcellP0936 | 16 | IHSLLDEGKQSLTKLA |
| TcellP0937 | 20 | IHVVFRSDESGTTDNFQRYL |
| TcellP0938 | 15 | IIPEYLFIQSSTDLR |
| TcellP0939 | 15 | IISDVADIIKGTLGE |
| TcellP0940 | 20 | IKGKLNEFVDAVSSTQDQIT |
| TcellP0941 | 20 | IKIFMLVTAVVLLCCSGVAT |
| TcellP0942 | 18 | ILAAYHPQQFIYAGSLSA |
| TcellP0943 | 15 | ILAKYVQLDFFRHVD |
| TcellP0944 | 15 | ILPIAEMSVVAMEFG |
| TcellP0945 | 9 | ILPSDAPVL |
| TcellP0946 | 20 | ILTVSVAVSEGKPTEKHIQI |
| TcellP0947 | 9 | IMANRAQVL |
| TcellP0948 | 10 | IMILLVLVSA |
| TcellP0949 | 15 | IMLLAYYIAAVNIES |
| TcellP0950 | 8 | IMYNYPAM |
| TcellP0951 | 9 | IMYNYPAML |
| TcellP0952 | 21 | INFNCEVWSNVSETISGPRLY |
| TcellP0953 | 15 | INLIIHYVDRPGALG |
| TcellP0954 | 15 | INLIIHYVHRAGALG |
| TcellP0955 | 20 | INSARMYAGPGSASLVAAAK |
| TcellP0956 | 8 | INYEYAIV |
| TcellP0957 | 8 | INYLVPFL |
| TcellP0958 | 9 | IPAEFLENF |
| TcellP0959 | 9 | IPKLRQGSY |
| TcellP0960 | 9 | IPKLVANNT |
| TcellP0961 | 20 | IPPRGTQAVVLKVYQNAGGT |
| TcellP0962 | 9 | IPRDEVRVM |
| TcellP0963 | 15 | IPVMAYLVGLFAWVL |
| TcellP0964 | 24 | IPVVSVTKSVGFQLRGQSGPTTVK |
| TcellP0965 | 15 | IQARAAALAFEQAYA |
| TcellP0966 | 15 | IQGNVTSIHSLLDEG |
| TcellP0967 | 20 | IQMSDPAYNINISLPSYYPD |
| TcellP0968 | 20 | IRAVGPYAWAGRCGRIGRWG |
| TcellP0969 | 20 | IRGSVTPAVSQFNARTADGI |
| TcellP0970 | 20 | IRLHTLLAVLTAAPLLLAAA |
| TcellP0971 | 10 | IRQAGVQYSR |
| TcellP0972 | 15 | IRQAGVQYSRADEEQ |
| TcellP0973 | 18 | IRQAGVQYSRADEEQQQA |
| TcellP0974 | 25 | IRQAGVQYSRADEEQQQALSSQMGF |
| TcellP0975 | 15 | IRQLERLLQAVVGAG |
| TcellP0976 | 18 | IRRAYAEMVATSHEIDDD |
| TcellP0977 | 20 | IRVRNASWQHDIDSLFCTQR |
| TcellP0978 | 15 | ISEAGQAMASTEGNV |
| TcellP0979 | 20 | ISEAGQAMASTEGNVTGMFA |
| TcellP0980 | 20 | ISFLDQASQRGLGEAQLGNS |
| TcellP0981 | 20 | ISGDLKTQIDQVESTAGSLQ |
| TcellP0982 | 20 | ISLPSYYPDQKSLENYIAQT |
| TcellP0983 | 20 | ISPHLSPLSNIVSMLNNHVS |
| TcellP0984 | 15 | ISTNIRQAGVQYSRA |
| TcellP0985 | 15 | ITPDEGAYAFEALLR |
| TcellP0986 | 20 | ITVGLSRGWSLIKSVRLGNA |
| TcellP0987 | 20 | ITVPARWVVNGIERSGEVNA |
| TcellP0988 | 16 | ITYQGWQTQWNQALED |
| TcellP0989 | 9 | IVFATAARY |
| TcellP0990 | 16 | IVKVALEAPLKQIAFN |
| TcellP0991 | 9 | IVLVRRWPK |
| TcellP0992 | 15 | IVQINGRHFDLRAQG |
| TcellP0993 | 20 | IVSMLNNHVSMTNSGVSMAS |
| TcellP0994 | 15 | IVVMYLLLAATAVAA |
| TcellP0995 | 15 | IWHPRKVQSATIYQV |
| TcellP0996 | 9 | IYAGSLSAL |
| TcellP0997 | 15 | IYEQANAHGQKVQAA |
| TcellP0998 | 10 | IYHPQQFVYA |
| TcellP0999 | 10 | IYSKYGGTEI |
| TcellP1000 | 10 | IYYVDANASI |
| TcellP1001 | 15 | KAAIELIADHQLTVL |
| TcellP1002 | 15 | KADDGLAGVIDKIFG |
| TcellP1003 | 15 | KAGLDRLRSVVHSLI |
| TcellP1004 | 18 | KALAAATPMVVWLQTAST |
| TcellP1005 | 15 | KASVPGGGDMGGMDF |
| TcellP1006 | 15 | KDFDGRSEFAYGSFV |
| TcellP1007 | 15 | KDLQDIIAILGIDEL |
| TcellP1008 | 11 | KDTQGADGPAG |
| TcellP1009 | 15 | KEDPAWQRNDPLLNV |
| TcellP1010 | 18 | KFSDPSKPNGQIWTGVIG |
| TcellP1011 | 25 | KGYISGYFVTDPERQEAVLEDPYIL |
| TcellP1012 | 25 | KHSSQLKHAAWVINGLANAYNDTRR |
| TcellP1013 | 16 | KILVQANEAETTTASG |
| TcellP1014 | 20 | KITGTATGVDMANPMSPVNK |
| TcellP1015 | 15 | KKVAGLIKLQIVAGQ |
| TcellP1016 | 9 | KLAGGVAVI |
| TcellP1017 | 25 | KLFDDWSNLGSISELSTSRVLDPAA |
| TcellP1018 | 9 | KLIANNTRV |
| TcellP1019 | 16 | KLNGKVLAAMYQGTIK |
| TcellP1020 | 25 | KLNPDVNLVDTLNGGEYTVFAPTNA |
| TcellP1021 | 9 | KLQERLAKL |
| TcellP1022 | 9 | KLVANNTRL |
| TcellP1023 | 18 | KLVANNTRLWVYCGNGTP |
| TcellP1024 | 15 | KMAPVLRQIYDQMAE |
| TcellP1025 | 25 | KPDAEEQGVPVSPTASDPALLAEIR |
| TcellP1026 | 21 | KPDLQRALGATPNTGPAPQGA |
| TcellP1027 | 15 | KPEKEKASVPGGGDM |
| TcellP1028 | 9 | KPGCDAPAY |
| TcellP1029 | 20 | KPGTKSGDRVGIWVDSAGQL |
| TcellP1030 | 9 | KPLLIIAED |
| TcellP1031 | 15 | KPLVLILDDFAMREH |
| TcellP1032 | 9 | KPRDDAAAL |
| TcellP1033 | 20 | KPRIITLTMNPALDITTSVD |
| TcellP1034 | 15 | KPTGSAAIGLSMAGS |
| TcellP1035 | 8 | KPTGSAVV |
| TcellP1036 | 20 | KQDPEGWGKSPGFGTTVDFP |
| TcellP1037 | 15 | KQELDEISTNIRQAG |
| TcellP1038 | 15 | KQQVIAELYEKFFRI |
| TcellP1039 | 15 | KQSLTKLAAAWGGSG |
| TcellP1040 | 16 | KQSLTKLAAAWGGSGS |
| TcellP1041 | 22 | KRGIEKAVEKVTETLLKDAKEV |
| TcellP1042 | 16 | KRIPLDVAEGDTVIYS |
| TcellP1043 | 20 | KSLENYIAQTRDKFLSAATS |
| TcellP1044 | 30 | KSLENYIAQTRDKFLSAATSSTPREAPYEL |
| TcellP1045 | 11 | KTIAYDEEARR |
| TcellP1046 | 15 | KTQIDQVESTAGSLQ |
| TcellP1047 | 15 | KVALEAPLKQIAFNS |
| TcellP1048 | 25 | KVDSLLGITSADVDVRANPLAAKGV |
| TcellP1049 | 15 | KVQAAGNNMAQTDSA |
| TcellP1050 | 15 | KVQFQSGGANSPALY |
| TcellP1051 | 15 | KVQSATIYQVTDRSH |
| TcellP1052 | 11 | KVVIDGKDQNV |
| TcellP1053 | 20 | KVVIDGKDQNVTGSVVCTTA |
| TcellP1054 | 9 | KWDATATEL |
| TcellP1055 | 16 | KWDATATELNNALQNL |
| TcellP1056 | 15 | KWETLLTSELPQWLS |
| TcellP1057 | 16 | KYGGTEIKYNGEEYLI |
| TcellP1058 | 9 | KYIAADRKI |
| TcellP1059 | 9 | KYIFPGGLL |
| TcellP1060 | 15 | LAAAAAWDALAAELY |
| TcellP1061 | 20 | LAAAGAYLKADDGLAGVIDK |
| TcellP1062 | 9 | LAGKGISVV |
| TcellP1063 | 20 | LAIYHPQQFVYAGAMSGLLD |
| TcellP1064 | 20 | LAKIFNGSITQWNNPAIQAL |
| TcellP1065 | 16 | LAKLAGGVAVIKAGAA |
| TcellP1066 | 15 | LALLLVPGVPLVVMP |
| TcellP1067 | 15 | LALVGFLGGLITGIS |
| TcellP1068 | 15 | LALVGFLGGLITGTS |
| TcellP1069 | 15 | LAMGDAGGYKAADMW |
| TcellP1070 | 25 | LANAYNDTRRKVVPPEEIAANREER |
| TcellP1071 | 15 | LAQEAGNFERISGDL |
| TcellP1072 | 20 | LAQEAGNFERISGDLKTQID |
| TcellP1073 | 20 | LAQPTKSIWPFDQLSELWKA |
| TcellP1074 | 15 | LARTISEAGQAMAST |
| TcellP1075 | 24 | LARTISEAGQAMASTEGNVTGMFA |
| TcellP1076 | 15 | LAVRYGISSLEEAQA |
| TcellP1077 | 15 | LAWLVQASANSAAMA |
| TcellP1078 | 16 | LDEGKQSLTKLAAAWG |
| TcellP1079 | 15 | LDEISTNIRQAGVQY |
| TcellP1080 | 19 | LDEISTNIRQAGVQYSRAD |
| TcellP1081 | 15 | LDFGALPPEINSGRM |
| TcellP1082 | 18 | LDLHTLKLRPHAPADRIT |
| TcellP1083 | 15 | LDPGASQSTTNPIFG |
| TcellP1084 | 15 | LDVAEGDTVIYSKYG |
| TcellP1085 | 15 | LDWFCLFSSAAALTG |
| TcellP1086 | 15 | LDYLRRMTVFLQGLM |
| TcellP1087 | 20 | LEAVVMLLAVTVSLLTIPFA |
| TcellP1088 | 16 | LEDKILVQANEAETTT |
| TcellP1089 | 15 | LEDPYEKIGAELVKE |
| TcellP1090 | 16 | LEDPYEKIGAELVKEV |
| TcellP1091 | 9 | LEEIGILLL |
| TcellP1092 | 15 | LENDNQLLYNYPGAL |
| TcellP1093 | 11 | LEPGVVAEKVR |
| TcellP1094 | 16 | LEPGVVAEKVRNLPAG |
| TcellP1095 | 18 | LEPMASILDPGASQSTTN |
| TcellP1096 | 9 | LEVPAMGVL |
| TcellP1097 | 9 | LFAAFPSFA |
| TcellP1098 | 15 | LFAAFPSFAGLRPTF |
| TcellP1099 | 20 | LFAAFPSFAGLRPTFDTRLM |
| TcellP1100 | 25 | LFEKLEPMASILDPGASQSTTNPIF |
| TcellP1101 | 20 | LFNLWGPAFHERYPNVTITA |
| TcellP1102 | 25 | LGACLALWLSGCSSPKPDAEEQGVP |
| TcellP1103 | 15 | LGALTIVPRLVAHPT |
| TcellP1104 | 15 | LGDIVYVHGAVISSR |
| TcellP1105 | 20 | LGGCSTALFPAGGSTGSLLM |
| TcellP1106 | 15 | LGGLWTAVSPHLSPL |
| TcellP1107 | 20 | LGGNNLPAKFLEGFVRTSNI |
| TcellP1108 | 9 | LGNVNGVTL |
| TcellP1109 | 20 | LGRWKWHDPWVHASLLAQNN |
| TcellP1110 | 9 | LIASNVAGV |
| TcellP1111 | 20 | LIATNLLGQNTPAIAVNEAE |
| TcellP1112 | 20 | LIDRGRAEVVVVSLGSQGAL |
| TcellP1113 | 15 | LIESGVLIPARRPQN |
| TcellP1114 | 18 | LIGLAMGDAGGYKAADMW |
| TcellP1115 | 15 | LIILRKRENFRRAFS |
| TcellP1116 | 20 | LIKSVRLGNAAGAAMLLTPG |
| TcellP1117 | 9 | LILATMLVA |
| TcellP1118 | 15 | LKTQIDQVESTAGSL |
| TcellP1119 | 20 | LKVYQNAGGTHPTTTYKAFD |
| TcellP1120 | 10 | LLAVLTAAPL |
| TcellP1121 | 9 | LLDAHIPQL |
| TcellP1122 | 9 | LLDEGKQSL |
| TcellP1123 | 9 | LLDGLLAWY |
| TcellP1124 | 15 | LLDGLRAQDDFSGWD |
| TcellP1125 | 18 | LLDPSQGMGLIGLAMGDA |
| TcellP1126 | 25 | LLDVRIEDQDHSARLLREGVAMGAV |
| TcellP1127 | 15 | LLEFAVVLELAILSI |
| TcellP1128 | 20 | LLEQAVAVEEAIDTAAANQL |
| TcellP1129 | 15 | LLGQNTAAIAAIEAQ |
| TcellP1130 | 15 | LLPLLEKVIGAGKPL |
| TcellP1131 | 15 | LLQAAPTLDELKLEG |
| TcellP1132 | 18 | LLRAESLPGAGGSLTRTP |
| TcellP1133 | 15 | LLSTRGYITAEKIRS |
| TcellP1134 | 10 | LLSVLAAVGL |
| TcellP1135 | 18 | LLTSELPQWLSANRAVKP |
| TcellP1136 | 15 | LLVIPVALSASIIRL |
| TcellP1137 | 15 | LLVSSKVSTVKDLLP |
| TcellP1138 | 9 | LLYDGSFAV |
| TcellP1139 | 9 | LMHYRGELL |
| TcellP1140 | 9 | LMIGTAAAV |
| TcellP1141 | 9 | LMLADHPEY |
| TcellP1142 | 9 | LMVVRALFL |
| TcellP1143 | 15 | LNAMKGDLQSSLGAG |
| TcellP1144 | 23 | LNIYVRRWRTALHATVSALIVAI |
| TcellP1145 | 15 | LNNALQNLARTISEA |
| TcellP1146 | 16 | LNNALQNLARTISEAG |
| TcellP1147 | 19 | LNNALQNLARTISEAGQAM |
| TcellP1148 | 20 | LNPQVNLVDTLNSGQYTVFA |
| TcellP1149 | 20 | LNSGQYTVFAPTNAAFSKLP |
| TcellP1150 | 25 | LNSGQYTVFAPTNAAFSKLPASTID |
| TcellP1151 | 15 | LNYRPLLPKDRRMII |
| TcellP1152 | 16 | LPAGHGLNAQTGVYED |
| TcellP1153 | 18 | LPEIAANHITQAVLTATN |
| TcellP1154 | 25 | LPGAGGSLTRTPLMSQLIEKPVAPS |
| TcellP1155 | 9 | LPGTAVVPL |
| TcellP1156 | 20 | LPIARPTIALAAQAFRDEIV |
| TcellP1157 | 20 | LPLGQLTNSGGGFGGVSNAL |
| TcellP1158 | 15 | LPLLVPLRAIPLLGN |
| TcellP1159 | 15 | LPPEINSGRMYCGPG |
| TcellP1160 | 20 | LPPGVAADYYQRVADICRRS |
| TcellP1161 | 9 | LPQWLSANR |
| TcellP1162 | 25 | LPVASRPFVQRHLSDGFTAAAAAKA |
| TcellP1163 | 9 | LPVEYLQVP |
| TcellP1164 | 15 | LPVEYLQVPSPSMGR |
| TcellP1165 | 15 | LQGQWRGAAGTAAQA |
| TcellP1166 | 15 | LQNAASIAGLFLTTE |
| TcellP1167 | 8 | LQNLARTI |
| TcellP1168 | 15 | LQNLARTISEAGQAM |
| TcellP1169 | 23 | LQNLARTISEAGQAMASTEGNVT |
| TcellP1170 | 15 | LQSLGADIASEQAVL |
| TcellP1171 | 16 | LQSLGADIASEQAVLS |
| TcellP1172 | 15 | LQSLGAEIAVEQAAL |
| TcellP1173 | 16 | LQSLGAEIAVEQAALQ |
| TcellP1174 | 15 | LQSLWANFYELLADA |
| TcellP1175 | 15 | LQVPSPSMGRDIKVQ |
| TcellP1176 | 20 | LQVPSPSMGRDIKVQFQSGG |
| TcellP1177 | 15 | LQVVLRGYASMVAEL |
| TcellP1178 | 19 | LREAAEKAKIELSSSQSTS |
| TcellP1179 | 25 | LREGVAMGAVTTERNPVPGCRVHPL |
| TcellP1180 | 15 | LRGLLSTFIAALMGA |
| TcellP1181 | 15 | LRIAAKIYSEADEAW |
| TcellP1182 | 15 | LRLLVIALKHNVILN |
| TcellP1183 | 10 | LRPTFDTRLM |
| TcellP1184 | 20 | LRPTFDTRLMRLEDEMKEGR |
| TcellP1185 | 22 | LRPTFDTRLMRLEDEMKEGRYE |
| TcellP1186 | 15 | LRQHASRYLARVEAG |
| TcellP1187 | 18 | LSANRAVKPTGSAAIGLS |
| TcellP1188 | 18 | LSANRAVKPTGSPSAAIG |
| TcellP1189 | 12 | LSFTLTGNPNRP |
| TcellP1190 | 25 | LSGVTNLQAQGTEVIDGISTTKITG |
| TcellP1191 | 15 | LSIVMPVGGQSSFYS |
| TcellP1192 | 18 | LSMAGSSAMILAAYHPQQ |
| TcellP1193 | 15 | LSPISNMVSMANNHM |
| TcellP1194 | 15 | LSPISNMVSMANNHV |
| TcellP1195 | 15 | LSPLSNMVSMANNHM |
| TcellP1196 | 20 | LSVPQAWAAANQAVTPAARA |
| TcellP1197 | 20 | LTAAAQYMREHDIGALPICG |
| TcellP1198 | 16 | LTAAPLLLAAAGCGSK |
| TcellP1199 | 15 | LTAIRYQIVVMYLLL |
| TcellP1200 | 15 | LTATNFFGINTIPIA |
| TcellP1201 | 25 | LTFLGFIGVLLAINLFGNRAIKWAN |
| TcellP1202 | 15 | LTKLAAAWGGSGSEA |
| TcellP1203 | 15 | LTLNEIHAFIKDPLG |
| TcellP1204 | 9 | LTRAILIRV |
| TcellP1205 | 20 | LTREMPAWLQANKGVSPTGN |
| TcellP1206 | 20 | LTSELPGWLQANRHVKPTGS |
| TcellP1207 | 15 | LTSELPQWLSANRAV |
| TcellP1208 | 20 | LTSELPQWLSANRAVKPTGS |
| TcellP1209 | 15 | LTVDAGAYASAEAAN |
| TcellP1210 | 15 | LTVDAGAYVSAEAAN |
| TcellP1211 | 15 | LVAQEAAAAGTPLVT |
| TcellP1212 | 25 | LVCGGVHTANATVYMIDTVLMPPAQ |
| TcellP1213 | 15 | LVILLIIYRNPITMV |
| TcellP1214 | 20 | LVLDTDSFYRPKRPGSYPIV |
| TcellP1215 | 15 | LVLVIDDEPQILRAL |
| TcellP1216 | 13 | LVPVNHLPLTLPL |
| TcellP1217 | 18 | LVRAVAESHGVAAVLFAA |
| TcellP1218 | 9 | LVRAYHAMS |
| TcellP1219 | 9 | LVVADLSFI |
| TcellP1220 | 16 | LWFELMKPMTSTATGR |
| TcellP1221 | 15 | LWIWVALTGAAATVL |
| TcellP1222 | 20 | LWLSVAAVAGALLALTRAIL |
| TcellP1223 | 15 | LYRPGLVHIYHALTW |
| TcellP1224 | 20 | MAAHKGLMNIALAISAQQVN |
| TcellP1225 | 25 | MAAIATFAAPVALAAYPITGKLGSE |
| TcellP1226 | 15 | MAEAWFETVAIAQQR |
| TcellP1227 | 15 | MAEMKTDAATLAQEA |
| TcellP1228 | 18 | MAEMKTDAATLAQEAGNF |
| TcellP1229 | 20 | MAEMKTDAATLAQEAGNFER |
| TcellP1230 | 25 | MAEMKTDAATLAQEAGNFERISGDL |
| TcellP1231 | 15 | MAFLRSVSCLAAAVF |
| TcellP1232 | 15 | MAFLRSVSRLAAAVF |
| TcellP1233 | 9 | MAKTIAYDE |
| TcellP1234 | 13 | MAKTIAYDEEARR |
| TcellP1235 | 15 | MAKTIAYDEEARRGL |
| TcellP1236 | 16 | MAKTIAYDEEARRGLE |
| TcellP1237 | 20 | MAKTIAYDEEARRGLERGLN |
| TcellP1238 | 16 | MAMLARDGAEAAKWGG |
| TcellP1239 | 9 | MAMMARDTA |
| TcellP1240 | 16 | MAMMARDTAEAAKWGG |
| TcellP1241 | 15 | MAMTINYQFGDVDAH |
| TcellP1242 | 11 | MANPFVKAWKY |
| TcellP1243 | 19 | MANPMSPVNKSFEIEVTCS |
| TcellP1244 | 15 | MAQDPVATAASNNPM |
| TcellP1245 | 9 | MARAVGIDL |
| TcellP1246 | 20 | MASAAAFETAFALTVPPAEV |
| TcellP1247 | 11 | MATTLPVQRHP |
| TcellP1248 | 20 | MATTLPVQRHPRSLFPEFSE |
| TcellP1249 | 25 | MATWFSAVFDGLGDVLLDVRIEDQD |
| TcellP1250 | 15 | MDFGLLPPEVNSSRM |
| TcellP1251 | 15 | MDYFIRMWNQAALAM |
| TcellP1252 | 18 | MDYFIRMWNQAALAMEVY |
| TcellP1253 | 6 | MFFLDA |
| TcellP1254 | 6 | MFLIDV |
| TcellP1255 | 15 | MFVLRSHAAGLLREG |
| TcellP1256 | 15 | MGRDIKVQFQSGGAN |
| TcellP1257 | 25 | MHPMIPAEYISNIIYEGPGADSLFF |
| TcellP1258 | 23 | MHRIFLITVALALLTASPASAIT |
| TcellP1259 | 15 | MHVSFVMAYPEMLAA |
| TcellP1260 | 20 | MIATTRDREGATMITFRLRL |
| TcellP1261 | 15 | MIGTAAAVVLPGLVG |
| TcellP1262 | 5 | MILLV |
| TcellP1263 | 15 | MINVQAKPAAAASLA |
| TcellP1264 | 25 | MINVQAKPAAAASLAAIAIAFLAGC |
| TcellP1265 | 15 | MIRAQAGLLEAEHQA |
| TcellP1266 | 6 | MIVVLV |
| TcellP1267 | 15 | MKEGRYEVRAELPGV |
| TcellP1268 | 16 | MKIRLHTLLAVLTAAP |
| TcellP1269 | 20 | MKIRLHTLLAVLTAAPLLLA |
| TcellP1270 | 18 | MKLGWHPYHFPDEPDSKQ |
| TcellP1271 | 25 | MKLTTMIKTAVAVVAMAAIATFAAP |
| TcellP1272 | 22 | MKPDLQRALGATPNTGPAPQGA |
| TcellP1273 | 20 | MKRGLTVAVAGAAILVAGLS |
| TcellP1274 | 24 | MKRGLTVAVAGAAILVAGLSGCSS |
| TcellP1275 | 20 | MKVKNTIAATSFAAAGLAAL |
| TcellP1276 | 25 | MKVKNTIAATSFAAAGLAALAVAVS |
| TcellP1277 | 9 | MLGHAGDMA |
| TcellP1278 | 18 | MLGHAGDMAGYAGTLQSL |
| TcellP1279 | 16 | MLGNAPSVVPNTTLGM |
| TcellP1280 | 16 | MLGTGTPNRARINFNC |
| TcellP1281 | 25 | MLLRKGTVYVLVIRADLVNAMVAHA |
| TcellP1282 | 6 | MLVLLV |
| TcellP1283 | 19 | MLVLLVAVLVTAVYAFVHA |
| TcellP1284 | 18 | MLWHAMPPELNTARLMAG |
| TcellP1285 | 15 | MMAHAGDMAGYAGTL |
| TcellP1286 | 20 | MMAHAGDMAGYAGTLQSLGA |
| TcellP1287 | 9 | MMARDTAEA |
| TcellP1288 | 9 | MMKYLAFGL |
| TcellP1289 | 25 | MNDGKRAVTSAVLVVLGACLALWLS |
| TcellP1290 | 20 | MNNVPQALQQLAQPTKSIWP |
| TcellP1291 | 19 | MNRHSTAASDRGLQAERTT |
| TcellP1292 | 9 | MPVGGGSSF |
| TcellP1293 | 9 | MPVGGQSSF |
| TcellP1294 | 9 | MQLVDRVRG |
| TcellP1295 | 11 | MQLVDRVRGAV |
| TcellP1296 | 32 | MRFAQPSALSRFSALTRDWFTSTFAAPTAAQA |
| TcellP1297 | 20 | MRIKIFMLVTAVVLLCCSGV |
| TcellP1298 | 19 | MRILPISTIKGKLNEFVDA |
| TcellP1299 | 15 | MRNVRLFRALLGVDK |
| TcellP1300 | 15 | MSFVTTQPEALAAAA |
| TcellP1301 | 18 | MSGHALAARTLLAAADEL |
| TcellP1302 | 15 | MSPVNKSFEIEVTCS |
| TcellP1303 | 18 | MSQIMYNYPAMLGHAGDM |
| TcellP1304 | 15 | MSQIMYNYPAMMAHA |
| TcellP1305 | 16 | MSQIMYNYPAMMAHAG |
| TcellP1306 | 20 | MSQIMYNYPAMMAHAGDMAG |
| TcellP1307 | 15 | MSQIMYNYPAMRAHA |
| TcellP1308 | 20 | MSQSMYSYPAMTANVGDMAG |
| TcellP1309 | 9 | MSRRLVVGA |
| TcellP1310 | 15 | MSTQRPRHSGIRAVG |
| TcellP1311 | 20 | MSTQRPRHSGIRAVGPYAWA |
| TcellP1312 | 20 | MSWVMVSPELVVAAAADLAG |
| TcellP1313 | 19 | MTAISCSPRPRYASRMPVL |
| TcellP1314 | 20 | MTANVGDMAGYTGTTQSLGA |
| TcellP1315 | 15 | MTDVSRKIRAWGRRL |
| TcellP1316 | 40 | MTDVSRKIRAWGRRLMIGTAAAVVLPGLVGLAGGAATAGA |
| TcellP1317 | 20 | MTEPAAWDEGKPRIITLTMN |
| TcellP1318 | 8 | MTEQQWNF |
| TcellP1319 | 15 | MTEQQWNFAGIEAAA |
| TcellP1320 | 16 | MTEQQWNFAGIEAAAS |
| TcellP1321 | 18 | MTEQQWNFAGIEAAASAI |
| TcellP1322 | 20 | MTEQQWNFAGIEAAASAIQG |
| TcellP1323 | 25 | MTEQQWNFAGIEAAASAIQGNVTSI |
| TcellP1324 | 11 | MTFFEQVRRLR |
| TcellP1325 | 19 | MTGFLGVVPSFLKVLAGMH |
| TcellP1326 | 18 | MTHKRTKRQPAIAAGLNA |
| TcellP1327 | 20 | MTNSGVSMASTLHSMLKGFA |
| TcellP1328 | 19 | MTVKRTTIELDEDLVRAAQ |
| TcellP1329 | 9 | MTYAAPLFV |
| TcellP1330 | 21 | MVDFGALPPEINSARMYAGPG |
| TcellP1331 | 25 | MVDPQLDGPQLAALAAVVELGSFDA |
| TcellP1332 | 11 | MVNKSRMMPAV |
| TcellP1333 | 15 | MVQIVQVRVLATELQ |
| TcellP1334 | 15 | MVWQREKLLQVNEIG |
| TcellP1335 | 15 | MWDPDVYLAFSGHRN |
| TcellP1336 | 20 | MWDSVASDLFSAASAFQSVV |
| TcellP1337 | 20 | MYGYAAASAVAARLNPLTRP |
| TcellP1338 | 9 | MYNYPAMLG |
| TcellP1339 | 15 | MYRELLELVAADVES |
| TcellP1340 | 11 | NAAFSKLPAST |
| TcellP1341 | 15 | NAHGQKVQAAGNNMA |
| TcellP1342 | 13 | NALQNLARTISEA |
| TcellP1343 | 15 | NALQNLARTISEAGQ |
| TcellP1344 | 18 | NALQNLARTISEAGQAMA |
| TcellP1345 | 20 | NAMTRFVNVFEQACPGQTLN |
| TcellP1346 | 9 | NAVFNFPPN |
| TcellP1347 | 20 | NAVFNFPPNGTHSWEYWGAQ |
| TcellP1348 | 20 | NDGVIFFFNPGELLPEAAGP |
| TcellP1349 | 20 | NDPLLNVGKLIANNTRVWVY |
| TcellP1350 | 20 | NDPMVQIPRLVANNTRIWVY |
| TcellP1351 | 15 | NDPTQQIPKLVANNT |
| TcellP1352 | 20 | NDPTQQIPKLVANNTRLWVY |
| TcellP1353 | 19 | NEAEYGEMWAQDAAAMFGY |
| TcellP1354 | 18 | NELGGANIPAEFLENFVR |
| TcellP1355 | 9 | NFAGIEAAA |
| TcellP1356 | 15 | NFAGIEAAASAIQGN |
| TcellP1357 | 16 | NFERISGDLKTQIDQV |
| TcellP1358 | 20 | NFLLPDAQSIQAAAAGFASK |
| TcellP1359 | 20 | NFVRSSNLKFQDAYKPAGGH |
| TcellP1360 | 20 | NFVRSSNLKFQDAYNAAGGH |
| TcellP1361 | 8 | NGEEYLIL |
| TcellP1362 | 20 | NGKVLAAMYQGTIKTWDDPQ |
| TcellP1363 | 20 | NGTPNELGGANIPAEFLENF |
| TcellP1364 | 20 | NGVFDFPDSGTHSWEYWGAQ |
| TcellP1365 | 20 | NGVFNFPPNGTHSWPYWNEQ |
| TcellP1366 | 16 | NIALAISAQQVNYNLP |
| TcellP1367 | 9 | NIRQAGVQY |
| TcellP1368 | 15 | NIRQAGVQYSRADEE |
| TcellP1369 | 18 | NIRQAGVQYSRADEEQQQ |
| TcellP1370 | 20 | NITSATYQSAIPPRGTQAVV |
| TcellP1371 | 15 | NLAICLVLIYAIALV |
| TcellP1372 | 15 | NLARTISEAGQAMAS |
| TcellP1373 | 20 | NLARTISEAGQAMASTEGNV |
| TcellP1374 | 9 | NLLVTGFDT |
| TcellP1375 | 9 | NMMARGMDL |
| TcellP1376 | 20 | NMTLLADAMGTVGGAAPGGG |
| TcellP1377 | 15 | NNALQNLARTISEAG |
| TcellP1378 | 15 | NNSPAVYLLDGLRAQ |
| TcellP1379 | 20 | NNSPAVYLLDGLRAQDDYNG |
| TcellP1380 | 20 | NNTRLWVYCGNGTPNELGGA |
| TcellP1381 | 15 | NNTRVWVYCGNGKPS |
| TcellP1382 | 15 | NPFPFLRQIIANQQV |
| TcellP1383 | 15 | NPGLLRFLPQLSERL |
| TcellP1384 | 15 | NPLGLKRGIEKAVEK |
| TcellP1385 | 15 | NQAFRNIVNMLHGVR |
| TcellP1386 | 7 | NQAISMI |
| TcellP1387 | 20 | NQAVTPAARALPLTSLTSAA |
| TcellP1388 | 15 | NQGGWMLSRASAMEL |
| TcellP1389 | 15 | NQRQKFNPLVRLDSI |
| TcellP1390 | 15 | NRASLMQLISTNVFG |
| TcellP1391 | 25 | NRDDGLQDMLVRKAFRRAITRPTHF |
| TcellP1392 | 20 | NSARMYGGAGAADLLAAAAA |
| TcellP1393 | 15 | NSGRMYCGPGSGPML |
| TcellP1394 | 20 | NSLKVGNADVVCGGVSTANA |
| TcellP1395 | 18 | NTIPIALTEMDYFIRMWN |
| TcellP1396 | 9 | NVTSIHSLL |
| TcellP1397 | 15 | NVTSIHSLLDEGKQS |
| TcellP1398 | 16 | NVTSIHSLLDEGKQSL |
| TcellP1399 | 20 | NVTSIHSLLDEGKQSLTKLA |
| TcellP1400 | 15 | NYEQQEQASQQILSS |
| TcellP1401 | 10 | NYEYAIVNNR |
| TcellP1402 | 9 | NYIPQQLAL |
| TcellP1403 | 16 | NYPAMMAHAGDMAGYA |
| TcellP1404 | 25 | PAATIDQLKTDAKLLSSILTYHVIA |
| TcellP1405 | 18 | PADVPSAEQRRAQRQRDL |
| TcellP1406 | 16 | PAFEWYYQSGLSIVMP |
| TcellP1407 | 20 | PAGVGGTALTADLGNASVVG |
| TcellP1408 | 20 | PALDITTSVDVVRPTEKMRC |
| TcellP1409 | 16 | PALDKLKLTGDEATGA |
| TcellP1410 | 15 | PALFVFRPLLNLALR |
| TcellP1411 | 22 | PAVLAVAVVVAFLTTGCIRWST |
| TcellP1412 | 25 | PAVSQFNARTADGINYRVLWQAAGP |
| TcellP1413 | 16 | PAVVKLSDALIATISS |
| TcellP1414 | 15 | PAWERNDPTQQIPKL |
| TcellP1415 | 20 | PCRTILRVFSRNPLVRGTDR |
| TcellP1416 | 20 | PDQKSLENYIAQTRDKFLSA |
| TcellP1417 | 15 | PDTAKEKPQEGTVVA |
| TcellP1418 | 15 | PEFSELFAAFPSFAG |
| TcellP1419 | 25 | PEKLAASPLADGSFVRVCDIHLDVP |
| TcellP1420 | 20 | PGGWVEADEDTFYDRAQEYS |
| TcellP1421 | 13 | PHVKPAALAEQPG |
| TcellP1422 | 20 | PIAVTYNLNSVSSLNLDGPT |
| TcellP1423 | 15 | PLLNVGKLIANNTRV |
| TcellP1424 | 20 | PLPVVFPIVQGELSKQTGQQ |
| TcellP1425 | 9 | PMAPLAPLL |
| TcellP1426 | 9 | PMKRTALAL |
| TcellP1427 | 18 | PMLAAAAGWQTLSAALDA |
| TcellP1428 | 9 | PMQQLTQPL |
| TcellP1429 | 15 | PNGTHSWEYWGAQLN |
| TcellP1430 | 15 | PPEVNSARVFAGAGS |
| TcellP1431 | 15 | PQWLSANRAVKPTGS |
| TcellP1432 | 15 | PRHSGIRAVGPYAWA |
| TcellP1433 | 20 | PRSLFPEFSELFAAFPSFAG |
| TcellP1434 | 23 | PRVVATVPQLVQLAPHAVQMSQN |
| TcellP1435 | 14 | PRYISLIPVNVVAD |
| TcellP1436 | 15 | PSLIGLAMGDAGGYK |
| TcellP1437 | 20 | PSLIGLAMGDAGGYKAADMW |
| TcellP1438 | 20 | PSLTVAEQEQCLDELRGAAA |
| TcellP1439 | 9 | PSMGRDIKV |
| TcellP1440 | 15 | PSMGRDIKVQFQSGG |
| TcellP1441 | 15 | PSPSMGRDIKVQFQS |
| TcellP1442 | 24 | PSWGLVVTMFAWGYLLDHVGERMV |
| TcellP1443 | 25 | PTGPASVQGMSQDPVAVAASNNPEL |
| TcellP1444 | 20 | PTLIGLAMGDAGGYKASDMW |
| TcellP1445 | 9 | PTNAAFDKL |
| TcellP1446 | 20 | PTNAAFSKLPASTIDELKTN |
| TcellP1447 | 20 | PTQQIPKLVANNTRLWVYCG |
| TcellP1448 | 15 | PVEYLQVPSPSMGRD |
| TcellP1449 | 20 | PVGGQSSFYSDWYQPACGKA |
| TcellP1450 | 15 | PVGGQSSFYSDWYSP |
| TcellP1451 | 20 | PVGGQSSFYSDWYSPACGKA |
| TcellP1452 | 20 | PVGGQSSFYTDWYQPSQSNG |
| TcellP1453 | 18 | PVGQLPPAATQTLGQLGE |
| TcellP1454 | 20 | PVNYQNFAVTNDGVIFFFNP |
| TcellP1455 | 25 | PVPGCRVHPLGEMRYLPVASRPFVQ |
| TcellP1456 | 25 | PVTTAAMADPAADLIGRGCAQYAAQ |
| TcellP1457 | 9 | PYEKIGAEL |
| TcellP1458 | 10 | PYILLVSSKV |
| TcellP1459 | 15 | PYILLVSSKVSTVKD |
| TcellP1460 | 20 | PYLVWLTCTAESSALAAAQA |
| TcellP1461 | 9 | PYNLRYRVL |
| TcellP1462 | 15 | QAARGIRPLFDDITE |
| TcellP1463 | 20 | QAASNGAWGKGAGKSFQGGV |
| TcellP1464 | 15 | QAAVVRFQEAANKQK |
| TcellP1465 | 15 | QAFRSRFGENFFYIL |
| TcellP1466 | 15 | QAMASTEGNVTGMFA |
| TcellP1467 | 9 | QAMEDLVRA |
| TcellP1468 | 9 | QAQTRHPAT |
| TcellP1469 | 15 | QASPDLLRGLLSTFI |
| TcellP1470 | 25 | QATAQAAAYTQAMATTPSLPEIAAN |
| TcellP1471 | 15 | QAVLSSAWQGDTGIT |
| TcellP1472 | 15 | QAVLTATNFFGINTI |
| TcellP1473 | 15 | QAYLALRAWGLPVSE |
| TcellP1474 | 15 | QCKDIIDELERMQVF |
| TcellP1475 | 15 | QDAYKPAGGHNAVFN |
| TcellP1476 | 20 | QDAYKPAGGHNAVFNFPPNG |
| TcellP1477 | 15 | QDAYNAAGGHNAVFN |
| TcellP1478 | 16 | QDAYNAAGGHNAVFNF |
| TcellP1479 | 20 | QDAYNAAGGHNAVFNFPPNG |
| TcellP1480 | 15 | QDAYNAGGGHNGVFD |
| TcellP1481 | 20 | QDAYNAGGGHNGVFDFPDSG |
| TcellP1482 | 16 | QEAGNFERISGDLKTQ |
| TcellP1483 | 20 | QETLYWLAQPGIRESIAEAD |
| TcellP1484 | 15 | QFGDVDAHGAMIRAQ |
| TcellP1485 | 19 | QFIYAGSLSALLDPSQGMG |
| TcellP1486 | 9 | QFLNLDVPL |
| TcellP1487 | 20 | QFLNLDVPLFVESAFHGLGG |
| TcellP1488 | 15 | QGGLAPVMMQQTFST |
| TcellP1489 | 15 | QGNVTSIHSLLDEGK |
| TcellP1490 | 15 | QGQWRGAAGTAAQAA |
| TcellP1491 | 20 | QGTGSGAGIAQAAAGTVNIG |
| TcellP1492 | 24 | QGVQQKWDATATELNNALQNLART |
| TcellP1493 | 9 | QIMYNYPAM |
| TcellP1494 | 15 | QIPKLVANNTRLWVY |
| TcellP1495 | 20 | QIPQYKHSVVMGVNKAKVPG |
| TcellP1496 | 20 | QKQELDEISTNIRQAGVQYS |
| TcellP1497 | 15 | QKWDATATELNNALQ |
| TcellP1498 | 25 | QLIEKPVAPSVMPAAAAGSSATGGA |
| TcellP1499 | 15 | QLSAEYASTAAELSG |
| TcellP1500 | 15 | QLSALWARFPLPVIP |
| TcellP1501 | 20 | QLTHGSFTSKFNDTLQEFET |
| TcellP1502 | 15 | QMATTLPVQRHPRSL |
| TcellP1503 | 9 | QMKFYAVAL |
| TcellP1504 | 15 | QMPYQPVQSPTQVEA |
| TcellP1505 | 9 | QMRACARRL |
| TcellP1506 | 20 | QMSDPAYNINISLPSYYPDQ |
| TcellP1507 | 15 | QNLARTISEAGQAMA |
| TcellP1508 | 20 | QNYTYKWETFLTREMPAWLQ |
| TcellP1509 | 20 | QPGIRESIAEADADIASGRT |
| TcellP1510 | 13 | QQFIYAGSLSALL |
| TcellP1511 | 15 | QQFIYAGSLSALLDP |
| TcellP1512 | 19 | QQFIYAGSLSALLDPSQGM |
| TcellP1513 | 20 | QQFIYAGSLSALLDPSQGMG |
| TcellP1514 | 20 | QQFPYAASLSGFLNPSEGWW |
| TcellP1515 | 20 | QQFVYAGAMSGLLDPSQAMG |
| TcellP1516 | 15 | QQIKFAALSARAVAL |
| TcellP1517 | 15 | QQKWDATATELNNAL |
| TcellP1518 | 18 | QQLVAAAAEQAAARRRRI |
| TcellP1519 | 25 | QQVGQVLVVREKPCRATTAGIPLLR |
| TcellP1520 | 18 | QQVTSLFSQVGGTGGGNP |
| TcellP1521 | 10 | QQWNFAGIEA |
| TcellP1522 | 25 | QQWNFAGIEAAASAIQGNVTSIHSL |
| TcellP1523 | 15 | QRCRVHFMRNLYTAV |
| TcellP1524 | 20 | QRNDPLLNVGKLIANNTRVW |
| TcellP1525 | 15 | QRPRMLYDYFHQLFA |
| TcellP1526 | 20 | QRVADICRRSSTPLILDTSG |
| TcellP1527 | 19 | QSIGDLIAEAMDKVGNEGV |
| TcellP1528 | 9 | QSSFYSDWY |
| TcellP1529 | 20 | QTAPGHMLGGLPLGQLTNSG |
| TcellP1530 | 8 | QTGVYEDL |
| TcellP1531 | 18 | QTLGQLGEMSGPMQQLTQ |
| TcellP1532 | 15 | QTLVYKGMLTTPQLK |
| TcellP1533 | 16 | QTQWNQALEDLVRAYQ |
| TcellP1534 | 25 | QTSPANVVGTRQTLQGASVTVTGQG |
| TcellP1535 | 11 | QTVEDEARRMW |
| TcellP1536 | 15 | QTVEDEARRMWASAQ |
| TcellP1537 | 9 | QTYKWETFL |
| TcellP1538 | 15 | QTYKWETFLTSELPG |
| TcellP1539 | 12 | QTYKWETLLTSE |
| TcellP1540 | 15 | QVESTAGSLQGQWRG |
| TcellP1541 | 20 | QVESTAGSLQGQWRGAAGTA |
| TcellP1542 | 25 | QVESTAGSLQGQWRGAAGTAAQAAV |
| TcellP1543 | 15 | QVPSASMGRDIKVQF |
| TcellP1544 | 10 | QWNFAGIEAA |
| TcellP1545 | 15 | QWNFAGIEAAASAIQ |
| TcellP1546 | 20 | QWNNPAIQALNRDFTLPGER |
| TcellP1547 | 15 | QWRGAAGTAAQAAVV |
| TcellP1548 | 18 | RAAASGLYRGQQRRRRPG |
| TcellP1549 | 10 | RADEEQQQAL |
| TcellP1550 | 16 | RADEEQQQALSSQMGF |
| TcellP1551 | 13 | RALKRRNARTKAR |
| TcellP1552 | 25 | RANPLAAKGVCTYNDEQGVPFRVQG |
| TcellP1553 | 15 | RAQDDFSGWDINTPA |
| TcellP1554 | 18 | RAQRQRDLEAIRRAYAEM |
| TcellP1555 | 15 | RARQGFRDIEFHPTI |
| TcellP1556 | 16 | RAYQSMSGTHESNTMA |
| TcellP1557 | 15 | RCALHWFPGSHLLAC |
| TcellP1558 | 15 | RCALHWFPGSHLLHV |
| TcellP1559 | 20 | RCRRALRQIGVLERPVGDSS |
| TcellP1560 | 20 | RDGQLTIKAERTEQKDFDGR |
| TcellP1561 | 21 | RDGQLTIKAERTEQKDFDGRS |
| TcellP1562 | 20 | RDKFLSAATSSTPREAPYEL |
| TcellP1563 | 20 | RDTYAADGGRNGVFNFPPNG |
| TcellP1564 | 20 | RDVLAAGDFWGGAGSVACQE |
| TcellP1565 | 20 | RECVGSELLTEPEQLAAAHE |
| TcellP1566 | 25 | RELAYSVETTAESLEDELDELDENW |
| TcellP1567 | 15 | RELIRAFWPGALSLV |
| TcellP1568 | 9 | REPRRGPRL |
| TcellP1569 | 20 | RFFELAAEPTEVGQDQYVWH |
| TcellP1570 | 15 | RFQEAANKQKQELDE |
| TcellP1571 | 15 | RGAAGTAAQAAVVRF |
| TcellP1572 | 15 | RGKVVLIDFWAYPCI |
| TcellP1573 | 16 | RGLERGLNALADAVKV |
| TcellP1574 | 9 | RGPNAGSRF |
| TcellP1575 | 15 | RGRKRTLSDVLNEMR |
| TcellP1576 | 20 | RGRWLDPRHAGPATAADAGD |
| TcellP1577 | 24 | RGVAGKVVAGIGILAIRWRLPSGT |
| TcellP1578 | 20 | RHLPEHAIVQFVKAICSPMA |
| TcellP1579 | 11 | RKGRLAALAIA |
| TcellP1580 | 23 | RKHRIEDAVRNAKAAVEEGIVAG |
| TcellP1581 | 15 | RKIRAWGRRLMIGTA |
| TcellP1582 | 9 | RLANLLPLI |
| TcellP1583 | 9 | RLAPGGTTI |
| TcellP1584 | 9 | RLARRARNI |
| TcellP1585 | 20 | RLEDEMKEGRYEVRAELPGV |
| TcellP1586 | 15 | RLFNANAEEYHALSA |
| TcellP1587 | 9 | RLGLCALAL |
| TcellP1588 | 9 | RLGRLLNRI |
| TcellP1589 | 9 | RLPLVLPAV |
| TcellP1590 | 20 | RLSTEHWMGPASLSMAAAVQ |
| TcellP1591 | 20 | RLSVPASWSTAAPATAAGAA |
| TcellP1592 | 9 | RMAATAQVL |
| TcellP1593 | 9 | RMPPLGHEL |
| TcellP1594 | 18 | RNDPTQQIPKLVANNTRL |
| TcellP1595 | 15 | RNFQVIYEQANAHGQ |
| TcellP1596 | 15 | RNLPAGHGLNAQTGV |
| TcellP1597 | 20 | RNPLVRGTDRLEAVVMLLAV |
| TcellP1598 | 18 | RNRVGRQHGWPADVPSAE |
| TcellP1599 | 9 | RPANLAFFL |
| TcellP1600 | 9 | RPGCDAPAY |
| TcellP1601 | 12 | RPGLDELSFTLT |
| TcellP1602 | 24 | RPGLDELSFTLTGNPNRPDGGILT |
| TcellP1603 | 9 | RPKIDDHDY |
| TcellP1604 | 9 | RPKPDTETY |
| TcellP1605 | 9 | RPKPDYSAM |
| TcellP1606 | 9 | RPKVEGLEY |
| TcellP1607 | 9 | RPREATIIY |
| TcellP1608 | 9 | RPRLDSITY |
| TcellP1609 | 9 | RPRYEIFVY |
| TcellP1610 | 15 | RQAGVQYSRADEEQQ |
| TcellP1611 | 15 | RQSGATIADVLAEKE |
| TcellP1612 | 25 | RRAITRPTHFVPTTEGFTAAARAGL |
| TcellP1613 | 20 | RRARWVVRMLTSLLMFPGRD |
| TcellP1614 | 15 | RRDIELIHEQLADAG |
| TcellP1615 | 20 | RRIVDHLAHAGTHVDADVLL |
| TcellP1616 | 21 | RRLKLGDKVYFRHTKAGELCE |
| TcellP1617 | 11 | RRLVFIDRRPN |
| TcellP1618 | 15 | RRMWASAQNISGAGW |
| TcellP1619 | 15 | RRPLLVAVSWAIFAL |
| TcellP1620 | 15 | RRSFYRIFFDSGFTP |
| TcellP1621 | 15 | RSAFRLSPPVLSGAM |
| TcellP1622 | 9 | RSCPRLTIL |
| TcellP1623 | 15 | RSEFAYGSFVRTVSL |
| TcellP1624 | 20 | RSHVYAHQAQTRHPATATVI |
| TcellP1625 | 20 | RSLFPEFSELFAAFPSFAGL |
| TcellP1626 | 15 | RSPISNMVSMANNHM |
| TcellP1627 | 15 | RSRPRRTTRRMDRRT |
| TcellP1628 | 20 | RTEQKDFDGRSEFAYGSFVR |
| TcellP1629 | 15 | RTISEAGQAMASTEG |
| TcellP1630 | 9 | RTVSLPVGA |
| TcellP1631 | 16 | RVAQIRQEIENSDSDY |
| TcellP1632 | 25 | RVCDIHLDVPLYWQCWKLDSPIIAR |
| TcellP1633 | 15 | RVIEDALRFYFESHE |
| TcellP1634 | 18 | RVILHSPYGLRVHGPLAL |
| TcellP1635 | 20 | RVIPIAASTRESFTVNESRT |
| TcellP1636 | 15 | RVLLAGWEQFDEPVD |
| TcellP1637 | 15 | RVPEDLLAMVVAVEQ |
| TcellP1638 | 20 | RVTDVLDTCRQQKGHVFEGG |
| TcellP1639 | 6 | RYPNVT |
| TcellP1640 | 7 | RYPNVTI |
| TcellP1641 | 20 | SAAFVVASGSLPPGVAADYY |
| TcellP1642 | 15 | SAAGSYAAAEAANAS |
| TcellP1643 | 9 | SAAIAGLFG |
| TcellP1644 | 20 | SAASAFQSVVWGLTTGSWIG |
| TcellP1645 | 15 | SAGMRRITISLDTLR |
| TcellP1646 | 20 | SAIAATEARYGEMWAQDASA |
| TcellP1647 | 15 | SAIQGNVTSIHSLLD |
| TcellP1648 | 25 | SAIQGNVTSIHSLLDEGKQSLTKLA |
| TcellP1649 | 10 | SALQNAASIA |
| TcellP1650 | 20 | SALTLAIYHPQQFVYAGAMS |
| TcellP1651 | 15 | SAMILAAYHPQQFIY |
| TcellP1652 | 20 | SAMILAAYHPQQFIYAGSLS |
| TcellP1653 | 15 | SARLRLLRDRLVEGV |
| TcellP1654 | 14 | SASMGRDIKVQFQG |
| TcellP1655 | 18 | SAWQGDTGITYQAWQAQW |
| TcellP1656 | 15 | SAWQGDTGITYQGWQ |
| TcellP1657 | 20 | SAWQGDTGITYQGWQTQWNQ |
| TcellP1658 | 25 | SDPALLAEIRQSLDATKGLTSVHVA |
| TcellP1659 | 15 | SEAGQAMASTEGNVT |
| TcellP1660 | 14 | SEFAYGSFVRTVSL |
| TcellP1661 | 15 | SEFAYGSFVRTVSLP |
| TcellP1662 | 20 | SEFAYGSFVRTVSLPVGADE |
| TcellP1663 | 15 | SEFNEVFFNDVFVPD |
| TcellP1664 | 15 | SELPGWLQANRHVKP |
| TcellP1665 | 19 | SFAAAGLAALAVAVSPPAA |
| TcellP1666 | 20 | SFLKVLAGMHNEIVGDIKRA |
| TcellP1667 | 20 | SGAGWSGMAEATSLDTMTQM |
| TcellP1668 | 20 | SGETTTAAGTTASPGAASGP |
| TcellP1669 | 18 | SGGNNSPAVYLLDGLRAQ |
| TcellP1670 | 20 | SGGYNANSMWGPSSDPAWKR |
| TcellP1671 | 18 | SGLSIVMPVGGQSSFYSD |
| TcellP1672 | 20 | SGNFLLPDAQSIQAAAAGFA |
| TcellP1673 | 20 | SGRTYGEDEIRAEFGVPRRP |
| TcellP1674 | 15 | SGSEAYQGVQQKWDA |
| TcellP1675 | 25 | SGSEAYQGVQQKWDATATELNNALQ |
| TcellP1676 | 16 | SGTHESNTMAMLARDG |
| TcellP1677 | 9 | SGVGNDLVL |
| TcellP1678 | 20 | SHITNPAGLAHQAAAVGQAG |
| TcellP1679 | 20 | SHLISDVADAVLSFASPVMS |
| TcellP1680 | 23 | SIDLGSGSIQLTQSKWNEPVNVD |
| TcellP1681 | 18 | SIHSLLDEGKQSLTKLAA |
| TcellP1682 | 9 | SIIIPTLNV |
| TcellP1683 | 15 | SILDMRQLFDGIDLS |
| TcellP1684 | 20 | SKFNDTLQEFETTRSSTGTG |
| TcellP1685 | 15 | SLAVKTFEDLFAELG |
| TcellP1686 | 15 | SLFAALNIAAVVAVL |
| TcellP1687 | 9 | SLIDLLHKI |
| TcellP1688 | 25 | SLKRTRITIAVNADSMATWFSAVFD |
| TcellP1689 | 15 | SLNLRFVSTYRSPDR |
| TcellP1690 | 20 | SLPEIAANHITQAVLTATNF |
| TcellP1691 | 15 | SLQGQWRGAAGTAAQ |
| TcellP1692 | 9 | SLRNWIATL |
| TcellP1693 | 20 | SLVRHRRQQRDALCLSSTQI |
| TcellP1694 | 9 | SLWKDGAPL |
| TcellP1695 | 9 | SLYFGGICV |
| TcellP1696 | 9 | SMADRAENL |
| TcellP1697 | 9 | SMAGRAGQL |
| TcellP1698 | 9 | SMAGSSAMI |
| TcellP1699 | 10 | SMAGSSAMIL |
| TcellP1700 | 15 | SMAGSSAMILAAYHP |
| TcellP1701 | 9 | SMFAAVQAL |
| TcellP1702 | 15 | SMSGTHESNTMAMLA |
| TcellP1703 | 20 | SMSGTHESNTMAMLARDGAE |
| TcellP1704 | 9 | SMTNSGVSM |
| TcellP1705 | 25 | SNNPMLSTLTSALSGKLNPDVNLVD |
| TcellP1706 | 25 | SNVAGVNTPAIADLDAQYDQYRARN |
| TcellP1707 | 15 | SPALYLLDGLRAQDD |
| TcellP1708 | 15 | SPEEILDVIADFEAM |
| TcellP1709 | 20 | SPGFGTTVDFPAVPGALGEN |
| TcellP1710 | 9 | SPKETWLRL |
| TcellP1711 | 14 | SPSMGRDIKVQFQS |
| TcellP1712 | 14 | SPSMGRDIPVAFLA |
| TcellP1713 | 20 | SPYVAWMSVTAGQAELTAAQ |
| TcellP1714 | 15 | SQGMGPSLIGLAMGD |
| TcellP1715 | 9 | SQIMYNYPA |
| TcellP1716 | 18 | SQLIEKPVAPSVMPAAAA |
| TcellP1717 | 20 | SQRIKSLEQQVGQVLVVREK |
| TcellP1718 | 15 | SQVIEAVNLFRANVI |
| TcellP1719 | 17 | SRADEEQQQALSSQMGF |
| TcellP1720 | 15 | SRFYFLTGRGALLQL |
| TcellP1721 | 25 | SRIDGTHQTLQGADLTVIGARDDLM |
| TcellP1722 | 20 | SSAGLMVAAASPYVAWMSVT |
| TcellP1723 | 20 | SSDPAWERNDPTQQIPKLVA |
| TcellP1724 | 11 | SSFYRPTQPGS |
| TcellP1725 | 15 | SSFYSDWYSPACGKA |
| TcellP1726 | 20 | SSGLGAGVAANLGRAASVGS |
| TcellP1727 | 25 | SSILTYHVIAGQASPSRIDGTHQTL |
| TcellP1728 | 20 | SSIPMTAVSGVGAGDAMVAA |
| TcellP1729 | 20 | SSLLTSILTYHVVAGQTSPA |
| TcellP1730 | 25 | SSLLTSILTYHVVAGQTSPANVVGT |
| TcellP1731 | 18 | SSNLKFQDAYKPAGGHNA |
| TcellP1732 | 15 | SSTATSGAAVVSPAE |
| TcellP1733 | 25 | SSTAVIPGYPVAGQVWEATATVNAI |
| TcellP1734 | 15 | SSTHEANTMAMMARD |
| TcellP1735 | 15 | SSYAATEVANAAAAS |
| TcellP1736 | 15 | SSYAATEVANAAAGQ |
| TcellP1737 | 15 | STAGSLQGQWRGAAG |
| TcellP1738 | 11 | STEGNVTGMFA |
| TcellP1739 | 15 | STHEANTMAMMARDT |
| TcellP1740 | 15 | STIFPFRRLFMVADV |
| TcellP1741 | 15 | STIFPFRRLFMVAEV |
| TcellP1742 | 15 | STNIRQAGVQYSRAD |
| TcellP1743 | 20 | STPLILDTSGGGLQHISSGV |
| TcellP1744 | 20 | STPREAPYELNITSATYQSA |
| TcellP1745 | 25 | STSRVLDPAAGVTQLLSGVTNLQAQ |
| TcellP1746 | 10 | STVKDLLPLL |
| TcellP1747 | 16 | SVAVKAPGFGDRRKAM |
| TcellP1748 | 20 | SVAVSEGKPTEKHIQIRSTN |
| TcellP1749 | 25 | SVKMLDPGAKSARPATVWIAQDGSH |
| TcellP1750 | 18 | SVMPAAAAGSSATGGAAP |
| TcellP1751 | 15 | SVSWSNPNDWWLVRL |
| TcellP1752 | 20 | SVTITGFGVFEQRRRAARVA |
| TcellP1753 | 10 | SVVMGVNKAK |
| TcellP1754 | 15 | SWEYWGAQLNAMKPD |
| TcellP1755 | 23 | SWRILDGLIAVMMVALGISLTVT |
| TcellP1756 | 20 | TAACNRDDVERFFELAAEPT |
| TcellP1757 | 8 | TAAGNVNI |
| TcellP1758 | 10 | TAAGNVNIAI |
| TcellP1759 | 10 | TAAQAAVVRF |
| TcellP1760 | 15 | TAAQAAVVRFQEAAN |
| TcellP1761 | 9 | TAGSLQGQW |
| TcellP1762 | 15 | TAGSLQGQWRGAAGT |
| TcellP1763 | 13 | TAINSLVTATHGA |
| TcellP1764 | 16 | TAKEKPQEGTVVAVGP |
| TcellP1765 | 17 | TASGLVIPDTAKEKPQE |
| TcellP1766 | 20 | TASPGAASGPKVVIDGKDQN |
| TcellP1767 | 20 | TATEALLPFEDAPLITNPGG |
| TcellP1768 | 15 | TATELNNALQNLART |
| TcellP1769 | 20 | TATELNNALQNLARTISEAG |
| TcellP1770 | 25 | TATELNNALQNLARTISEAGQAMAS |
| TcellP1771 | 20 | TATNILGQNVSAIAATEARY |
| TcellP1772 | 20 | TATSAPPRTKITVPARWVVN |
| TcellP1773 | 15 | TCNYGQVVAALNATD |
| TcellP1774 | 15 | TDAATLAQEAGNFER |
| TcellP1775 | 16 | TDAIAGRVAQIRQEIE |
| TcellP1776 | 15 | TDAMRKVTGMHVRLA |
| TcellP1777 | 15 | TDNGAMIAAFAAQLV |
| TcellP1778 | 15 | TELNNALQNLARTIS |
| TcellP1779 | 15 | TEQQWNFAGIEAAAS |
| TcellP1780 | 20 | TEVGQDQYVWHPIVNPEASP |
| TcellP1781 | 7 | TFGLQLE |
| TcellP1782 | 9 | TFGLQLELT |
| TcellP1783 | 17 | TFGLQLELTEGMRFDKG |
| TcellP1784 | 20 | TFLTSELPGWLQANRHVKPT |
| TcellP1785 | 15 | TGIFGLVLVICVVLI |
| TcellP1786 | 18 | TGSAAIGLSMAGSSAMIL |
| TcellP1787 | 15 | TGSAVVGLSMAASSA |
| TcellP1788 | 17 | TGSGAGIAQAAAGTVNI |
| TcellP1789 | 18 | TGSPSAAIGLSMAGSSAM |
| TcellP1790 | 20 | TGTGLQGVTSGLANNLLAAA |
| TcellP1791 | 15 | TGVVPAAADEVSALT |
| TcellP1792 | 9 | THEANTMAM |
| TcellP1793 | 15 | THEANTMAMMARDTA |
| TcellP1794 | 15 | THSWEYWGAQLNAMK |
| TcellP1795 | 19 | THSWEYWGAQLNAMKGDLQ |
| TcellP1796 | 20 | THSWEYWGAQLNAMKGDLQS |
| TcellP1797 | 19 | THSWEYWGAQLNAMKPDLQ |
| TcellP1798 | 20 | THSWEYWGAQLNAMKPDLQR |
| TcellP1799 | 20 | THSWPYWNEQLVAMKADIQH |
| TcellP1800 | 10 | TIAYDEEARR |
| TcellP1801 | 20 | TIDSVGQTIAGATISGVGND |
| TcellP1802 | 20 | TIGDTTIPAGRRVLLLYGSA |
| TcellP1803 | 9 | TIPEALAAV |
| TcellP1804 | 20 | TIPIALTEMDYFIRMWNQAA |
| TcellP1805 | 15 | TISEAGQAMASTEGN |
| TcellP1806 | 16 | TISEAGQAMASTEGNV |
| TcellP1807 | 20 | TKAPAKKAVKATKSPAKKVT |
| TcellP1808 | 25 | TKGLTSVHVAVRTTGKVDSLLGITS |
| TcellP1809 | 10 | TLAGKGISVV |
| TcellP1810 | 9 | TLAPQVEPL |
| TcellP1811 | 25 | TLGQLGEMSGPMQQLTQPLQQVTSL |
| TcellP1812 | 20 | TLHSMLKGFAPAAAQAVETA |
| TcellP1813 | 20 | TLRATVERALQQLVAAAAEQ |
| TcellP1814 | 18 | TLSAALDAQAVELTARLN |
| TcellP1815 | 16 | TLVVNKIRGTFKSVAV |
| TcellP1816 | 18 | TMFAEQMKILVPVFTSND |
| TcellP1817 | 9 | TMITFRLRL |
| TcellP1818 | 14 | TNFFGINTIPIALT |
| TcellP1819 | 15 | TNFFGINTIPIALTE |
| TcellP1820 | 13 | TNIRQAGVQYSRA |
| TcellP1821 | 15 | TNIRQAGVQYSRADE |
| TcellP1822 | 16 | TNSSLLTSILTYHVVA |
| TcellP1823 | 18 | TPAFEWYYQSGLSIVMPV |
| TcellP1824 | 20 | TPAIAVNEAEYGEMWAQDAA |
| TcellP1825 | 17 | TPMVVWLQTASTQAKTR |
| TcellP1826 | 25 | TPSLPEIAANHITQAVLTATNFFGI |
| TcellP1827 | 15 | TPVQSQRVDPSAASG |
| TcellP1828 | 13 | TPVRLAEVLVTCA |
| TcellP1829 | 20 | TQAMATTPSLPEIAANHITQ |
| TcellP1830 | 13 | TQIDQVESTAGSL |
| TcellP1831 | 10 | TQLLMAAASA |
| TcellP1832 | 25 | TQPLQQVTSLFSQVGGTGGGNPADE |
| TcellP1833 | 15 | TQVLVPRSAIDSMLA |
| TcellP1834 | 16 | TQVRDLIIQASQAGAA |
| TcellP1835 | 20 | TRHPATATVIDHEGVIDSNT |
| TcellP1836 | 25 | TRMGYGPLVRAATGVTRVWTSDEAQ |
| TcellP1837 | 18 | TRPGLVAPAPLAQEREED |
| TcellP1838 | 12 | TRRRLLAVLIAL |
| TcellP1839 | 11 | TRVWVWSPTNP |
| TcellP1840 | 20 | TRVWVWSPTNPGASDPAAMI |
| TcellP1841 | 9 | TSADRAVVL |
| TcellP1842 | 20 | TSAGGDPVAITIDSVGQTIA |
| TcellP1843 | 20 | TSGLANNLLAAAGAYLKADD |
| TcellP1844 | 18 | TSHEIDDDTAELALLSMH |
| TcellP1845 | 15 | TSIHSLLDEGKQSLT |
| TcellP1846 | 15 | TSPAAPVTTAAMADP |
| TcellP1847 | 18 | TSPLSNHPLAGGSGPSAG |
| TcellP1848 | 25 | TTIFPDHVVGRVGALLALAALIHRD |
| TcellP1849 | 17 | TTLGMHCGSFGSAPSNG |
| TcellP1850 | 21 | TTPASSPVTLAETGSTLLYPL |
| TcellP1851 | 15 | TVAWTMLGVALSAYE |
| TcellP1852 | 15 | TVEDEARRMWASAQN |
| TcellP1853 | 25 | TVGQVVLGWKVSDLKSSTAVIPGYP |
| TcellP1854 | 25 | TVIGARDDLMVNNAGLVCGGVHTAN |
| TcellP1855 | 15 | TVRYRIRRIEQLLST |
| TcellP1856 | 20 | TVSLLTIPFAAAAGTAVQDS |
| TcellP1857 | 15 | TVSLPVGADEDDIKA |
| TcellP1858 | 20 | TVSLPVGADEDDIKATYDKG |
| TcellP1859 | 25 | TVWIAQDGSHHLVRASIDLGSGSIQ |
| TcellP1860 | 13 | TVYMIDSVLMPPA |
| TcellP1861 | 9 | TYQAWQAQW |
| TcellP1862 | 15 | VAAMPGLLQRLSSAA |
| TcellP1863 | 15 | VADIHFQPRYIFAAI |
| TcellP1864 | 16 | VAFRAGLVMEAGSKVT |
| TcellP1865 | 20 | VAGISGRVQLTHGSFTSKFN |
| TcellP1866 | 15 | VAKVNIKPLEDKILV |
| TcellP1867 | 16 | VAKVNIKPLEDKILVQ |
| TcellP1868 | 15 | VANNTRLWVYCGNGT |
| TcellP1869 | 16 | VANNTRLWVYCGNGTP |
| TcellP1870 | 20 | VANNTRLWVYCGNGTPNELG |
| TcellP1871 | 20 | VANRALLAELTATNILGQNV |
| TcellP1872 | 20 | VCGGVSTANATVYMIDSVLM |
| TcellP1873 | 20 | VDAVSSTQDQITITKNGAPA |
| TcellP1874 | 20 | VDEPAPPARAIADAALAALG |
| TcellP1875 | 20 | VDFGALPPEVNSARMYGGAG |
| TcellP1876 | 15 | VDHLERMLSLDNAFT |
| TcellP1877 | 15 | VDLAKSLRIAAKIYS |
| TcellP1878 | 15 | VDLIAHGTAARIYRL |
| TcellP1879 | 15 | VDVEDGRVIVDEYQR |
| TcellP1880 | 9 | VEAFRTRPL |
| TcellP1881 | 15 | VEDEARRMWASAQNI |
| TcellP1882 | 15 | VEGAGDTDAIAGRVA |
| TcellP1883 | 18 | VELTARLNSLGEAWTGGG |
| TcellP1884 | 25 | VELTARLNSLGEAWTGGGSDKALAA |
| TcellP1885 | 8 | VESTAGSL |
| TcellP1886 | 15 | VEYLQVPSPSMGRDI |
| TcellP1887 | 15 | VFDFPDSGTHSWEYW |
| TcellP1888 | 18 | VFNFPPNGTHSWEYWGAQ |
| TcellP1889 | 20 | VGAGDAMVAAITVGLSRGWS |
| TcellP1890 | 15 | VGGAGFATDFEPVDH |
| TcellP1891 | 20 | VGIVTEADIARHLPEHAIVQ |
| TcellP1892 | 11 | VGLGNVNGVTL |
| TcellP1893 | 9 | VGSLNGTYV |
| TcellP1894 | 15 | VGSSHGPHGLLRGLP |
| TcellP1895 | 15 | VHAQTVEDEARRMWA |
| TcellP1896 | 12 | VHFQPLPPAVVK |
| TcellP1897 | 15 | VHVADFVDVFPGQRI |
| TcellP1898 | 15 | VHVSFVMAYPEMLAA |
| TcellP1899 | 33 | VIKAGAATEVELKERKHRIEDAVRNAKAAVEEG |
| TcellP1900 | 25 | VITMLWHAMPPELNTARLMAGAGPA |
| TcellP1901 | 20 | VKPTSVPAFRPGAQFKAVVS |
| TcellP1902 | 16 | VKRGLTVAVAGAAILV |
| TcellP1903 | 20 | VKRGLTVAVAGAAILVAGLS |
| TcellP1904 | 15 | VKVTRSALQNAASIA |
| TcellP1905 | 9 | VLAGSVDEL |
| TcellP1906 | 20 | VLERPVGDSSDCGTIRVGSF |
| TcellP1907 | 19 | VLFAATAAAAAAVDRGDPP |
| TcellP1908 | 9 | VLGRLDQKL |
| TcellP1909 | 9 | VLLPGLPYL |
| TcellP1910 | 10 | VLMGGVPGVE |
| TcellP1911 | 9 | VLPACLGIL |
| TcellP1912 | 9 | VLRPGGHFL |
| TcellP1913 | 20 | VLSFASPVMSAADTGLEAVR |
| TcellP1914 | 20 | VLSGAMGPFMHTGLYVAQSW |
| TcellP1915 | 18 | VLTATNFFGINTIPIALT |
| TcellP1916 | 25 | VLTATNFFGINTIPIALTEMDYFIR |
| TcellP1917 | 10 | VLTDGNPPEV |
| TcellP1918 | 9 | VMADRTRHL |
| TcellP1919 | 9 | VMAPLGPIL |
| TcellP1920 | 9 | VMATRRNVL |
| TcellP1921 | 9 | VMMSEIAGL |
| TcellP1922 | 15 | VMRLYPVRLTTTMTR |
| TcellP1923 | 9 | VMTTVLATL |
| TcellP1924 | 12 | VNKIRGTFKSVA |
| TcellP1925 | 7 | VNKSFEI |
| TcellP1926 | 13 | VNLVDTLNSGQYT |
| TcellP1927 | 15 | VPSPSMGRDIKVQFQ |
| TcellP1928 | 20 | VPSPSMGRDIKVQFQSGGAN |
| TcellP1929 | 9 | VPWQPAFVF |
| TcellP1930 | 16 | VQQKWDATATELNNAL |
| TcellP1931 | 15 | VQYSRADEEQQQALS |
| TcellP1932 | 20 | VQYSRADEEQQQALSSQMGF |
| TcellP1933 | 15 | VRFQEAANKQKQELD |
| TcellP1934 | 15 | VRNLPAGHGLNAQTG |
| TcellP1935 | 20 | VRVAAAAYETAYGLTVPPPV |
| TcellP1936 | 15 | VSASLARANKIGALS |
| TcellP1937 | 20 | VSDLKSSTAVIPGYPVAGQV |
| TcellP1938 | 20 | VSIAPNAGLDPVNYQNFAVT |
| TcellP1939 | 25 | VSQDTSPKPATSPAAPVTTAAMADP |
| TcellP1940 | 15 | VTANRAELKALIASN |
| TcellP1941 | 20 | VTGSVVCTTAAGNVNIAIGG |
| TcellP1942 | 16 | VTLLQAAPTLDELKLE |
| TcellP1943 | 15 | VTSIHSLLDEGKQSL |
| TcellP1944 | 24 | VTSIHSLLDEGKQSLTKLAAAWGG |
| TcellP1945 | 15 | VTTQPEALAAAAANL |
| TcellP1946 | 15 | VTVDAAVLAAIDADA |
| TcellP1947 | 20 | VTVTFMLVWLINHHGWSVAQ |
| TcellP1948 | 15 | VVAEKVRNLPAGHGL |
| TcellP1949 | 15 | VVCTTAAGNVNIAIG |
| TcellP1950 | 25 | VVHPAVVQANRVRTWLLAVSNVFGQ |
| TcellP1951 | 15 | VVMPVGGQSSFYSDW |
| TcellP1952 | 11 | VVNKIRGTFKS |
| TcellP1953 | 16 | VVNKIRGTFKSVAVKA |
| TcellP1954 | 15 | VVRFQEAANKQKQEL |
| TcellP1955 | 18 | VVRFQEAANKQKQELDEI |
| TcellP1956 | 20 | VVRPTEKMRCGAPRYDPGGG |
| TcellP1957 | 20 | VVSLGSQGALLATRHASHRF |
| TcellP1958 | 15 | VVSREHLIQQAIAAN |
| TcellP1959 | 20 | VVVTKDETTIVEGAGDTDAI |
| TcellP1960 | 18 | VWLQTASTQAKTRAMQAT |
| TcellP1961 | 25 | VWLQTASTQAKTRAMQATAQAAAYT |
| TcellP1962 | 8 | VYAGAMSG |
| TcellP1963 | 9 | VYAGAMSGL |
| TcellP1964 | 15 | VYEDLLAAGVADPVK |
| TcellP1965 | 16 | VYEDLLAAGVADPVKV |
| TcellP1966 | 9 | VYLDTVVLL |
| TcellP1967 | 15 | VYLLDGLRAQDDYNG |
| TcellP1968 | 15 | VYLVWRFIVPLVGRL |
| TcellP1969 | 15 | VYLVWRFIVPVVGRL |
| TcellP1970 | 9 | VYYPGENQI |
| TcellP1971 | 15 | WDEDGEKRIPLDVAE |
| TcellP1972 | 20 | WDINTPAFEEYYQSGLSVIM |
| TcellP1973 | 20 | WDINTPAFEWYDQSGLSVVM |
| TcellP1974 | 15 | WDINTPAFEWYYQSG |
| TcellP1975 | 20 | WDINTPAFEWYYQSGLSIVM |
| TcellP1976 | 20 | WDQAYRKPITYDTLWQADTD |
| TcellP1977 | 25 | WEATATVNAIRGSVTPAVSQFNART |
| TcellP1978 | 15 | WFINWYLPISQLFYN |
| TcellP1979 | 9 | WGAQLNAMK |
| TcellP1980 | 20 | WGLTTGSWIGSSAGLMVAAA |
| TcellP1981 | 15 | WGRRLMIGTAAAVVL |
| TcellP1982 | 17 | WIFGWNRLPRLVHLACI |
| TcellP1983 | 25 | WKLDSPIIARITDTVRAAASGLYRG |
| TcellP1984 | 21 | WLKLGLVEFGGVAKLNAEVMS |
| TcellP1985 | 9 | WLPPLLTNL |
| TcellP1986 | 15 | WLQANRHVKPTGSAV |
| TcellP1987 | 9 | WLYPGAQNL |
| TcellP1988 | 9 | WMCDRAVDL |
| TcellP1989 | 15 | WNFAGIEAAASAIQG |
| TcellP1990 | 23 | WNFAGIEAAASAIQGNVTSIHSL |
| TcellP1991 | 20 | WNGIAVEVSTAASSVGSVIT |
| TcellP1992 | 18 | WPADVPSAEQRRAQRQRD |
| TcellP1993 | 9 | WPTLIGLAM |
| TcellP1994 | 9 | WQAQWNQAM |
| TcellP1995 | 9 | WQGDTGITY |
| TcellP1996 | 16 | WQGDTGITYQGWQTQW |
| TcellP1997 | 15 | WQRNDPLLNVGKLIA |
| TcellP1998 | 15 | WRGAAGTAAQAAVVR |
| TcellP1999 | 22 | WRRRPLSSALLSFGLLLGGLPL |
| TcellP2000 | 17 | WSEYSRFVGDVFGAPLA |
| TcellP2001 | 15 | WTNTPTKWDNSFLEI |
| TcellP2002 | 15 | WVYCGNGKPSDLGGN |
| TcellP2003 | 18 | WVYCGNGTPNELGGANIP |
| TcellP2004 | 9 | WYDQSGLSV |
| TcellP2005 | 18 | WYSPACGKAGCQTYKWET |
| TcellP2006 | 9 | WYYQSGLSI |
| TcellP2007 | 15 | YAAALVAMPTLAELA |
| TcellP2008 | 9 | YAEMWAQDA |
| TcellP2009 | 18 | YAGSLSALLDPSQGMGPS |
| TcellP2010 | 20 | YAGTLQSLGADIASEQAVLS |
| TcellP2011 | 18 | YAGTLQSLGAEIAVEQAA |
| TcellP2012 | 20 | YAIVNNRQKDAATAQTLQAF |
| TcellP2013 | 15 | YASVEAANASPLQVA |
| TcellP2014 | 17 | YDAYFTDAGGITPGNSV |
| TcellP2015 | 20 | YDQSGLSVVMPVGGQSSFYS |
| TcellP2016 | 20 | YDTLWQADTDPLPVVFPIVQ |
| TcellP2017 | 21 | YEKIGAELVKEVAKKTDDVAG |
| TcellP2018 | 20 | YEVRAELPGVDPDKDVDIMV |
| TcellP2019 | 15 | YFIRMWNQAALAMEV |
| TcellP2020 | 15 | YFVTDPERQEAVLED |
| TcellP2021 | 20 | YGEMWAQDAAAMFGYAATAA |
| TcellP2022 | 15 | YGSFVRTVSLPVGAD |
| TcellP2023 | 15 | YHPQQFVYAGAMSGL |
| TcellP2024 | 18 | YKPAGGHNAVFNFPPNGT |
| TcellP2025 | 15 | YLAWLSTAAAQAEQA |
| TcellP2026 | 15 | YLGLEVLTRARAALT |
| TcellP2027 | 9 | YLLADTFTV |
| TcellP2028 | 15 | YLLDFLRQSGNTPIV |
| TcellP2029 | 9 | YLLDGLRAQ |
| TcellP2030 | 18 | YLLDGLRAQDDYNGWDIN |
| TcellP2031 | 18 | YLQVPSPSMGRDIKVQFQ |
| TcellP2032 | 15 | YNGEEYLILSARDVL |
| TcellP2033 | 20 | YNGEEYLILSARDVLAVVSK |
| TcellP2034 | 20 | YNINISLPSYYPDQKSLENY |
| TcellP2035 | 15 | YNYPAMMAHAGDMAG |
| TcellP2036 | 21 | YPFEGLRILDLGIIVAGGELS |
| TcellP2037 | 10 | YPIINYEYAI |
| TcellP2038 | 16 | YPIINYEYAIVNNRQK |
| TcellP2039 | 25 | YPITGKLGSELTMTDTVGQVVLGWK |
| TcellP2040 | 18 | YQAWQAQWNQAMEDLVRA |
| TcellP2041 | 15 | YQGVQQKWDATATEL |
| TcellP2042 | 20 | YQGVQQKWDATATELNNALQ |
| TcellP2043 | 15 | YQGWQTQWNQALEDL |
| TcellP2044 | 20 | YQGWQTQWNQALEDLVRAYQ |
| TcellP2045 | 9 | YQSGLSIVM |
| TcellP2046 | 15 | YRIAARPGAVTRRAA |
| TcellP2047 | 25 | YRVLWQAAGPDTISGATIPQGEQST |
| TcellP2048 | 15 | YSKYGGTEIKYNGEE |
| TcellP2049 | 15 | YVAWMSATAALAREA |
| TcellP2050 | 15 | YWGAQLNAMKGDLQS |
| TcellP2051 | 20 | YWGAQLNAMKGDLQSSLGAG |
| TcellP2052 | 10 | YYQSGLSIVM |
| TcellP2053 | 15 | YYQSGLSIVMPVGGQ |
| TcellP2054 | 20 | YYQSGLSIVMPVGGQSSFYS |
| TcellP2055 | 20 | YYQSGLSVIMPVGGQSSFYT |
| BcellP0001 | 15 | AAAGDLVGPGCAEYA |
| BcellP0002 | 16 | AAASAIQGNVTSIHSL |
| BcellP0003 | 15 | AAASLAAIAIAFLAG |
| BcellP0004 | 23 | AAAWGGSGSEAYQGVQQKWDATA |
| BcellP0005 | 20 | AAGFASKTPANQAISMIDGP |
| BcellP0006 | 7 | AAGNVNI |
| BcellP0007 | 15 | AAGTAAQAAVVRFQE |
| BcellP0008 | 15 | AAHKGLMNIALAISA |
| BcellP0009 | 20 | AAIGLSMAGSSAMILAAYHP |
| BcellP0010 | 21 | AAIGLSMAGSSAMILAAYHPQ |
| BcellP0011 | 15 | AAPAKKAAPAKKAAA |
| BcellP0012 | 15 | AAQAVQTAAQNGVRA |
| BcellP0013 | 20 | AASGPKVVIDGKDQNVTGSV |
| BcellP0014 | 9 | AASIAGLFL |
| BcellP0015 | 8 | AAVAEAEG |
| BcellP0016 | 24 | AAVQAAWAVLVAYFNRLRCGTGDY |
| BcellP0017 | 20 | AAVVLPGLVGLAGGAATAGA |
| BcellP0018 | 15 | ACGGGTNSSSSGAGG |
| BcellP0019 | 15 | ADEEQQQALSSQMGF |
| BcellP0020 | 8 | AEAEGGTW |
| BcellP0021 | 10 | AEGDTVIYSK |
| BcellP0022 | 15 | AEGDTVIYSKYGGTE |
| BcellP0023 | 8 | AEGGTWRI |
| BcellP0024 | 8 | AEYAAANP |
| BcellP0025 | 15 | AFDLDRLLFKRNICH |
| BcellP0026 | 15 | AFLHWAITDGNKASF |
| BcellP0027 | 15 | AGAGTVATTPASSPV |
| BcellP0028 | 15 | AGFTAPATTLLSALG |
| BcellP0029 | 15 | AGGGHNGVFDFPDSG |
| BcellP0030 | 15 | AGGYKAADMWGPSSD |
| BcellP0031 | 20 | AGGYKAADMWGPSSDPAWER |
| BcellP0032 | 10 | AGLFLTTEAV |
| BcellP0033 | 8 | AGPGVAGS |
| BcellP0034 | 8 | AGSGASDL |
| BcellP0035 | 15 | AGSLQGQWRGAAGTA |
| BcellP0036 | 15 | AGTAVQDSRSHVYAH |
| BcellP0037 | 15 | AGTVNIGASDAYLSE |
| BcellP0038 | 20 | AHAIALFLNQVHRSRAQFAK |
| BcellP0039 | 15 | AIANAYWSPQARRRF |
| BcellP0040 | 21 | AIWRRIRVVPFEVVIPADEQD |
| BcellP0041 | 15 | AKFSDTIGKRDEQIT |
| BcellP0042 | 13 | AKVNIKPLEDKIL |
| BcellP0043 | 15 | AKVRRERMGHIELAA |
| BcellP0044 | 20 | ALLDPSQGMGPSLIGLAMGD |
| BcellP0045 | 15 | ALMSGNFSNGILWRG |
| BcellP0046 | 15 | ALTGLGDKFGESIVN |
| BcellP0047 | 10 | ALVREGLRNV |
| BcellP0048 | 13 | ALVREGLRNVAAG |
| BcellP0049 | 15 | AMEDLVRAYHAMSST |
| BcellP0050 | 15 | AMMARDTAEAAKWGG |
| BcellP0051 | 15 | ANRAVKPTGSAAIGL |
| BcellP0052 | 20 | ANRAVKPTGSAAIGLSMAGS |
| BcellP0053 | 15 | ANRHVKPTGSAVVGL |
| BcellP0054 | 8 | ANVVGTRQ |
| BcellP0055 | 9 | APDPPLSRR |
| BcellP0056 | 15 | APYNVRRLPPPVVEP |
| BcellP0057 | 15 | AQAAVVRFQEAANKQ |
| BcellP0058 | 15 | AQAQHLTMANIVTSA |
| BcellP0059 | 10 | ARDVLAVVSK |
| BcellP0060 | 15 | ARLVVLASGTGSLLR |
| BcellP0061 | 20 | ARPADYMLALSVAGGSLPVV |
| BcellP0062 | 20 | ASATKDGSHYKITGTATGVD |
| BcellP0063 | 8 | ASDLRSSS |
| BcellP0064 | 15 | ASFLDQVHFQPLPPA |
| BcellP0065 | 20 | ASMLGTVTNSPGVPAVPWGA |
| BcellP0066 | 24 | ATELNNALQNLARTISEAGQAMAS |
| BcellP0067 | 20 | ATGVDMANPMSPVNKSFEIE |
| BcellP0068 | 15 | ATIPQGEQSTGKIYF |
| BcellP0069 | 15 | ATVLAQALVREGLRN |
| BcellP0070 | 20 | AVAASNNPELTTLTAALSGQ |
| BcellP0071 | 8 | AVAEAEGG |
| BcellP0072 | 20 | AVAVSPPAAAGDLVGPGCAE |
| BcellP0073 | 10 | AVGPGRWDED |
| BcellP0074 | 16 | AVLEDPYILLVSSKVS |
| BcellP0075 | 13 | AVVRFQEAANKQK |
| BcellP0076 | 24 | AWGGSGSEAYQGVQQKWDATATEL |
| BcellP0077 | 15 | AYVITSVDEEMRHNE |
| BcellP0078 | 15 | CGGVSTANATVYMID |
| BcellP0079 | 20 | CGNGTPNELGGANIPAEFLE |
| BcellP0080 | 8 | CPPRRRAG |
| BcellP0081 | 15 | CVAYIGISFLDQASQ |
| BcellP0082 | 8 | CVTGHWRC |
| BcellP0083 | 20 | DAATAQTLQAFLHWAITDGN |
| BcellP0084 | 8 | DAAVAEAE |
| BcellP0085 | 13 | DDLVGAGVIDAVA |
| BcellP0086 | 15 | DDVAGDGTTTATVLA |
| BcellP0087 | 9 | DEGKQSLTK |
| BcellP0088 | 15 | DEMTRVIWKLIKDML |
| BcellP0089 | 20 | DFWATWCGPCKMVAPVLEEI |
| BcellP0090 | 10 | DGEKRIPLDV |
| BcellP0091 | 20 | DGNPPEVKSVGLGNVNGVTL |
| BcellP0092 | 15 | DGYPIINYEYAIVNN |
| BcellP0093 | 20 | DIKVQFQSGGNNSPAVYLLD |
| BcellP0094 | 15 | DKILVQANEAETTTA |
| BcellP0095 | 20 | DKVLPLVAIVVSVGLAVSYD |
| BcellP0096 | 8 | DLRSSSGC |
| BcellP0097 | 16 | DLSLLGKARKVVVTKD |
| BcellP0098 | 15 | DMAILTGGQVISEEV |
| BcellP0099 | 20 | DMANPMSPVNKSFEIEVTCS |
| BcellP0100 | 12 | DNIGNANIGFGN |
| BcellP0101 | 36 | DNIGNANIGFGNRGDANIGIGNIGDRNLGIGNTGNW |
| BcellP0102 | 10 | DPDKDVDIMV |
| BcellP0103 | 20 | DPDKDVDIMVRDGQLTIKAE |
| BcellP0104 | 15 | DPRYNQLLGPLPFRH |
| BcellP0105 | 20 | DQVHFQPLPPAVVKLSDALI |
| BcellP0106 | 15 | DRFADFPALPLDPSA |
| BcellP0107 | 12 | DRNLGIGNTGNW |
| BcellP0108 | 25 | DRYRHLVALSITDFGAAGPRSSWRA |
| BcellP0109 | 15 | DSIYYVDANASIQEM |
| BcellP0110 | 15 | DTLNGGEYTVFAPTN |
| BcellP0111 | 8 | DTLNKVDA |
| BcellP0112 | 10 | DTVIYSKYGG |
| BcellP0113 | 15 | DVGSLNGTYVNREPV |
| BcellP0114 | 15 | DVVQHVARRPGESPP |
| BcellP0115 | 20 | DWYSPACGKAGCQTYKWETF |
| BcellP0116 | 20 | DWYSPACGKAGCQTYKWETL |
| BcellP0117 | 24 | EAAASAIQGNVTSIHSLLDEGKQS |
| BcellP0118 | 8 | EAEGGTWR |
| BcellP0119 | 15 | EAEHQAIVRDVLAAG |
| BcellP0120 | 8 | EAGPGVAG |
| BcellP0121 | 15 | EDEMKEGRYEVRAEL |
| BcellP0122 | 10 | EDGEKRIPLD |
| BcellP0123 | 8 | EDTLNKVD |
| BcellP0124 | 10 | EEYLILSARD |
| BcellP0125 | 17 | EGDEATGANIVKVALEA |
| BcellP0126 | 10 | EGDTVIYSKY |
| BcellP0127 | 8 | EGGTWRIG |
| BcellP0128 | 15 | EGMRFDKGYISGYFV |
| BcellP0129 | 15 | EGWGKSPGFGTTVDF |
| BcellP0130 | 15 | EGWWPTLIGLAMNDS |
| BcellP0131 | 15 | EHLKLNGKVLAAMYQ |
| BcellP0132 | 10 | EIKYNGEEYL |
| BcellP0133 | 15 | EISTNIRQAGVQYSR |
| BcellP0134 | 16 | EKASVPGGGDMGGMDF |
| BcellP0135 | 8 | EKFVKEQR |
| BcellP0136 | 10 | EKRIPLDVAE |
| BcellP0137 | 15 | EKVAKEAADEAKAKL |
| BcellP0138 | 15 | EMIEAVGYRSWPRYF |
| BcellP0139 | 15 | ENYIAQTRDKFLSAA |
| BcellP0140 | 13 | EQQWNFAGIEAAA |
| BcellP0141 | 15 | ESGTTDNFQRYLQAA |
| BcellP0142 | 15 | ESNTFGLQLELTEGM |
| BcellP0143 | 25 | ETLHERIKVTERRLLVAAVAALATH |
| BcellP0144 | 15 | ETTTASGLVIPDTAK |
| BcellP0145 | 26 | ETTTASGLVIPDTAKEKPQEGTVVAV |
| BcellP0146 | 10 | EYLILSARDV |
| BcellP0147 | 15 | FCGRIHTRYSSAYEL |
| BcellP0148 | 15 | FEETFEVTAAAPVAV |
| BcellP0149 | 15 | FGDVDAHGAMIRAQA |
| BcellP0150 | 8 | FGHQVGDG |
| BcellP0151 | 15 | FGLQLELTEGMRFDK |
| BcellP0152 | 15 | FLGGFAGLPSLGFGN |
| BcellP0153 | 20 | FPGWQPGMPTIPTAPPTTPV |
| BcellP0154 | 15 | FPHHPIHTVPSEEVL |
| BcellP0155 | 15 | FPSFAGLRPTFDTRL |
| BcellP0156 | 22 | FPVVTHDEVLRLVGRRRLWGRG |
| BcellP0157 | 15 | FRMELLSLPQDEWAG |
| BcellP0158 | 20 | FSRPGLPVEYLQVPSPSMGR |
| BcellP0159 | 20 | GAAILVAGLSGCSSNKSTTG |
| BcellP0160 | 20 | GAKGKAALLRELSDVVPNLN |
| BcellP0161 | 20 | GANIPAEFLENFVRSSNLKF |
| BcellP0162 | 15 | GANPLGLKRGIEKAV |
| BcellP0163 | 20 | GASDAYLSEGDMAAHKGLMN |
| BcellP0164 | 8 | GASDLRSS |
| BcellP0165 | 15 | GASVTVTGQGNSLKV |
| BcellP0166 | 20 | GCQTYKWETFLTSELPQWLS |
| BcellP0167 | 15 | GDDDRLHGMLTDRDI |
| BcellP0168 | 15 | GDLPTIGTAVSARNT |
| BcellP0169 | 15 | GDLVGPGCAEYAAAN |
| BcellP0170 | 20 | GDLVGPGCAEYAAANPTGPA |
| BcellP0171 | 10 | GDTVIYSKYG |
| BcellP0172 | 13 | GDVQPAEVVAAAR |
| BcellP0173 | 8 | GEAGPGVA |
| BcellP0174 | 10 | GEEYLILSAR |
| BcellP0175 | 10 | GEKRIPLDVA |
| BcellP0176 | 15 | GELDNRGSQFYLAMY |
| BcellP0177 | 20 | GEQFMQWFLQEQIEEVALMA |
| BcellP0178 | 13 | GFASKTPANQAIS |
| BcellP0179 | 19 | GFKVRPSFSFFAVGPDGMP |
| BcellP0180 | 15 | GFLMPPSDGSGVTPG |
| BcellP0181 | 15 | GFSEIMRSTLEKDNT |
| BcellP0182 | 10 | GGTEIKYNGE |
| BcellP0183 | 8 | GGTWRIGY |
| BcellP0184 | 10 | GGVAVIKAGA |
| BcellP0185 | 7 | GHNAVFN |
| BcellP0186 | 8 | GHNAVFNC |
| BcellP0187 | 8 | GHQVGDGE |
| BcellP0188 | 8 | GHWRCPPR |
| BcellP0189 | 12 | GIGNIGDRNLGI |
| BcellP0190 | 14 | GKPTEKHIQIRSTN |
| BcellP0191 | 24 | GKQSLTKLAAAWGGSGSEAYQGVQ |
| BcellP0192 | 20 | GLRAQDDFSGWDINTPAFEW |
| BcellP0193 | 20 | GLRAQDDYNGWDINTPAFEW |
| BcellP0194 | 10 | GLRNVAAGAN |
| BcellP0195 | 8 | GMSQDPVA |
| BcellP0196 | 15 | GNFERISGDLKTQID |
| BcellP0197 | 20 | GNGGMVTGCAETPGCVAYIG |
| BcellP0198 | 15 | GNKASFLDQVHFQPL |
| BcellP0199 | 12 | GNPNRPDGGILT |
| BcellP0200 | 7 | GPAFHER |
| BcellP0201 | 15 | GPGQEGLDQYGSIPL |
| BcellP0202 | 8 | GPGVAGSG |
| BcellP0203 | 16 | GPKGRNVVLEKKWGAP |
| BcellP0204 | 21 | GPRLYGEMTMQGTRKPRPSGP |
| BcellP0205 | 20 | GPSSDPAWERNDPTQQIPKL |
| BcellP0206 | 16 | GRWDEDGEKRIPLDVA |
| BcellP0207 | 8 | GSGASDLR |
| BcellP0208 | 16 | GSINTGWFNTGNANTG |
| BcellP0209 | 14 | GSKPPSGSPETGAG |
| BcellP0210 | 8 | GSKVPEDT |
| BcellP0211 | 25 | GSLIFAAVLVMLIAVLARLMMRGWR |
| BcellP0212 | 20 | GSRDCVIPVEQARSFVERLR |
| BcellP0213 | 50 | GTRQTLQGASVTVTGQGNSLKVGNADVVCGGVSTANATVYMIDSVLMPPA |
| BcellP0214 | 10 | GTVVAVGPGR |
| BcellP0215 | 15 | GTVVAVGPGRWDEDG |
| BcellP0216 | 16 | GTVVAVGPGRWDEDGE |
| BcellP0217 | 8 | GVAGSGAS |
| BcellP0218 | 15 | GVIFFFNPGELLPEA |
| BcellP0219 | 15 | GVPSRLGYLLDLAPK |
| BcellP0220 | 20 | GVSTANATVYMIDSVLMPPA |
| BcellP0221 | 10 | GVTLLQAAPT |
| BcellP0222 | 15 | GVYKVCKGLEKIPLL |
| BcellP0223 | 8 | GYFGHQVG |
| BcellP0224 | 15 | GYIPILGAPWLADLV |
| BcellP0225 | 15 | GYPVHKPVTAGWNGY |
| BcellP0226 | 15 | HGTCANQCPIVDAGD |
| BcellP0227 | 20 | HPTTTYKAFDWDQAYRKPIT |
| BcellP0228 | 15 | HQGQGGAKSKGSQQE |
| BcellP0229 | 8 | HQVGDGEA |
| BcellP0230 | 15 | HRSDGSGDTFLFTQY |
| BcellP0231 | 22 | HSLLWIGVFLFLGGMAAGGCNS |
| BcellP0232 | 15 | HVIAGQASPSRIDGT |
| BcellP0233 | 16 | HVTLYLGNGQMELPNK |
| BcellP0234 | 15 | HWLRPITPTFRPSWP |
| BcellP0235 | 8 | HWRCPPRR |
| BcellP0236 | 15 | IADIEAYPQWISEYK |
| BcellP0237 | 15 | IAFLAGCSSTKPVSQ |
| BcellP0238 | 20 | IAIGGAATGIAAVLTDGNPP |
| BcellP0239 | 21 | IALAISAQQVNYNLPGVSEHL |
| BcellP0240 | 20 | IAVAGCSAGGHLSALAGLTA |
| BcellP0241 | 15 | IELPKKAAPAKKAAP |
| BcellP0242 | 15 | IGLSMAGSSAMILAA |
| BcellP0243 | 8 | IGYFGHQV |
| BcellP0244 | 10 | IKPLEDKILV |
| BcellP0245 | 9 | IKYNGEEYL |
| BcellP0246 | 10 | IKYNGEEYLI |
| BcellP0247 | 15 | ILKDESYKVTGTAPI |
| BcellP0248 | 20 | ILTVSVAVSEGKPTEKHIQI |
| BcellP0249 | 25 | IMIVSLGVTASSFLFINGVAFLIPR |
| BcellP0250 | 21 | INFNCEVWSNVSETISGPRLY |
| BcellP0251 | 24 | IPVVSVTKSVGFQLRGQSGPTTVK |
| BcellP0252 | 20 | IQMSDPAYNINISLPSYYPD |
| BcellP0253 | 15 | IRGSVTPAVSQFNAR |
| BcellP0254 | 15 | IRLAKCGPWHRGGWD |
| BcellP0255 | 15 | IRQAGVQYSRADEEQ |
| BcellP0256 | 15 | ISGDLKTQIDQVEST |
| BcellP0257 | 15 | ISMIDGPAPDGYPII |
| BcellP0258 | 15 | ITSAGRHPDSDIFLD |
| BcellP0259 | 10 | IVAGGGVTLL |
| BcellP0260 | 15 | IVMSTNNDDPAGACR |
| BcellP0261 | 15 | IWKLIKDMLILPYLD |
| BcellP0262 | 10 | IYSKYGGTEI |
| BcellP0263 | 15 | KAMLFDGRSGEPFPY |
| BcellP0264 | 15 | KAVEKVTETLLKGAK |
| BcellP0265 | 8 | KFVKEQRE |
| BcellP0266 | 15 | KGLMNIALAISAQQV |
| BcellP0267 | 20 | KMVAPVLEEIATERATDLTV |
| BcellP0268 | 15 | KPEKEKASVPGGGDM |
| BcellP0269 | 10 | KPLEDKILVQ |
| BcellP0270 | 20 | KQDPEGWGKSPGFGTTVDFP |
| BcellP0271 | 15 | KQELDEISTNIRQAG |
| BcellP0272 | 10 | KRIPLDVAEG |
| BcellP0273 | 11 | KTIAYDEEARR |
| BcellP0274 | 15 | KTINLCAPDDPICTG |
| BcellP0275 | 15 | KTLDAAIGKLLDNDK |
| BcellP0276 | 8 | KTNSSLLT |
| BcellP0277 | 15 | KTQIDQVESTAGSLQ |
| BcellP0278 | 15 | KVALEAPLKQIAFNS |
| BcellP0279 | 10 | KVNIKPLEDK |
| BcellP0280 | 8 | KVPEDTLN |
| BcellP0281 | 10 | KVTLGPKGRN |
| BcellP0282 | 11 | KVVIDGKDQNV |
| BcellP0283 | 5 | KWDAT |
| BcellP0284 | 10 | KYGGTEIKYN |
| BcellP0285 | 10 | KYNGEEYLIL |
| BcellP0286 | 15 | LAALPDFLGGFAGLP |
| BcellP0287 | 20 | LAGGAATAGAFSRPGLPVEY |
| BcellP0288 | 10 | LAKLAGGVAV |
| BcellP0289 | 15 | LAPDTVPLAGVVLAG |
| BcellP0290 | 15 | LAQEAGNFERISGDL |
| BcellP0291 | 24 | LARTISEAGQAMASTEGNVTGMFA |
| BcellP0292 | 15 | LASTLTDALKSHGPQ |
| BcellP0293 | 15 | LCKTTSNFIWGQLLL |
| BcellP0294 | 16 | LDEGKQSLTKLAAAWG |
| BcellP0295 | 15 | LDVAEGDTVIYSKYG |
| BcellP0296 | 15 | LEAAGDKKIGVIKVV |
| BcellP0297 | 15 | LEPGVVAEKVRNLPA |
| BcellP0298 | 16 | LEPGVVAEKVRNLPAG |
| BcellP0299 | 20 | LFAAFPSFAGLRPTFDTRLM |
| BcellP0300 | 20 | LFNLWGPAFHERYPNVTITA |
| BcellP0301 | 15 | LFSRVEDVLGLPQNT |
| BcellP0302 | 15 | LGDVGEAFVDSLTSQ |
| BcellP0303 | 15 | LGENGNGGMVTGCAE |
| BcellP0304 | 15 | LGGGVGAFLNALFAG |
| BcellP0305 | 12 | LGLKRGIEKAVE |
| BcellP0306 | 15 | LGWKVSDLKSSTAVI |
| BcellP0307 | 10 | LILSARDVLA |
| BcellP0308 | 15 | LILTSYTSDEAMLDA |
| BcellP0309 | 8 | LKTNSSLL |
| BcellP0310 | 13 | LKTQIDQVESTAG |
| BcellP0311 | 15 | LLAAGVADPVKVTRS |
| BcellP0312 | 15 | LLDIYRKLRPGEPPT |
| BcellP0313 | 15 | LLQAAPTLDELKLEG |
| BcellP0314 | 15 | LMILIATNLLGQNTP |
| BcellP0315 | 15 | LNAMKGDLQSSLGAG |
| BcellP0316 | 8 | LNKVDAAV |
| BcellP0317 | 20 | LNSGQYTVFAPTNAAFSKLP |
| BcellP0318 | 15 | LPADTAAQLTSAGRE |
| BcellP0319 | 15 | LPDWLAANRGLAPGG |
| BcellP0320 | 15 | LPGVSEHLKLNGKVL |
| BcellP0321 | 41 | LPKKAAPAKKAAPAKKAAPAKKAAAKKAPAKKAAAKKVTQK |
| BcellP0322 | 15 | LPLLEKVIGAGKPLL |
| BcellP0323 | 24 | LPVVALCTVSDIVLIAAGIAGFGA |
| BcellP0324 | 15 | LQAHPHAVNGTAMRE |
| BcellP0325 | 20 | LQDSGVHDVAVISEAQAATA |
| BcellP0326 | 15 | LQSLGAEIAVEQAAL |
| BcellP0327 | 20 | LQVPSPSMGRDIKVQFQSGG |
| BcellP0328 | 15 | LRPASCRFVPTCSQY |
| BcellP0329 | 10 | LRPTFDTRLM |
| BcellP0330 | 20 | LRPTFDTRLMRLEDEMKEGR |
| BcellP0331 | 8 | LRSSSGCV |
| BcellP0332 | 15 | LSEGDMAAHKGLMNI |
| BcellP0333 | 12 | LSFTLTGNPNRP |
| BcellP0334 | 15 | LSIVMPVGGQSSFYS |
| BcellP0335 | 20 | LSVVGDPDAPPTMVAVAPVA |
| BcellP0336 | 25 | LTFLGFIGVLLAINLFGNRAIKWAN |
| BcellP0337 | 15 | LTLENADLSLLGKAR |
| BcellP0338 | 20 | LTSELPQWLSANRAVKPTGS |
| BcellP0339 | 15 | LTTYILLPSQDLPLL |
| BcellP0340 | 8 | LVYQTEKF |
| BcellP0341 | 15 | MAEDVRAEIVASVLE |
| BcellP0342 | 15 | MAEMKTDAATLAQEA |
| BcellP0343 | 15 | MAKTIAYDEEARRGL |
| BcellP0344 | 15 | MAKVNIKPLEDKILV |
| BcellP0345 | 13 | MINVQAKPAAAAS |
| BcellP0346 | 20 | MKIRLHTLLAVLTAAPLLLA |
| BcellP0347 | 20 | MKRGLTVAVAGAAILVAGLS |
| BcellP0348 | 24 | MKRGLTVAVAGAAILVAGLSGCSS |
| BcellP0349 | 16 | MLGNAPSVVPNTTLGM |
| BcellP0350 | 16 | MLGTGTPNRARINFNC |
| BcellP0351 | 14 | MLQDMAILTGGQVI |
| BcellP0352 | 20 | MLVQHLLDRDLRVEIPGVDT |
| BcellP0353 | 15 | MNIALAISAQQVNYN |
| BcellP0354 | 23 | MRASPAERVDGAYAGAGPHTQSV |
| BcellP0355 | 32 | MRFAQPSALSRFSALTRDWFTSTFAAPTAAQA |
| BcellP0356 | 15 | MSFVTIQPVVLAAAT |
| BcellP0357 | 15 | MSPVNKSFEIEVTCS |
| BcellP0358 | 15 | MTAHTHDGTRTWRTG |
| BcellP0359 | 20 | MTDVSRKIRAWGRRLMIGTA |
| BcellP0360 | 40 | MTDVSRKIRAWGRRLMIGTAAAVVLPGLVGLAGGAATAGA |
| BcellP0361 | 16 | MTEQQWNFAGIEAAAS |
| BcellP0362 | 20 | MTEQQWNFAGIEAAASAIQG |
| BcellP0363 | 15 | MTLLELSDFVKKFEE |
| BcellP0364 | 11 | MVNKSRMMPAV |
| BcellP0365 | 15 | MWELYLAYSEAGFRS |
| BcellP0366 | 15 | MWLSPNGTIRNILGG |
| BcellP0367 | 5 | NAAFS |
| BcellP0368 | 10 | NAASIAGLFL |
| BcellP0369 | 15 | NAFLTALTNAGIAYD |
| BcellP0370 | 20 | NAVFNFPPNGTHSWEYWGAQ |
| BcellP0371 | 20 | NDPQYQAELPEGSDTSVDAV |
| BcellP0372 | 20 | NDPTQQIPKLVANNTRLWVY |
| BcellP0373 | 10 | NGEEYLILSA |
| BcellP0374 | 15 | NGEEYLILSARDVLA |
| BcellP0375 | 20 | NGVTLGYTSGTCQGNASATK |
| BcellP0376 | 12 | NIGFGNRGDANI |
| BcellP0377 | 10 | NIKPLEDKIL |
| BcellP0378 | 20 | NITSATYQSAIPPRGTQAVV |
| BcellP0379 | 8 | NKVDAAVA |
| BcellP0380 | 8 | NNPELTTL |
| BcellP0381 | 20 | NNSPAVYLLDGLRAQDDYNG |
| BcellP0382 | 15 | NPLGLKRGIEKAVEK |
| BcellP0383 | 8 | NPQVNLVD |
| BcellP0384 | 8 | NPTGPASV |
| BcellP0385 | 14 | NRYVELKRGEGKGA |
| BcellP0386 | 9 | NTFGLQLEL |
| BcellP0387 | 15 | NTPAIAVNEAEYGEM |
| BcellP0388 | 16 | NVTSIHSLLDEGKQSL |
| BcellP0389 | 7 | NVVGTRQ |
| BcellP0390 | 15 | PAEFLENFVRSSNLK |
| BcellP0391 | 15 | PAGGAYSMYTNWEQD |
| BcellP0392 | 15 | PDRSAERCGSPAWDL |
| BcellP0393 | 15 | PDTAKEKPQEGTVVA |
| BcellP0394 | 8 | PEDTLNKV |
| BcellP0395 | 15 | PETINYRTLKPEKDG |
| BcellP0396 | 15 | PFNVNLKLQFLHDAF |
| BcellP0397 | 10 | PGFGDRRKAM |
| BcellP0398 | 15 | PGGTTTLVEHMVGIL |
| BcellP0399 | 15 | PGQGAYAAANSWVDV |
| BcellP0400 | 8 | PGVAGSGA |
| BcellP0401 | 15 | PGYPVAGQVWEATAT |
| BcellP0402 | 13 | PHVKPAALAEQPG |
| BcellP0403 | 15 | PILGAPWLADLVRRS |
| BcellP0404 | 20 | PIPVPIIIPPFPGWQPGMPT |
| BcellP0405 | 10 | PKGRNVVLEK |
| BcellP0406 | 15 | PLDTPPAPAPPPFRL |
| BcellP0407 | 10 | PLGLKRGIEK |
| BcellP0408 | 10 | PLKQIAFNSG |
| BcellP0409 | 13 | PNGTHSWEYWGAQ |
| BcellP0410 | 15 | PPGYDITALLQAHPH |
| BcellP0411 | 8 | PPRRRAGR |
| BcellP0412 | 15 | PPTPISVIFRSDKSG |
| BcellP0413 | 8 | PQVNLVDT |
| BcellP0414 | 8 | PRRRAGRC |
| BcellP0415 | 15 | PSDLGGNNLPAKFLE |
| BcellP0416 | 20 | PSLIGLAMGDAGGYKAADMW |
| BcellP0417 | 9 | PSMGRDIKV |
| BcellP0418 | 24 | PSWGLVVTMFAWGYLLDHVGERMV |
| BcellP0419 | 20 | PTNAAFSKLPASTIDELKTN |
| BcellP0420 | 15 | PVAGQVWEATATVNA |
| BcellP0421 | 9 | PVEYLQVPS |
| BcellP0422 | 20 | PVGGQSSFYSDWYSPACGKA |
| BcellP0423 | 15 | PVIAENRAELMILIA |
| BcellP0424 | 15 | QAQLISSQAQQGGQQ |
| BcellP0425 | 15 | QAQYVAQLHVWARRE |
| BcellP0426 | 20 | QDAYNAAGGHNAVFNFPPNG |
| BcellP0427 | 15 | QDPEGWGKSPGFGTT |
| BcellP0428 | 4 | QDPV |
| BcellP0429 | 22 | QDVGNYDAFISIRAAIRAIESA |
| BcellP0430 | 15 | QEAGNFERISGDLKT |
| BcellP0431 | 16 | QEIENSDSDYDREKLQ |
| BcellP0432 | 15 | QGQWRGAAGTAAQAA |
| BcellP0433 | 24 | QGVQQKWDATATELNNALQNLART |
| BcellP0434 | 10 | QIAATAAISA |
| BcellP0435 | 13 | QKVQAAGNNMAQT |
| BcellP0436 | 15 | QNAGGTHPTTTYKAF |
| BcellP0437 | 15 | QPACRKAGCQTYKWE |
| BcellP0438 | 20 | QQFIYAGSLSALLDPSQGMG |
| BcellP0439 | 13 | QQKWDATATELNN |
| BcellP0440 | 15 | QSGGNNSPAVYLLDG |
| BcellP0441 | 15 | QSGLGDNGYIPIPDE |
| BcellP0442 | 19 | QSIGDLIAEAMDKVGNEGV |
| BcellP0443 | 19 | QSIGDLIAFAMDKVGNEGV |
| BcellP0444 | 15 | QSSFYSDWYSPACGK |
| BcellP0445 | 15 | QSVCPILAEPGGSFN |
| BcellP0446 | 8 | QTEKFVKE |
| BcellP0447 | 15 | QVESTAGSLQGQWRG |
| BcellP0448 | 8 | QVGDGEAG |
| BcellP0449 | 15 | QVGSQFGPYQLLRLL |
| BcellP0450 | 15 | QVVLGWKVSDLKSST |
| BcellP0451 | 8 | RAGRCPPR |
| BcellP0452 | 13 | RALKRRNARTKAR |
| BcellP0453 | 8 | RCPPRRRA |
| BcellP0454 | 20 | RDGQLTIKAERTEQKDFDGR |
| BcellP0455 | 10 | RFDKGYISGY |
| BcellP0456 | 12 | RGDANIGIGNIG |
| BcellP0457 | 15 | RGEHRDEHTQDAGDK |
| BcellP0458 | 15 | RGLGEAQLGNSSGNF |
| BcellP0459 | 15 | RGLRAAAEAGVRLAF |
| BcellP0460 | 24 | RGVAGKVVAGIGILAIRWRLPSGT |
| BcellP0461 | 15 | RHGLSSLGWNLCRIF |
| BcellP0462 | 15 | RIFYKDAFAKHQELF |
| BcellP0463 | 8 | RIGYFGHQ |
| BcellP0464 | 15 | RKVVVTKDETTIVEG |
| BcellP0465 | 10 | RNLPAGHGLN |
| BcellP0466 | 12 | RPGLDELSFTLT |
| BcellP0467 | 24 | RPGLDELSFTLTGNPNRPDGGILT |
| BcellP0468 | 8 | RRAGRCPP |
| BcellP0469 | 15 | RRFPLPDVPWTRFFG |
| BcellP0470 | 10 | RRKAMLQDMA |
| BcellP0471 | 8 | RRRAGRCP |
| BcellP0472 | 15 | RSCPGYTLDYNANGS |
| BcellP0473 | 8 | RSSSGCVT |
| BcellP0474 | 20 | RTEQKDFDGRSEFAYGSFVR |
| BcellP0475 | 15 | SAAAKNTPGSITYNE |
| BcellP0476 | 15 | SAAIGLSMAGSSAMI |
| BcellP0477 | 15 | SAERCGSPAWDLPTV |
| BcellP0478 | 15 | SAFAAKAGLMRHTIG |
| BcellP0479 | 20 | SAMILAAYHPQQFIYAGSLS |
| BcellP0480 | 8 | SDLRSSSG |
| BcellP0481 | 14 | SEFAYGSFVRTVSL |
| BcellP0482 | 15 | SEFDVILEAAGDKKI |
| BcellP0483 | 15 | SEWRSPADTSPRPLP |
| BcellP0484 | 8 | SGASDLRS |
| BcellP0485 | 20 | SGETTTAAGTTASPGAASGP |
| BcellP0486 | 8 | SKVPEDTL |
| BcellP0487 | 15 | SKVSTVKDLLPLLEK |
| BcellP0488 | 10 | SKYGGTEIKY |
| BcellP0489 | 5 | SNNPE |
| BcellP0490 | 15 | SPHPLLTHAVEQTGR |
| BcellP0491 | 22 | SPYRGSSILWRIHAASALLMMP |
| BcellP0492 | 12 | SSDPAWERNDPT |
| BcellP0493 | 8 | SSGCVTGH |
| BcellP0494 | 8 | SSSGCVTG |
| BcellP0495 | 8 | STANATVY |
| BcellP0496 | 11 | STEGNVTGMFA |
| BcellP0497 | 15 | SVPANVSRRAKVDVL |
| BcellP0498 | 5 | SVQGM |
| BcellP0499 | 15 | TAALRAAEDDCPPPG |
| BcellP0500 | 20 | TAIGVWGARHASDFLSATAK |
| BcellP0501 | 15 | TAQTLQAFLHWAITD |
| BcellP0502 | 20 | TASPGAASGPKVVIDGKDQN |
| BcellP0503 | 15 | TAVVLLCCSGVATAA |
| BcellP0504 | 15 | TCELFSRVEDVLGLP |
| BcellP0505 | 15 | TDELNQQRDDITRAI |
| BcellP0506 | 15 | TDVIPETDGAEKGPT |
| BcellP0507 | 8 | TEKFVKEQ |
| BcellP0508 | 9 | TFGLQLELT |
| BcellP0509 | 15 | TGGGGGSGFSNSGSG |
| BcellP0510 | 8 | TGHWRCPP |
| BcellP0511 | 15 | TGNMISNQAKYVSDT |
| BcellP0512 | 33 | TGPASVQGMSQDPVAVAASNNPELTTLTAALSG |
| BcellP0513 | 13 | TGVYEDLLAAGVA |
| BcellP0514 | 20 | THSWEYWGAQLNAMKGDLQS |
| BcellP0515 | 20 | THSWEYWGAQLNAMKPDLQR |
| BcellP0516 | 15 | TIEQLLTIPLAKELA |
| BcellP0517 | 9 | TKRRITPKD |
| BcellP0518 | 11 | TLLKGAKEVET |
| BcellP0519 | 8 | TLNKVDAA |
| BcellP0520 | 15 | TLQAFLHWAITDGNK |
| BcellP0521 | 20 | TLVVNKIRGTFKSVAVKAPG |
| BcellP0522 | 15 | TNDGVIFFFNPGELL |
| BcellP0523 | 15 | TNFFGINTIPIALTE |
| BcellP0524 | 15 | TPPAPAPPPFRLPLL |
| BcellP0525 | 15 | TSELPQWLSANRAVK |
| BcellP0526 | 15 | TSEQIVERFGFSEIM |
| BcellP0527 | 15 | TTEAVVADKPEKEKA |
| BcellP0528 | 25 | TTIFPDHVVGRVGALLALAALIHRD |
| BcellP0529 | 17 | TTLGMHCGSFGSAPSNG |
| BcellP0530 | 17 | TTPKNPTKRRITPKDVI |
| BcellP0531 | 15 | TVIAATAGRNHLKNV |
| BcellP0532 | 10 | TVIYSKYGGT |
| BcellP0533 | 10 | TVLAQALVRE |
| BcellP0534 | 20 | TVSLPVGADEDDIKATYDKG |
| BcellP0535 | 10 | TVVAVGPGRW |
| BcellP0536 | 15 | TWQTKSGGTRTGNVT |
| BcellP0537 | 8 | VAEAEGGT |
| BcellP0538 | 10 | VAEKVRNLPA |
| BcellP0539 | 24 | VAGGGVTLLQAAPTLDELKLEGDE |
| BcellP0540 | 20 | VAGLSGCSSNKSTTGSGETT |
| BcellP0541 | 8 | VAGSGASD |
| BcellP0542 | 15 | VAKVNIKPLEDKILV |
| BcellP0543 | 15 | VALFGEPSSGFSSML |
| BcellP0544 | 20 | VANNTRLWVYCGNGTPNELG |
| BcellP0545 | 10 | VAVGPGRWDE |
| BcellP0546 | 15 | VCAVDMPYLTVELIE |
| BcellP0547 | 15 | VFGGAASCAAPIQAD |
| BcellP0548 | 8 | VGDGEAGP |
| BcellP0549 | 15 | VGNVGLSTIAGRLLG |
| BcellP0550 | 15 | VGPGRWDEDGEKRIP |
| BcellP0551 | 15 | VGQVTATMGQLQQLV |
| BcellP0552 | 15 | VHNVAALPGAAYCEM |
| BcellP0553 | 10 | VIYSKYGGTE |
| BcellP0554 | 20 | VKRGLTVAVAGAAILVAGLS |
| BcellP0555 | 15 | VLCNGGSPTTQQALA |
| BcellP0556 | 15 | VLLESMKMEIPVLAE |
| BcellP0557 | 15 | VMPVGGQSSFYSDWY |
| BcellP0558 | 10 | VNIKPLEDKI |
| BcellP0559 | 7 | VNKSFEI |
| BcellP0560 | 8 | VPEDTLNK |
| BcellP0561 | 15 | VQYSRADEEQQQALS |
| BcellP0562 | 15 | VRERMTTQDVEAITP |
| BcellP0563 | 15 | VRFQEAANKQKQELD |
| BcellP0564 | 15 | VRRRHGLSSLGWNLC |
| BcellP0565 | 15 | VSVAVSEGKPTEKHI |
| BcellP0566 | 14 | VTFHYQNKYKVPAN |
| BcellP0567 | 8 | VTGHWRCP |
| BcellP0568 | 7 | VTGSVVC |
| BcellP0569 | 20 | VTGSVVCTTAAGNVNIAIGG |
| BcellP0570 | 24 | VTSIHSLLDEGKQSLTKLAAAWGG |
| BcellP0571 | 20 | VTVTFMLVWLINHHGWSVAQ |
| BcellP0572 | 15 | VTVTGQGNSLKVGNA |
| BcellP0573 | 10 | VVAVGPGRWD |
| BcellP0574 | 25 | VVHPAVVQANRVRTWLLAVSNVFGQ |
| BcellP0575 | 10 | VVLEKKWGAP |
| BcellP0576 | 8 | VYQTEKFV |
| BcellP0577 | 7 | WDEDGEK |
| BcellP0578 | 15 | WDEDGEKRIPLDVAE |
| BcellP0579 | 20 | WDINTPAFEWYYQSGLSIVM |
| BcellP0580 | 15 | WGPSSDPAWERNDPT |
| BcellP0581 | 20 | WGRRLMIGTAAAVVLPGLVG |
| BcellP0582 | 15 | WISEYKEVEILEADD |
| BcellP0583 | 21 | WLKLGLVEFGGVAKLNAEVMS |
| BcellP0584 | 15 | WLSANRAVKPTGSAA |
| BcellP0585 | 8 | WRCPPRRR |
| BcellP0586 | 8 | WRIGYFGH |
| BcellP0587 | 15 | WRSLDVEMTAVQRSF |
| BcellP0588 | 15 | WSFAVGKQLNMAQII |
| BcellP0589 | 20 | YAAANPTGPASVQGMSQDPV |
| BcellP0590 | 15 | YDEEARRGLERGLNA |
| BcellP0591 | 15 | YDREKLQERLAKLAG |
| BcellP0592 | 15 | YEEQMQDAFETGVMF |
| BcellP0593 | 21 | YEKIGAELVKEVAKKTDDVAG |
| BcellP0594 | 20 | YEVRAELPGVDPDKDVDIMV |
| BcellP0595 | 8 | YFGHQVGD |
| BcellP0596 | 10 | YGGTEIKYNG |
| BcellP0597 | 15 | YHVVAGQTSPANVVG |
| BcellP0598 | 15 | YIGISFLDQASQRGL |
| BcellP0599 | 10 | YILLVSSKVS |
| BcellP0600 | 10 | YISGYFVTDP |
| BcellP0601 | 10 | YLILSARDVL |
| BcellP0602 | 8 | YMIDSVLM |
| BcellP0603 | 15 | YNANGSGAGVTQFLN |
| BcellP0604 | 10 | YNGEEYLILS |
| BcellP0605 | 15 | YNGEEYLILSARDVL |
| BcellP0606 | 9 | YQGVQQKWD |
| BcellP0607 | 8 | YQTEKFVK |
| BcellP0608 | 15 | YRADSVERLIAAHLM |
| BcellP0609 | 15 | YRLERPLLFALQCMP |
| BcellP0610 | 10 | YSKYGGTEIK |
| BcellP0611 | 15 | YTFKEDEYPSTAYLQ |
| BcellP0612 | 20 | YYQSGLSIVMPVGGQSSFYS |
