# Supplementary material for: Genomic Analysis of a Mycobacterium Bovis Bacillus Calmette-Guérin Strain Isolated from an Adult Patient with Pulmonary Tuberculosis
Source: PLoS One. 2015 Apr 13;10(4):e0122403. doi: 10.1371/journal.pone.0122403 (PMC4395146; doi:10.1371/journal.pone.0122403)
Supplement: S4 Table — (DOC) [file pone.0122403.s004.doc]

**Table S4 Details of the lost epitopes by BCG 3281 alone.**

| **Epitope** | **Gene** | **Cause** |
| --- | --- | --- |
| TcellP1313 | Rv0164 | frameshift |
| TcellP1499 | Rv0286 | frameshift |
| TcellP1946 | Rv0298 | frameshift |
| TcellP1312 | Rv0747 | lost |
| BcellP0168 | Rv0916c | frameshift |
| BcellP0356 | Rv0916c | frameshift |
| BcellP0060 | Rv0956 | frameshift |
| TcellP0278 | Rv1361c | frameshift |
| TcellP0445 | Rv1844c | lost |
| TcellP0582 | Rv1844c | lost |
| TcellP0911 | Rv1870c | frameshift |
| TcellP0067 | Rv1877 | frameshift |
| TcellP0174 | Rv1877 | frameshift |
| BcellP0359 | Rv1886c | frameshift |
| BcellP0360 | Rv1886c | frameshift |
| TcellP1315 | Rv1886c | frameshift |
| TcellP1316 | Rv1886c | frameshift |
| TcellP1504 | Rv2290 | frameshift |
| BcellP0289 | Rv2453c | frameshift |
| TcellP0558 | Rv2526 | frameshift |
| TcellP1328 | Rv2526 | frameshift |
| TcellP0933 | Rv2628 | frameshift |
| TcellP0968 | Rv2628 | frameshift |
| TcellP1310 | Rv2628 | frameshift |
| TcellP1311 | Rv2628 | frameshift |
| TcellP1432 | Rv2628 | frameshift |
| TcellP1392 | Rv2770c | frameshift |
| BcellP0231 | Rv2994 | frameshift |
| BcellP0249 | Rv2994 | frameshift |
| BcellP0418 | Rv2994 | frameshift |
| BcellP0491 | Rv2994 | frameshift |
| BcellP0571 | Rv2994 | frameshift |
| TcellP1442 | Rv2994 | frameshift |
| TcellP1947 | Rv2994 | frameshift |
| TcellP1231 | Rv3330 | frameshift |
| TcellP1605 | Rv3378c | frameshift |
| TcellP1186 | Rv3407 | frameshift |
| TcellP0414 | Rv3425 | lost |
| TcellP0447 | Rv3425 | lost |
| TcellP0534 | Rv3425 | lost |
| TcellP1012 | Rv3425 | lost |
| TcellP1257 | Rv3425 | lost |
| TcellP1566 | Rv3425 | lost |
| TcellP1604 | Rv3689 | frameshift |
